# Synthesis, Crystal and Electronic Structures of a Thiophosphinoyl‐ and Amino‐Substituted Metallated Ylide

**DOI:** 10.1002/open.202100187

**Published:** 2021-09-27

**Authors:** Mike Jörges, Alexander Kroll, Leif Kelling, Richard Gauld, Bert Mallick, Stefan M. Huber, Viktoria H. Gessner

**Affiliations:** ^1^ Faculty of Chemistry and Biochemistry Ruhr-University Bochum Universitätsstraße 150 44801 Bochum Germany

**Keywords:** alkali metals, lithium, potassium, structure elucidation, ylide ligands

## Abstract

α‐Metallated ylides have revealed themselves to be versatile reagents for the introduction of ylide groups. Herein, we report the synthesis of the thiophosphinoyl and piperidyl (Pip) substituted α‐metallated ylide [Ph_2_(Pip)P=C−P(S)Ph_2_]M (M=Li, Na, K) through a four‐step synthetic procedure starting from diphenylmethylphosphine sulfide. Metallation of the ylide intermediate was successfully accomplished with different alkali metal bases delivering the lithium, sodium and potassium salts, the latter isolable in high yields. Structure analyses of the lithium and potassium compounds in the solid state with and without crown ether revealed different aggregates (monomer, dimer and hexamer) with the metals coordinated by the thiophosphoryl moiety and ylidic carbon atom. Although the piperidyl group does not coordinate to the metal, it significantly contributes to the stability of the yldiide by charge delocalization through negative hyperconjugation.

## Graphical abstract

**The synthesis and structures** of the thiophosphoryl‐ and amino‐substituted metallated ylide [Ph_2_(Pip)P=C−P(S)Ph_2_]M (M=Li, Na, K) are reported. The structures of the alkali metal salts (hexamer, dimer, monomer) reflect the stabilizing effects responsible for charge delocalization and show that the piperidyl moiety, albeit not coordinating to the metal, decisively contributes to the overall stability of the yldiide.

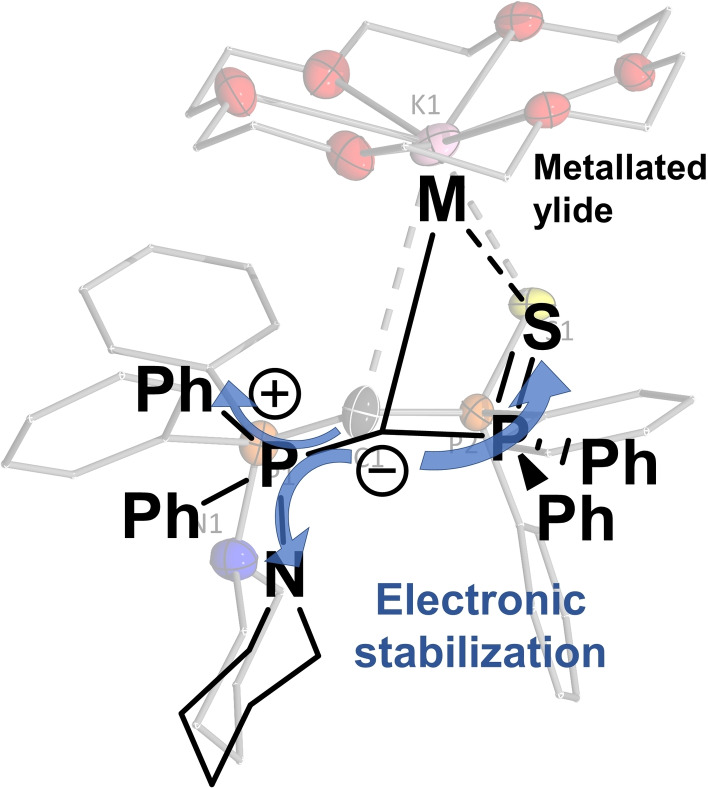

Phosphorus ylides are valuable reagents in chemical synthesis and have been applied in various research directions since many years. Apart from their utilization in organic synthesis (e. g., in olefination reactions), they have also been used as ligands in coordination chemistry and as strong donor substituents in organic materials and main group compounds.^[1]^ Typically, ylides are synthesized from the corresponding phosphonium salts by deprotonation. However, more recently, the introduction of ylide moieties by a salt metathesis approach using α‐metallated ylides has become a viable alternative.^[2]^ The prerequisite for this approach is the accessibility of yldiide species. The first metallated ylide was already reported by Corey and Kang in 1982, who prepared lithium triphenylphosphonium methyldiide, Ph_3_PC(H)Li, by lithiation of phosphonium methylide.^[3]^ While this yldiide was only used as an in situ‐prepared reagent and its existence had also been questioned by Schlosser and co‐workers,^[4]^ Bestmann and Schmidt succeeded in the isolation of the cyano‐stabilized compound **A** (Figure 1), whose alkali metal salts were also later structurally characterized.^[5]^ Since then, a number of metallated ylides (compounds **B‐E**) have been isolated^[6]^ – partly also on a gram scale – and applied in ylide transfer reactions.^[7]^ Our group, for example, isolated the sulfonyl‐substituted compounds **D** and used them for the stabilization of low‐valent main group species^[8]^ as well as in the synthesis of ylide‐substituted phosphines, which revealed themselves to be powerful ligands for homogeneous catalysis.[^9^, ^10^]

**Figure 1:**
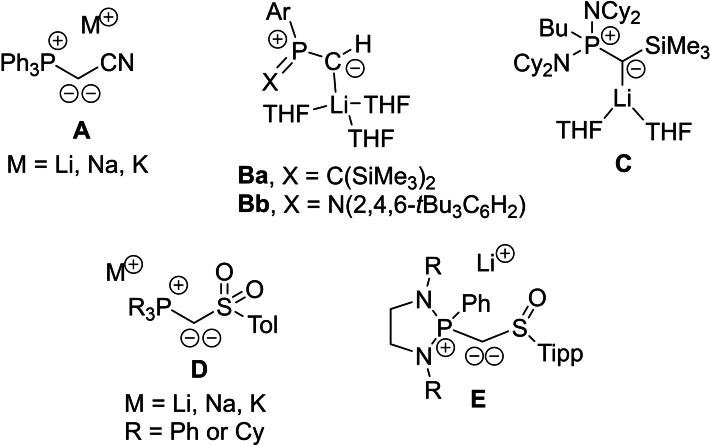
Isolated metallated ylides.

In order to expand the portfolio of readily accessible metallated ylides for their use in main group chemistry, we became interested in the preparation of further readily isolable and storable yldiides. Whereas sulfonyl and sulfinyl groups have already been proven to be suitable groups for stabilizing the high negative charge at the ylidic carbon atom, we also expected P^V^ substituents to being able to provide sufficient stabilization. Thus, we targeted the synthesis of a thiophosphoryl‐substituted compound and selected compound **Y‐H** (Scheme 1), since we expected the additional amino substituent at the phosphonium unit to be beneficial for metal coordination and hence for isolation of the alkali metal yldiides. Here, we report our findings.

**Scheme 1:**
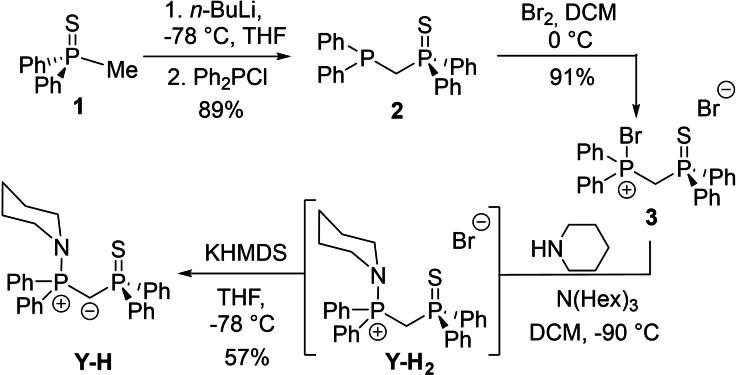
Synthesis route for **Y‐H** starting from chlorodiphenylphosphine sulfide (**1**).

We assumed that **Y‐H** should be readily prepared from **2**, which itself should be accessible from the commercially available bis(diphenylphosphino)methane (dppm). Unfortunately, initial attempts to synthesize this compound proved to be challenging, since selective oxidation of only one phosphine moiety with one equivalent of either bromine or sulfur to the respective mono‐oxidized compound, despite literature evidence, could not be achieved with sufficient selectivity.^[11]^ We therefore decided to try an alternative synthetic procedure towards **Y‐H** using methyldiphenylphosphine sulfide **1**, which was prepared according to a literature procedure in a one‐pot reaction between Ph_2_PCl and MeMgCl and subsequent treatment with elemental sulfur.^[12]^ For the synthesis of **2**, the phosphine sulfide **1** was reacted with a stoichiometric amount of *n*‐BuLi in THF followed by addition of chlorodiphenylphosphine. Removal of the solvent and work‐up gave **2** as a colourless solid in 89 % yield. The product could clearly be identified by multinuclear NMR spectroscopy, the ^31^P{^1^H} NMR spectrum showing two doublets at δ_P_=−28.4 and 40.0 ppm with a coupling constant of ^2^ *J*_PP_=77.3 Hz.^[13]^

The next step was the bromination of **2** using stoichiometric amounts of elemental bromine in DCM at low temperatures. Thereby, bromine addition must be carried out in a controlled manner to minimize side product formation. The resulting product **3** shows low solubility in DCM, thus allowing for its isolation as colorless solid in excellent yields. Spectroscopic analysis showed doublets at δ_P_=33.7 and 49.6 ppm in the ^31^P{^1^H} NMR spectrum, consistent with a transition from P^III^ to P^V^.^[14]^ Next, amination to yield **Y‐H_2_** was achieved through reaction with one equivalent of piperidine and two equivalents of trihexylamine. Unfortunately, this reaction was surprisingly unselective, always delivering the phosphine oxide as a by‐product. At 0 °C, an approx. 1 : 2 ratio of aminated to oxidized product was formed as determined by ^31^P{^1^H} NMR spectroscopy. Lowering the reaction temperature improved the selectivity, giving 75 % of the aminated compound at −90 °C. Since separation of the compounds was found to be difficult, the mixture was directly used for the subsequent deprotonation step. Fortunately, treatment of the mixture with KHMDS (HMDS=bis(trimethylsilyl)amide) in THF led to the precipitation of the deprotonated phosphine oxide, thus allowing the isolation of ylide **Y‐H** in a moderate 57 % yield on a gram scale in a single step from **3**. Ylide **Y‐H** revealed to be highly stable, showing no decomposition upon storage in a glovebox over extended periods of time. **Y‐H** is characterized by two doublets in the ^31^P{^1^H} NMR spectrum at δ_P_=33.7 and 41.7 ppm (Table 1). In the ^1^H NMR spectrum, a doublet of doublets integrating to one at *δ*_H_=1.84 ppm is indicative of successful deprotonation. Furthermore, the central carbon atom in **Y‐H** appears as a doublet of doublets at δ_C_=16.9 ppm in the ^13^C{^1^H} NMR spectrum, which is considerably shifted to higher field compared to the corresponding signal of **3**. Single crystals of **Y‐H** could be obtained from slow evaporation of a saturated diethyl ether solution (Figure 2).^[15]^ Single crystals of **Y‐H_2_** were serendipitously obtained from a reaction solution of the metallated ylide (see Supporting Information for details), thus allowing the comparison of the bond lengths and angles of the whole compound series (see Table 2).

**Figure 2:**
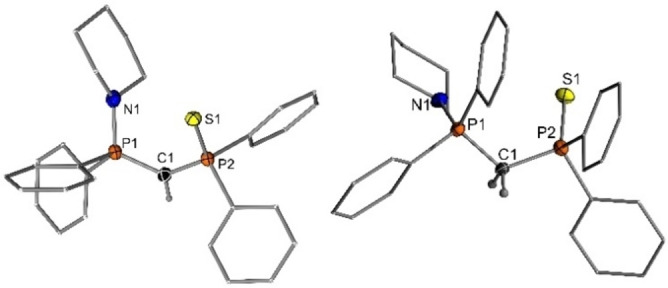
Molecular structures of **Y‐H_2_** (anion omitted) and **Y‐H**. Hydrogen atoms, except for the two H atoms bound to the central carbon atom have been omitted for clarity; thermal ellipsoids are displayed at the 50 % probability level. Selected bond lengths (Å) and angles (°): For **Y‐H_2_**: P1−C1 1.803(5), P2−C1 1.831(5), P1−N1 1.647(4), S1−P2 1.9502(16), P1−C1−P2 119.4(2). For **Y‐H**: P1−C1 1.694(2), P2−C1 1.723(2), P1−N1 1.6589(18), S1−P2 1.9743(7), P1−C1−P2 125.39(12).

With the ylide precursor in hand, we next addressed the deprotonation to the desired yldiide. Examination of different metal bases revealed that *n*‐butyllithium, sodium amide and benzyl potassium gave best results to access the different alkali metal salts, with the lithium and potassium compounds being more easily formed (Scheme 2). THF and benzene, respectively, were found to be the best solvents depending on the solubility of the reagents and products and reactions with the respective solvents. The lithium and sodium yldiide could both unambiguously be spectroscopically identified. However, they were found to be unstable in solution, showing facile re‐formation of **Y‐H** after only one hour and thus preventing isolation in pure form. This contrasts with the potassium derivative, which shows a higher degree of solution stability (the first signs of protonation appearing only after a 24 hour period), thus allowing for a full spectroscopic characterization (see Supporting Information).

**Scheme 2:**
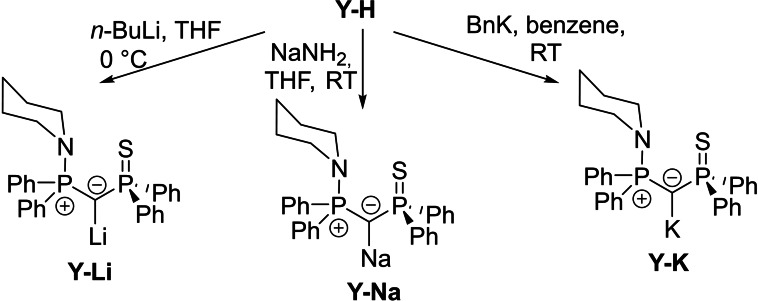
Synthesis pathway for individual alkali metal yldiides.

From the ^31^P{^1^H} NMR spectroscopic data of **Y‐M**, a few key trends can be seen for the different alkali metal salts (Figure 3, Table 1). The first is that, as group 1 is descended, an increasing shift to higher field in comparison to the ylide can be seen. This is in line with successful deprotonation and the resulting increase of electron density at the bridging carbon atom. At the same time, the ^2^ *J*_PP_ coupling constant increases, which indicates a widening of the P−C−P angle probably as a consequence of the weaker carbon metal interaction when moving down the group. In the ^13^C{^1^H} NMR spectra, a low‐field shift of the central carbon signal is observed, moving from 16.9 ppm in **Y‐H** to 29.1 ppm in **Y‐K**, as well as a concomitant decrease in coupling constants from ^1^ *J*_CP_=134.6 and 107.7 Hz to 102.0 and 81.9 Hz respectively. These trends have also previously been observed for other ylidiides.^[6]^

**Figure 3:**
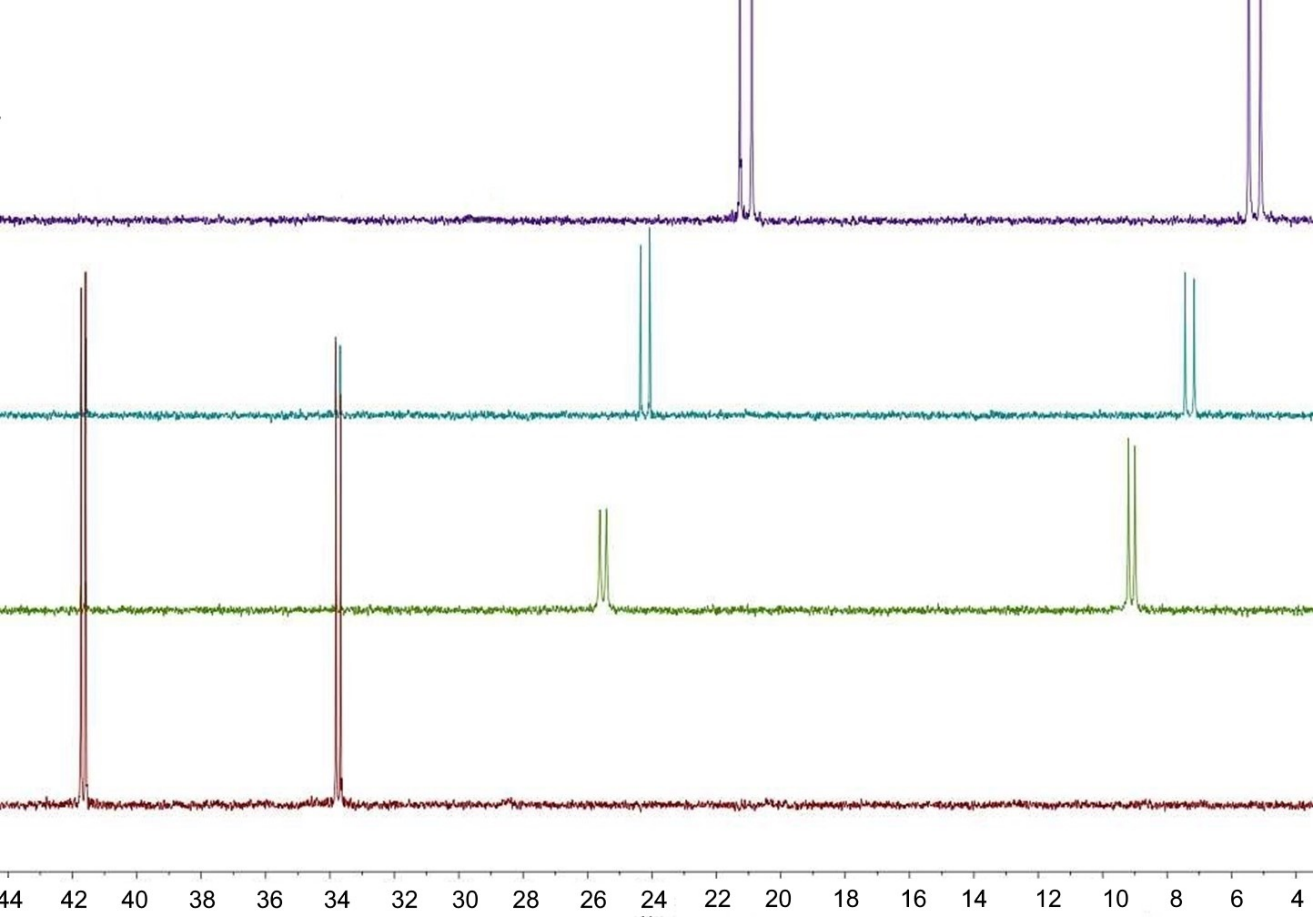
Comparison of ^31^P{^1^H} spectra of **Y‐H** (red), **LiY** (green), **NaY** (blue) and **KY** (purple) in THF‐d_8_ (shifts are given in ppm).

**Table 1:** ^31^P{^1^H} NMR shifts and coupling constants of the parent ylide **Y‐H** and the lithium, sodium and potassium yldiide in THF‐d_8_.

Species	*δ*_P_ [ppm]	^2^ *J*_PP_ [Hz]	*Δ*_C_ [ppm]	^1^ *J*_CP_ [Hz]
**Y‐H_2_**	44.7, 32.9	6.7	–	–
**Y‐H**	41.6, 33.8	23.3	16.9	134.6+107.7
**Y‐Li**	25.4, 9.2	32.5	–	–
**Y‐Na**	24.1, 7.4	48.7	–	–
**Y‐K**	20.9, 5.5	62.4	29.1	102.0+81.9

Despite the instability of the yldiides in solution, it was possible to obtain crystals of the lithium and potassium salts, both with and without addition of crown ether. The structures of all complexes are shown in Figures 4 and 5, and important bond lengths are given in Table 2. Crystallization of **Y‐Li** from THF solution gives the dimer (**Y‐Li** ⋅ THF)_2_, in which the coordination sphere of lithium is composed of one carbon center from the ylidiide, two sulfur atoms and one solvating THF molecule. The ylidic carbon atom exhibits a trigonal planar coordination environment. No contact between the piperidyl moiety and the metal is observed. The same holds true for the corresponding 12‐crown‐4 (12‐C‐4) complex [**Y_2_Li**][Li(12‐C‐4)_2_].^[16]^ To our surprise, the addition of crown ether to the THF solvated dimer did not lead to the formation of a monomeric structure as anticipated. Instead, only one lithium cation is complexed by the crown ether, while the second Li ion is coordinated by two yldiides, with the coordination sphere of the lithium center being completed by the sulfur atoms of the two ylidiides. Due to this coordination between sulfur and lithium, the Li1−C1−P2 bond angle of 91.90(13)° and Li1−C1−P1 bond angle of 132.09(15)° differ significantly from each other. The C1−Li1−C31 bond angle of 139.11(19)° shows a high discrepancy from linearity, hence the lithium center is in a distorted tetrahedral environment. The Li−C distances of 2.131(4)–2.215(4) Å in both lithium yldiides are comparable to previously studied dimeric lithium compounds.^[17]^ It is noteworthy that the Li−C distances in [**Y_2_Li**][Li(12‐C‐4)_2_] are slightly longer than those in the THF‐solvated dimer. This elongation can probably be attributed to increased steric bulk and reduced electrostatic attractions, due to one lithium cation being shared by two yldiide ligands.

**Figure 4:**
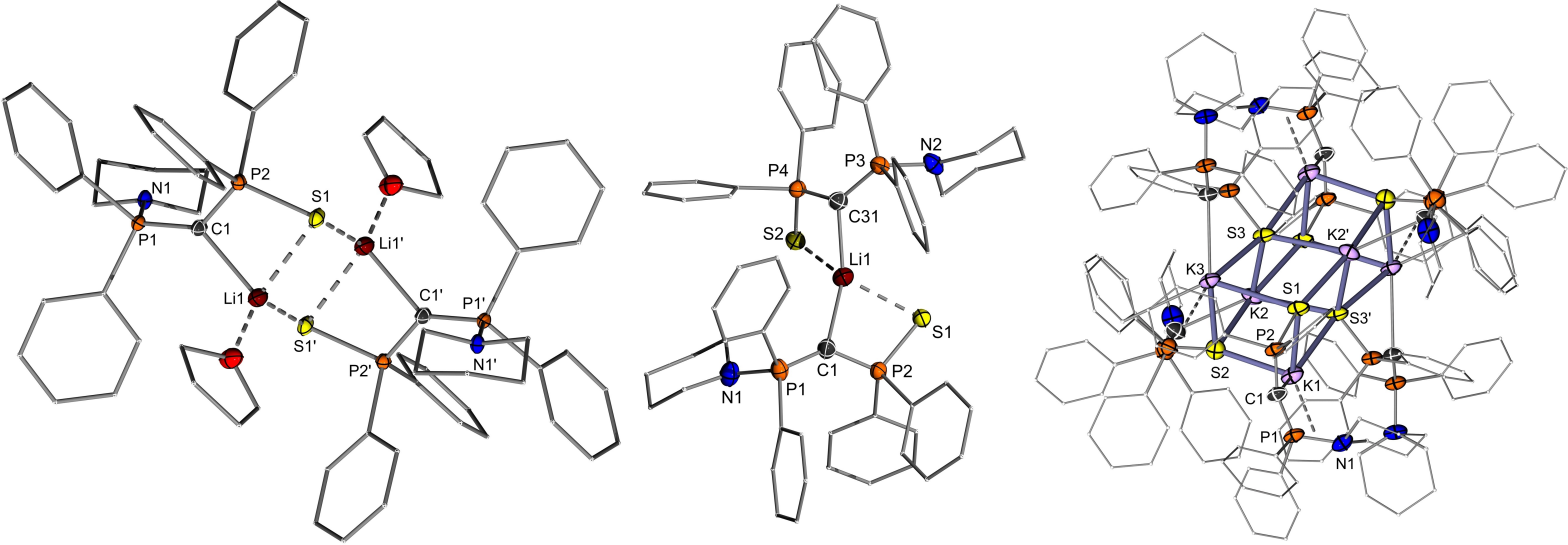
Molecular structures of (**Y‐Li** ⋅ THF)_2_, [**Y_2_Li**][Li(12‐C‐4)_2_] and (**Y‐K**)_6_. Hydrogen atoms, disorder and Li([12]C‐4)_2_^+^ have been omitted for clarity. Thermal ellipsoids are displayed at the 50 % probability level. Selected bond lengths (Å) and angles (°): (**Y‐Li** ⋅ THF)_2_: P1−C1 1.6391(17), P2−C1 1.6671(18), N1−P1 1.6976(15), S1−P2 2.0332(6), Li1−C1 2.132(4), S1−Li1 2.531(3), S1−Li1’ 2.487(3), P1−C1−P2 134.91(11); [**Y_2_Li**][Li(12‐C‐4)_2_]: P1−C1 1.639(2), P2−C1 1.658(2), N1−P1 1.725(2), S1−P2 2.0141(7), C1−Li1 2.215(4), S1−Li1 2.499(3), P1−C1−P2 135.95(13), C1−Li1−C31 139.11(19). (**Y‐K**)_6_: P1−C1 1.585(2), P2−C1 1.674(3), N1−P1 1.708(2), S1−P2 2.0337(8), C1−K1 2.872(3), S1−K1 3.1413(9), P1−C1−P2 138.93(17). Transformations used to generate symmetry‐equivalent atoms: −x, −y, −z (for (**Y‐Li** ⋅ THF)_2_) and −x, y+1/2, −z+1/2; −x, −y, −z and x, −y–1/2, z–1/2 for (**Y‐K**)_6_.

**Figure 5:**
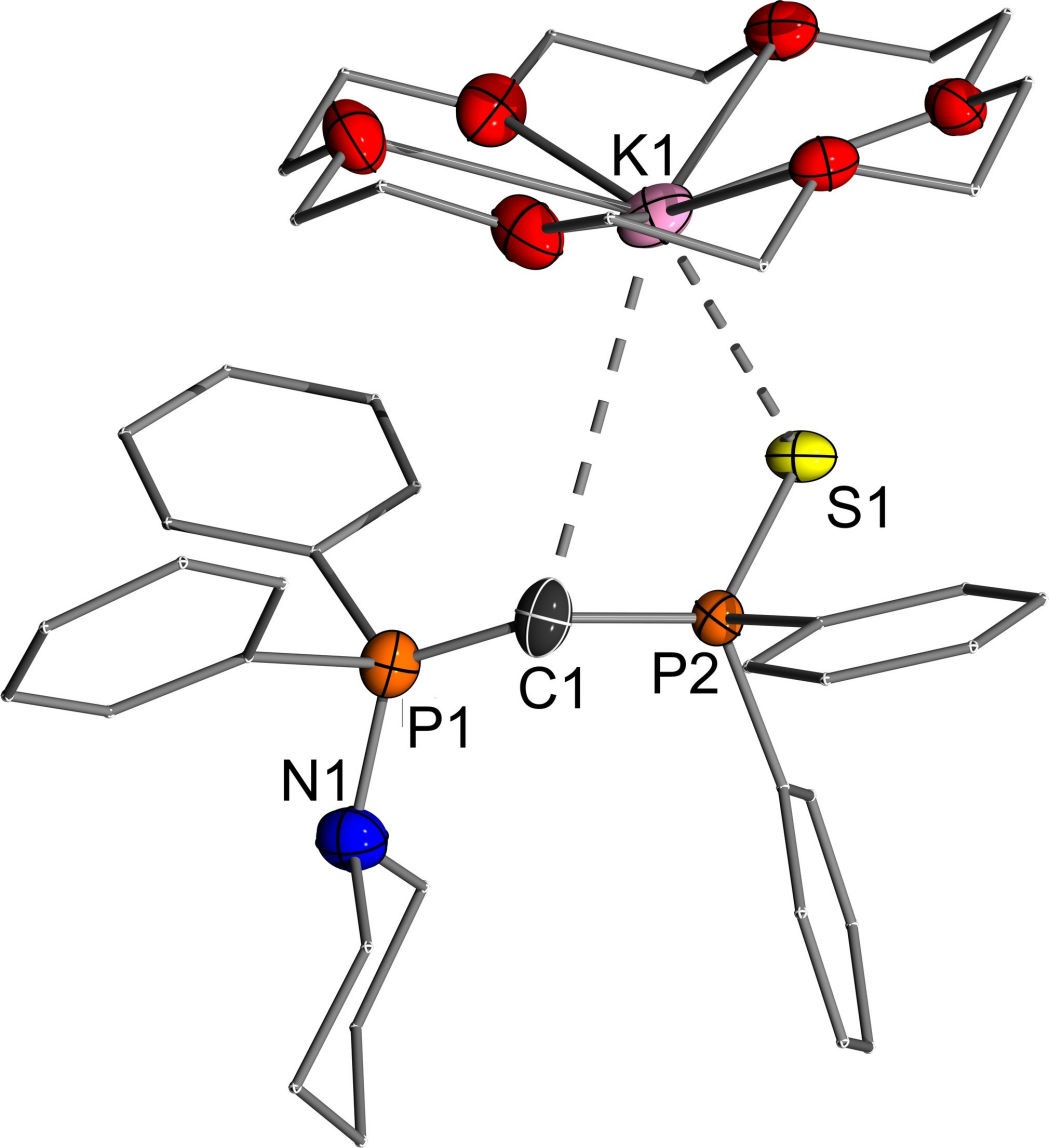
Molecular structure of [**Y‐K** ⋅ (18‐C‐6)]. Hydrogen atoms and disorder omitted for clarity. Ellipsoids are displayed at the 50 % probability level. Selected bond lengths (Å) and angles (°): P1−C1 1.609(3), P2−C1 1.660(2), K1−C1 3.857(4), N1−P1 1.729(2), S1−P2 2.0192(8), S1−K1 3.1573(12), P1−C1−P2 139.59(16).

**Table 2:** Selected structural parameters of ylide **Y‐H** and the lithium and potassium yldiides.

Species	P1−C1 P2−C1 [Å]	P−S [Å]	P1−N [Å]	P1−C_Ph_ [Å]	P−C−P [°]
**Y‐H**	1.694(2) 1.723(2)	1.997(1)	1.659(2)	1.812(2) 1.803(2)	125.4(1)
(**YLi** ⋅ THF)_2_	1.638(1) 1.668(1)	2.034(1)	1.696(1)	1.825(2) 1.822(2)	135.0(1)
[**Y_2_Li**]^−^	1.639(2) 1.657(2)	2.014(7)	1.725(2)	1.802(2)1.790(8)	136.0(1)
(**Y‐K**)_6_	1.626(2) 1.658(2),	2.034(1)	1.707(2)	1.827(3) 1.826(3)	136.6(2)
**KY**	1.610(5) 1.661(4)	2.019(1)	1.733(4)	1.845(3) 1.830(3)	139.5(3)

In addition to the structures of the lithium complexes, we were also able to obtain two structures of the potassium yldiide: the solvent‐free aggregate (**Y‐K**)_6_ and the 18‐crown‐6‐coordinated monomer [**Y‐K**(18‐C‐6)]. (**Y‐K**)_6_ crystalizes as an *C_i_*‐symmetric aggregate of six yldiide units. The central core is comprised of two face‐linked distorted K_4_S_4_ cubes, with the corners of the cubes alternately occupied by sulfur and potassium atoms. Each potassium atom features an additional contact with the central carbon atom of the ylidiide. Furthermore, the potassium ions on the corners of the (KS)_6_ core additionally interact with one aryl group of the thiophosphoryl group, thus resulting in a strongly distorted trigonal bipyramidal coordination sphere. The C−K distances range from 2.872(3) to 2.990(2) Å, which is relatively short compared to literature distances from comparable compounds, suggestive of a relatively strong bond.^[18]^

In contrast to the lithium yldiide (**Y‐Li** ⋅ THF)_2_, addition of stoichiometric amounts of 18‐crown‐6 (18‐C‐6) to (**Y‐K**)_6_ allowed the formation of a monomeric structure (Figure 5). The potassium center is coordinated to both the sulfur atom and the central ylidic carbon atom, with its coordination sphere completed by crown ether oxygen atoms. It is worth noting that the interaction between potassium and the central carbon atom is the weakest of all the yldiide structures studied here, with a distance of 3.857(4) Å compared to the sum of the van der Waals radii of 4.45 Å.^[19]^ This presumably leads to a higher charge concentration at C1 and the shortest P1−C1 bond (1.609(3) Å) within the whole series of metallated ylides (Table 2).

A comparison of the structural features of the lithium and potassium yldiides with the ylide and phosphonium salt precursor (Table 2) reveals that – as expected – the yldiides exhibit shorter P−C distances than **Y‐H** and particularly **Y‐H_2_** due to increased electrostatic attractions within the P−C−P linkage. At the same time, the bonds between the phosphorus atoms and their substituents (P−N, P−S and P−C_Ph_) elongate because of negative hyperconjugation effects. Interestingly, this elongation is more pronounced for the P−N bond than for the P−C_Ph_ bond in the phosphonium group, suggesting that the amino group – although not coordinating to the metal – significantly contributes to the stability of the yldiide by electronic stabilization of the charge at the ylidic carbon atom. Furthermore, the P−C−P bond angle increases in both the lithium and the potassium yldiides when compared to the parent **Y‐H** and **Y‐H_2_** compound, which implies an increasing s‐character in the phosphorus‐carbon bond. The larger angles are found for the potassium compounds, which is well in line with the increasing ^2^ *J*_PP_ coupling constant when descending the group (Figure 3).

To gain insight into the bonding situation and charge distribution in the metallated ylides, natural bond orbital analysis (NBO)^[20]^ studies of the dimeric THF complex of **Y‐Li** and monomeric **Y‐K** with crown ether compared with the ylide precursor **Y‐H** were performed. The calculations reflect a moderate increase of electron density at the central carbon atom C1 upon metallation, with an increase of the negative charge from *q*_C_=−1.367 e in **Y‐H** to *q*_C_=−1.624 e and −1.553 e in the lithium and potassium yldiide, respectively. A further increase of electron density is observed for the sulfur atom (Δ*q_S_* ≈−0.15 e), whereas the changes in charges at all other atoms are only small (Δ*q*<0.1). The increase of negative charge at the sulfur atom is also accompanied by a higher occupation of the antibonding σ*(P2−S) orbital, which is in line with substantial negative hyperconjugation effects, also expressed by a weakening of the P−S bond, that is, a reduced Wiberg bond index (WBI). This supports the strong anion‐stabilizing ability of the thiophosphinoyl moiety, which decisively contributes to the overall stability of the yldiide. Although the WBI of the P1−N and P1−C_Ph_ bonds in the yldiides barely change compared with those of **Y‐H**, the negative hyperconjucation into the phosphonium group becomes evident from the changes in the occupancy of the corresponding σ* orbitals.^[21]^ As can be seen from Table 3, the change in the occupancy is more pronounced for the P−N bond compared with the P−C_Ph_ bond, suggesting that the piperidine moiety accepts more electron density and hence contributes to the stability of the yldiide. This is well in line with the crystallographic data (see above). It is also noteworthy that the overall changes are more pronounced for the potassium compound than for the lithium compound which corroborates the more covalent character of organolithium compounds.^[22]^

**Table 3:** Results of the NBO analyses of **Y‐H**, (**YLi** ⋅ THF)_2_ and [**Y‐K**(18‐C‐6)] at the PW6B95‐D3/def2TZVP level of theory.^[23]^

	**Y‐H**	(**YLi** ⋅ THF)_2_	[**Y‐K**(18‐C‐6)]
WBI (P2=S)	1.306	1.044	1.122
WBI (P1−C_Ph_)^[a]^	0.865	0.914	0.813
q(C1)	−1.367	−1.624	−1.553
q(S)	−0.650	−0.796	−0.793
q(N)	−0.874	−0.869	−0.876
occ σ*(P2−S)	0.030	0.085 (Δ=0.055)^[b]^	0.116 (Δ=0.086^b]^
occ σ*(P1−N1)	0.145	0.186 (Δ=0.042)^[b]^	0.206 (Δ=0.061)^[b]^
occ σ*(P1−C_Ph_)^[a]^	0.101	0.129 (Δ=0.028)^[b]^	0.144 (Δ=0.043)^[b]^

[a] average values; [b] change in occupation relative to **Y‐H**.

In conclusion, we successfully isolated and characterized a series of stable thiophosphinoyl‐ and amino‐substituted metallated ylides. The lithium and potassium salts formed different structures in the solid state (monomer, dimer and hexamer), including a monomeric potassium crown ether complex. The structures reflect the increasing electrostatic attractions within the P−C−P linkage upon metallation, expressed by the shortening of the P−C bonds. Negative hyperconjugation effects mirrored by the lengthening of the bonds to the β‐substituents contribute to the stability of the compound and demonstrate the strong anion‐stabilizing ability of the P=S moiety. Interestingly, the lengthening of the bonds in the phosphonium group was more pronounced for the P−N than the P−C_Ph_ bond, demonstrating its importance for stabilizing the metallated ylide by negative hyperconjugation. This stabilizing effect was also confirmed by NBO analyses, showing a considerable increase in the occupancy of the σ*(P−N) orbital. Current studies are focusing on the application of the metallated ylides **Y‐M** in main group chemistry.

## Conflict of interest

The authors declare no conflict of interest.

## Supporting information

As a service to our authors and readers, this journal provides supporting information supplied by the authors. Such materials are peer reviewed and may be re‐organized for online delivery, but are not copy‐edited or typeset. Technical support issues arising from supporting information (other than missing files) should be addressed to the authors.

Supporting Information

## References

[1] For review articles, see:

[1a] M. Taillefer , H. J. Cristau , in New Aspects in Phosphorus Chemistry III. Topics in Current Chemistry, Springer, 2003;

[1b] G. D. Bisag , S. Ruggieri , M. Fochi , L. Bernardi , Org. Biomol. Chem. 2020, 18, 8793; 33084717 10.1039/d0ob01822h

[1c] R. Oost , J. D. Neuhaus , J. Merad , N. Maulide , in Modern Ylide Chemistry: Applications in Ligand Design, Organic and Catalytic Transformations, Structure and Bonding, Springer, 2018, 2003.

[2] For reviews, see:

[2a] L. Scharf , V. H. Gessner , Inorg. Chem. 2017, 56, 8599; 28240888 10.1021/acs.inorgchem.7b00099 PMC5549244

[2b] V. H. Gessner , in Modern Ylide Chemistry: Applications in Ligand Design, Organic and Catalytic Transformations, Structure and Bonding, Springer, 2018;

[2c] H.-J. Cristau , Chem. Rev. 1994, 94, 1299.

[3a] E. J. Corey , J. Kang , J. Am. Chem. Soc. 1982, 104, 4724;

[3b] E. J. Corey , J. Kang , K. Kyler , Tetrahedron Lett. 1986, 26, 555.

[4a] M. Schlosser , H. BaTuong , J. Respondek , B. Schaub , Chimia 1983, 37, 10;

[4b] B. Schaub , T. Jenny , M. Schlosser , Tetrahedron Lett. 1984, 25, 4097;

[4c] B. Schaub , M. Schlosser , Tetrahedron Lett. 1985, 26, 1623.

[5a] H. J. Bestmann , M. Schmidt , Angew. Chem. Int. Ed. 1987, 26, 79;

[5b] H. J. Bestmann , M. Schmidt , Tetrahedron Lett. 1987, 28, 2111;

[5c] C. Schwarz , L. T. Scharf , T. Scherpf , J. Weismann , V. H. Gessner , Chem. Eur. J. 2019, 25, 2793. 30556625 10.1002/chem.201805421 PMC6519153

[6a] S. Goumri-Magnet , H. Gornitzka , A. Baceiredo , G. Bertrand , Angew. Chem. Int. Ed. 1999, 38, 678; 10.1002/(SICI)1521-3773(19990301)38:5<678::AID-ANIE678>3.0.CO;2-L 29711539

[6b] T. Baumgartner , B. Schinkels , D. Gudat , M. Nieger , E. Niecke , J. Am. Chem. Soc. 1997, 119, 12410;

[6c] A. Garduno-Alvia , R. Lenk , Y. Escudié , M. L. González , L. Bousquet , N. Saffon-Merceron , C. A. Toledano , X. Bagan , V. Branchadell , E. Maerten , A. Baceiredo , Eur. J. Inorg. Chem. 2017, 2017, 3494;

[6d] T. Scherpf , R. Wirth , K.-S. Feichtner , S. Molitor , V. H. Gessner , Angew. Chem. Int. Ed. 2015, 54, 8542; 10.1002/anie.201501818 26094883

[6e] H. Darmandeh , T. Scherpf , K.-S. Feichtner , C. Schwarz , V. H. Gessner , Z. Anorg. Allg. Chem. 2020, 646, 835. 32742041 10.1002/zaac.201900333 PMC7386922

[7] C. Schwarz , T. Scherpf , I. Rodstein , J. Weismann , K.-S. Feichtner , V. H. Gessner , Chem. Open. 2019, 8, 621. 10.1002/open.201900094 PMC6519319 31123665

[8a] T. Scherpf , K.-S. Feichtner , S. Molitor , V. H. Gessner , Angew. Chem. Int. Ed. 2017, 56, 3275; 10.1002/anie.201611677 PMC5363341 28185370

[8b] C. Mohapatra , L. Scharf , T. Scherpf , B. Mallick , K.-S. Feichtner , C. Schwarz , V. H. Gessner , Angew. Chem. Int. Ed. 2019, 58, 7459; 10.1002/anie.201902831 PMC6563488 30901140

[8c] C. Mohapatra , H. Darmandeh , H. Steinert , B. Mallick , K.-S. Feichtner , V. H. Gessner , Chem. Eur. J. 2020, 26, 15145. 32954596 10.1002/chem.202004242 PMC7756224

[9a] T. Scherpf , C. Schwarz , L. T. Scharf , J. -A Zur , A. Helbig , V. H. Gessner , Angew. Chem. Int. Ed. 2018, 57, 12859; 10.1002/anie.201805372 PMC6174943 29862622

[9b] P. Weber , T. Scherpf , I. Rodstein , D. Lichte , L. T. Scharf , L. J. Gooßen , V. H. Gessner , Angew. Chem. Int. Ed. 2019, 58, 3203; 10.1002/anie.201810696 30451339

[9c] X.-Q. Hu , D. Lichte , I. Rodstein , P. Weber , A.-K. Seitz , T. Scherpf , V. H. Gessner , L. J. Gooßen , Org. Lett. 2019, 21, 7558; 31469570 10.1021/acs.orglett.9b02830

[9d] C. Schwarz , J. Handelmann , D. M. Baier , A. Ouissa , V. H. Gessner , Catal. Sci. Technol. 2019, 9, 6808;

[9e] L. T. Scharf , I. Rodstein , M. Schmidt , T. Scherpf , V. H. Gessner , ACS Catal. 2020, 10, 999; 32030314 10.1021/acscatal.9b04666 PMC6996648

[9f] J. Tappen , I. Rodstein , K. McGuire , A. Großjohann , J. Löffler , T. Scherpf , V. H. Gessner , Chem. Eur. J. 2020, 26, 4281; 31971642 10.1002/chem.201905535 PMC7186839

[9g] T. Scherpf , H. Steinert , A. Großjohann , K. Dilchert , J. Tappen , I. Rodstein , V. H. Gessner , Angew. Chem. Int. Ed. 2020, 59, 20596; 10.1002/anie.202008866 PMC7692947 32725943

[9h] I. Rodstein , D. S. Prendes , L. Wickert , M. Paaßen , V. H. Gessner , J. Org. Chem. 2020, 85, 14674; 32907331 10.1021/acs.joc.0c01771 PMC7684579

[9i] Z. Hu , X.-J. Wei , J. Handelmann , A.-K. Seitz , I. Rodstein , V. H. Gessner , L. J. Gooßen , Angew. Chem. Int. Ed. 2021, 60, 6778. 10.1002/anie.202016048 PMC7986804 33427381

[10a] A. Sarbajna , V. S. V. S. N. Swamy , V. H. Gessner , Chem. Sci. 2021, 12, 2016; 10.1039/d0sc03278f PMC8179322 34163963

[10b] R. Zurawinski , C. Lepetit , Y. Canac , M. Mikolajczyk , R. Chauvin , Inorg. Chem. 2009, 48, 2147. 19235974 10.1021/ic802104f

[11] L. Boubekeur , L. Ricard , N. Mézailles , P. Le Floch , Organometallics 2005, 24, 1065.

[12a] R. A. Zingaro , R. E. McGlothin , J. Chem. Eng. Data 1963, 8, 226;

[12b] V. H. Gessner , Organometallics 2011, 30, 4228.

[13] J. Weismann , V. H. Gessner , Chem. Commun. 2015, 51, 14909. 10.1039/c5cc05201g 26304998

[14a] M. Demange , L. Boubekeur , A. Auffrant , N. Mézailles , L. Ricard , X. Le Goff , P. Le Floch , New J. Chem. 2006, 30, 1745;

[14b] L. Boubekeur , L. Ricard , N. Mézailles , P. Le Floch , Organometallics 2005, 24, 1065.

[15] Deposition Number(s) 2100502 (for (**Y-K**)_6_), 2100503 (for **3**), 2100504 (for **Y-H**), 2100505 (for **Y-H** _ **2** _), 2100506 (for [**Y-K**(18-C-6)]), 2100507 (for [**Y** _ **2** _ **Li**][Li(12-C-4)_2_]) and 2100508 (for (**YLi** ⋅ THF)_2_) contain the supplementary crystallographic data for this paper. These data are provided free of charge by the joint Cambridge Crystallographic Data Centre and Fachinformationszentrum Karlsruhe Access Structures service.

[16] For other examples of lithium lithiate structures, see:

[16a] A.-C. Pöppler , M. Granitzka , R. Herbst-Irmer , Y.-S. Chen , B. B. Iversen , M. John , R. A. Mata , D. Stalke , Angew. Chem. Int. Ed. 2014, 53, 13282; 10.1002/anie.201406320 25284593

[16b] J. A. Greer , V. L. Blair , C. D. Thompson , P. C. Andrews , Dalton Trans. 2016, 45, 10887; 27327036 10.1039/c6dt01630h

[16c] H. Gornitzka , D. Stalke , Angew. Chem. Int. Ed. 1994, 33, 693;

[16d] S. Harder , M. H. Prosenc , Angew. Chem. Int. Ed. 1994, 33, 1744.

[17a] T. Cantat , L. Ricard , P. Le Floch , N. Mézailles , Organometallics 2006, 25, 4965;

[17b] C. P. Sindlinger , A. Stasch , Dalton Trans. 2014, 43, 14334; 24898367 10.1039/c4dt01185f

[17c] A. Harrison-Marchand , F. Morgin , Chem. Rev. 2013, 113, 7470. 23952819 10.1021/cr300295w

[18a] Y.-F. Yang , C. Foo , R. Ganguly , Y. Li , C.-W. So , Organometallics 2012, 31, 6538;

[18b] L. Orzechowski , G. Jansen , S. Harder , Angew. Chem. Int. Ed. 2009, 48, 3825. 10.1002/anie.200900830 19373819

[19a] M. Mantina , A. C. Chamberlin , R. Valero , C. J. Cramer , G. D. Truhlar , J. Phys. Chem. A 2009, 113, 5806. 19382751 10.1021/jp8111556 PMC3658832

[20] E. D. Glendening , J. K. Badenhoop , A. E. Reed , J. E. Carpenter , J. A. Bohmann , C. M. Morales , P. Karafiloglou , C. R. Landis , F. Weinhold , NBO7, Theoretical Chemistry Institute, University of Wisconsin, Madison, 2018.

[21] Further literature on negative hyperconjugation effects in alkali metal chemistry:

[21a] M. Köhler , A. Koch , H. Görls , M. Westerhausen , Organometallics 2016, 35, 242;

[21b] M. Karni , C. F. Bernasconi , Z. Rappoport , J. Org. Chem. 2008, 73, 2980; 18376875 10.1021/jo7017476

[21c] P. v R Schleyer , T. Clark , A. J. Kos , G. W. Spitznagel , C. Rohde , D. Arad , K. N. Houk , N. G. Rondan , J. Am. Chem. Soc. 1984, 106, 6467;

[21d] W. Scherer , P. Sirsch , D. Shorokhov , G. S. McGrady , S. A. Mason , M. G. Gardiner , Chem. Eur. J. 2002, 8, 2324. 12012416 10.1002/1521-3765(20020517)8:10<2324::AID-CHEM2324>3.0.CO;2-X

[22a] T. X. Gentner , R. E. Mulvey , Angew. Chem. Int. Ed. 2021, 60, 9247; 10.1002/anie.202010963 PMC8247348 33017511

[22b] C. Lambert , M. Kaupp , P. v Ragué Schleyer , Organometallics 1993, 12, 853;

[22c] S. Molitor , V. H. Gessner , Angew. Chem. Int. Ed. 2016, 55, 7712. 10.1002/anie.201601356 27100278

[23a] L. Goerigk , A. Hansen , C. Bauer , S. Ehrlich , A. Najini , S. Grimme , Phys. Chem. Chem. Phys. 2017. 19, 32184; 29110012 10.1039/c7cp04913g

[23b] L. Goerigk , S. Grimme , J. Chem. Theory Comput. 2011, 7, 291; 26596152 10.1021/ct100466k

[23c] S. Grimme , J. Antony , S. Ehrlich , H. Krieg , J. Chem. Phys. 2010, 132, 154104; 20423165 10.1063/1.3382344

[23d] F. Weigand , Phys. Chem. Chem. Phys. 2006, 8, 1057.16633586

