# Supplementary material for: Synthesis, Crystal and Electronic Structures of a Thiophosphinoyl‐ and Amino‐Substituted Metallated Ylide
Source: ChemistryOpen. 2021 Sep 27;10(11):1089–94. doi: 10.1002/open.202100187 (PMC8562316; doi:10.1002/open.202100187)
Supplement: Supplementary file 1 — Supporting Information [file OPEN-10-1089-s001.pdf]

ChemistryOpen

Supporting Information

Synthesis, Crystal and Electronic Structures of a Thiophosphinoyl- and Amino-Substituted Metallated Ylide

Mike Jörges, Alexander Kroll, Leif Kelling, Richard Gauld, Bert Mallick, Stefan M. Huber, and Viktoria H. Gessner*

Table of Contents

1. Experimental Details 2
1.1. General Experimental Information 2
1.2 Synthesis of compound 1 2
1.3 Synthesis of compound 2 3
1.4 Synthesis of compound 3 3
1.5 Synthesis of Ylide Y-H 4
1.6 Synthesis of metalated Ylide Y-Li 5
1.7 Synthesis of metalated Ylide Y-Na 6
1.8 Synthesis of metalated Ylide Y-K 6
2. NMR spectra of the isolated compounds and reaction mixtures 8
3. Crystal structure determination. 22
3.1 General information 22
3.2 Crystal Structure Determination of 3 26
3.3 Crystal Structure Determination of Y-H_2 31
3.4 Crystal Structure Determination of Y-H 35
3.5 Crystal Structure Determination of $(Y\text{-}Li\cdot THF)_2$ 38
3.6 Crystal Structure Determination of $[Y_2Li][Li(12\text{-}C\text{-}4)_2]$ 42
3.7 Crystal Structure Determination $(Y\text{-}K)_6$ 49
3.8 Crystal Structure Determination [Y-K·(18-C-6)] 56
4. Computational Details 62
5. References 69

1. Experimental Details

1.1. General Experimental Information

All experiments were carried out under a dry, oxygen-free argon atmosphere using standard Schlenk techniques. Involved solvents were dried using an MBraun SPS-800 (THF, DCM, toluene, acetonitrile, diethylether and pentane) or dried in accordance with standard procedures. Deuterated solvents were stored over molecular sieves in an argon-filled glovebox. BnK was prepared according to published procedures.[1] All other reagents were purchased from Sigma-Aldrich, ABCR, Rockwood Lithium or Acros Organics or donated by Umicore and used without further purification.

NMR spectra were recorded on Avance-400 spectrometers at 25 °C if not stated otherwise. All values of the chemical shift are in ppm regarding the δ-scale. All spin-spin coupling constants (*J*) are printed in Hertz (Hz). To display multiplicities and signal forms correctly the following abbreviations were used: s = singlet, d = doublet, t = triplet m = multiplet, dd = doublet of doublet, br = broad signal. Signal assignment was supported by, HSQC (^{1}H / ^{13}C), HMBC (^{1}H / ^{13}C, ^{1}H / ^{31}P) correlation experiments.

Elemental analyses were performed on an Elementar vario MICRO-cube elemental analyzer.

For details about the single-crystal Xray diffraction analyses, see chapter 3.

1.2 Synthesis of compound 1

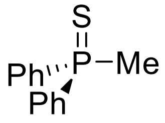

12.3 g (55.6 mmol) chlorodiphenylphosphine were dissolved in 115 mL diethylether and cooled to –78 °C. At that temperature 19 mL methylmagnesium bromide (3M in THF, 55.6 mmol) were added and warmed to ambient temperature. After stirring for 2 h at rt 1.78 g (55.6 mmol) sulfur were added and further stirred for 18 h. 90 mL water and 22 mL 2M hydrochloric acid were added, the mixture extracted with diethyl ether (3 x 150 mL) and the combined organic layers dried over sodium sulfate. Removal of the solvent afforded yellow oil which was purified by Kugelrohr distillation (bp: 135 °C, 1·10-3 mbar) yielding the product as colorless oil (10.7 g, 46.15 mmol; 83 %), which solidifies upon cooling. Analytical data match published data.[2]

1.3 Synthesis of compound 2

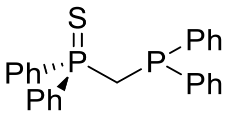

27.0 g (116 mmol) methyldiphenylphosphine sulfide were dissolved in 300 mL THF and cooled to –78 °C. At this temperature 83 mL (128 mmol) *n*BuLi (1.53 M in hexane) were added slowly and the resultant yellow mixture stirred for 3 h at low temperature. In a second Schlenk flask 27.1 mL (151 mmol) chlorodiphenylphosphine were dissolved in 120 mL THF and cooled to –78 °C. The lithiated phosphane sulfide was added dropwise via cannula transfer, stirred for 2 h at low temperature and slowly warmed to ambient temperature. Removal of the solvent afforded yellow oil which was solved in 400 ml DCM and stirred for 1 h. After filtration, evaporation of the solvent and washing with pentane (3 x 100 mL), the product was afforded as colorless solid (43.1 g, 103 mmol; 89 %). Analytical data match published data.[3]

1.4 Synthesis of compound 3

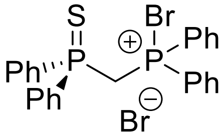

1.00 g (2.43 mmol) bis(diphenylphosphine)-methyl sulfide were dissolved in 20 mL DCM and cooled to 0 °C. At this temperature 124.5 µL (2.43 mmol) bromine were added, stirred for 0.5 h at low temperature and slowly warmed to ambient temperature. Removal of the solvent afforded a light yellow solid which was washed with THF (2 x 30 mL) and pentane (1 x 30 mL). The product was afforded as colorless solid (1.27 g, 2.20 mmol; 91 %). Single crystals suitable for X-ray diffraction analysis were grown by slow concentration of a solution of the compound in DCM and pentane. ^{1}H-NMR (400.3 MHz, CD_2Cl_2): δ = 6.05 (dd, $^{2}J_{HP}$ =14.5 Hz +11.8 Hz; P*CH*$_2$P), 7.34-7.42 (m; BrPC$H_{Ph,ortha}$), 7.42-7.45 (m; BrPC$H_{Ph,para}$), 7.45-7.54 (m; SPC$H_{Ph,ortho}$), 7.64-7.72 (m; SPC$H_{Ph,para}$), 7.99-8.10 (m; SPC$H_{Ph,meta}$), 8.22-8.33 (m; BrPC$H_{Ph,metha}$) ppm. ^{31}P{^{1}H}-NMR (162.1 MHz, CD_2Cl_2): δ = 33.7 (d, $^{2}J_{PP}$ = 10.5 Hz; S*P*Ph$_2$), 49.6 (d, $^{2}J_{PP}$ = 10.5 Hz; Br*P*Ph$_2$) ppm. ^{13}C{^{1}H}-NMR (100.7 MHz, CD_2Cl_2): δ = 32.3 (dd, $^{1}J_{CP}$ = 43.8 Hz + 40.2 Hz; P*C*H$_2$P), 120 (dd, $^{1,3}J_{CP}$ = 85.4 Hz + 1.4 Hz; SP$C_{Ph,ipso}$), 129.2 (d, $^{2}J_{CP}$ = 13.5 Hz; BrPC$H_{Ph,ortha}$), 130.1 (d, $^{3}J_{CP}$ = 15.1 Hz; SP*C*H$_{Ph,ortho}$), 131,6 (dd, $^{1,3}J_{CP}$ = 85.9 Hz + 3.6 Hz; BrP$C_{Ph,ipso}$), 132.3 (d, $^{2}J_{CP}$ = 11.9 Hz; BrP*C*H$_{Ph,metha}$), 132.7 (d, $^{4}J_{CP}$ = 3.3 Hz; BrP*C*H$_{Ph,para}$), 134.5 (d, $^{3}J_{CP}$ = 13.4 Hz; BrP*C*H$_{Ph,metha}$), 136.6 (d, $^{4}J_{CP}$ = 3.6 Hz; SP*C*H$_{Ph,para}$) ppm. Anal. Calcd for $C_{25}H_{22}P_2SBr_2$: C, 52.11; H, 3.85; S, 5.56. Found: C, 52.26; H, 4.126; S, 5.585.

1.5 Synthesis of Ylide Y-H

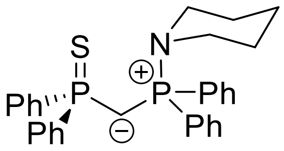

28.7 g (49.7 mmol) bromo bis(diphenylphosphine)-methyl sulfide were dissolved in 400 mL DCM and cooled to −90 °C. In a second Schlenk flask 4.9 mL (49.7 mmol) piperidine and 33.8 mL (99.4 mmol) trihexylamine were dissolved in 15 mL DCM and cooled to –90 °C. The piperidine solution was added dropwise via cannula transfer, stirred for 2 h at low temperature and slowly warmed to ambient temperature. Removal of the solvent afforded yellow oil, which was solved in 350 ml THF and cooled to −78 °C. In a second Schlenk flask 19.8 g (99.4 mmol) KHMDS were dissolved in 300 mL THF and cooled to –78 °C. The KHMDS solution was added dropwise via cannula transfer and slowly warmed to ambient temperature after completion of the addition and was stirred for 3 h. After filtration and evaporation of the solvent again a yellow oil could be obtained. 3.6 g (150 mmol) NaH and 800 mL cyclohexane were added and stirred overnight. After another filtration and evaporation of the solvent, a yellow wet solid was obtained. After washing with hexane (2 x 40 mL) and pentane (2 x 40 mL) the product was afforded as colorless solid (14.1 g, 28.2 mmol; 57 %). Single crystals suitable for X-ray diffraction analyses were grown by slow evaporation of a saturated solution of Y-H in diethyl ether. ^{1}H-NMR (400.3 MHz, THF-d_8): δ = 1.54-1.61 (br; $CH_{2,\mathrm{Pip},2,3,4}$), 1.84 (dd, $^{2}J_{HP}$ =3.78 Hz + 1.13 Hz; PC*H*P), 3.05-3.14 (br; $CH_{2,\mathrm{Pip},1,5}$), 7.20-7.26 (m; SPC$H_{\mathrm{Ph},ortho,para}$), 7.30-7.37 (m; NPC$H_{\mathrm{Ph},ortho}$), 7.38-7.44 (m; NPC$H_{\mathrm{Ph},para}$) 7.79-7.86 (m; SPC$H_{\mathrm{Ph},meta}$), 7.87-7.96 (m; NPC$H_{\mathrm{Ph},meta}$) ppm. ^{31}P{^{1}H}-NMR (162.1 MHz, THF-d_8): δ = 33.7 (d, $^{2}J_{PP}$ = 23.5 Hz; S*P*Ph_2), 41.7 (d, $^{2}J_{PP}$ = 23.5 Hz; N*P*Ph_2) ppm. ^{13}C{^{1}H}-NMR (100.7 MHz, THF-d_8): δ = 16.9 (dd, $^{1}J_{CP}$ = 134.6 Hz +107.7 Hz; P*C*HP), 25.6-26.1 (m; N*C*$H_{\mathrm{Pip},3}$), 27.2 (d, $^{3}J_{CP}$ = 6.69 Hz; N*C*$H_{\mathrm{Pip},2,4}$), 47.9 (d, $^{2}J_{CP}$ = 1.98 Hz; N*C*$H_{\mathrm{Pip},1,5}$), 128 (d, $^{2}J_{CP}$ = 12.0 Hz; SP*C*$H_{\mathrm{Ph},ortho}$), 129 (d, $^{2}J_{CP}$ = 12.3 Hz; NP*C*$H_{\mathrm{Ph},ortho}$), 130 (d, $^{4}J_{CP}$ = 2.84 Hz; SP*C*$H_{\mathrm{Ph},para}$), 132 (d, $^{3}J_{CP}$ = 10.6 Hz; NP*C*$H_{\mathrm{Ph},meta}$), 132 (d, $^{4}J_{CP}$ = 2.84 Hz; NP*C*$H_{\mathrm{Ph},para}$), 133 (dd, $^{1,3}J_{CP}$ = 110 Hz + 4.81Hz; SP*C*$H_{\mathrm{Ph},ipso}$), 133 (d, $^{3}J_{CP}$ = 9.75 Hz; SP*C*$H_{\mathrm{Ph},meta}$), 143 (dd, $^{1,3}J_{CP}$ = 85.4 Hz + 4.21Hz; NP*C*$H_{\mathrm{Ph},ipso}$) ppm. Anal. Calcd for $C_{30}H_{31}NP_2S$: C, 72.12; H, 6.25; N, 2.80. Found: C, 71.89; H, 6.10; N, 3.06.

1.6 Synthesis of metalated Ylide Y-Li

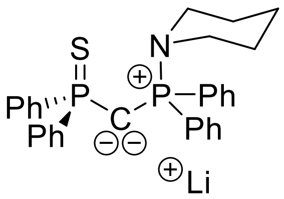

30 mg (0.06 mmol) Y-H were dissolved in 5 mL THF and cooled to 0 °C. At this temperature, 39 µL (0.06 mmol) *n*BuLi (1.53 M in hexane) were added resulting in a color change to dark yellow. Single crystals suitable for X-ray diffraction analyses were grown by two different ways: First, by slow evaporation of a saturated solution of **Y-Li** in THF at –30 °C and second by adding a small amount of 12-crown-4 ether to a saturated solution of **Y-Li** in diethyl ether. Isolation of the THF and crown ether complexes in larger quantities failed, due to the fast re-protonation to the ylide. This also holds true for the crown ether complex. Attempts to obtain further structural information by recording NMR spectra of **Y-Li** in C_6D_6 failed. Probably due to the formation of oligomeric structures, only very broad signals could be observed. Upon addition of crown ether to a C_6D_6 solution of **Y-Li** well resolved Signals were obtained in the ^{31}P NMR. Longer measurement times again resulted in re-protonation.

Y-Li THF complex:

^{1}H-NMR (400.3 MHz, THF-d_8): δ = 1.23-1.41 (br; C$H_{2,\mathrm{Pip},2,3,4}$), 2.59-2.75 (br; C$H_{2,\mathrm{Pip},1,5}$), 6.96-7.06 (m; NPC$H_{\mathrm{Ph},ortho,para}$), 7.14-7.21 (m; SPC$H_{\mathrm{Ph},ortho,para}$), 7.56-7.64 (m; SPC$H_{\mathrm{Ph},meta}$), 7.64-7.71 (m; NPC$H_{\mathrm{Ph},meta}$) ppm. ^{31}P{^{1}H} NMR (162.1 MHz, THF-d_8): δ = 9.2 (d, $^{2}J_{PP}$ = 32.5 Hz; S*P*Ph$_2$), 25.4 (d, $^{2}J_{PP}$ = 32.5 Hz; N*P*Ph$_2$) ppm. ^{13}C{^{1}H}-NMR (100.7 MHz, THF-d_8): 127.1 (d, $^{3}J_{CP}$ = 11.4 Hz; NP*C*H$_{\mathrm{Ph},ortho}$), 127.9 (d, $^{4}J_{CP}$ = 2.8 Hz; NP*C*H$_{\mathrm{Ph},para}$), 128.1 (d, $^{3}J_{CP}$ = 11.4 Hz; SP*C*H$_{\mathrm{Ph},ortho}$), 129.4 (d, $^{4}J_{CP}$ = 2.7 Hz; SP*C*H$_{\mathrm{Ph},para}$), 132.2 (d, $^{2}J_{CP}$ = 11.0 Hz; NP*C*H$_{\mathrm{Ph},meta}$), 132.6 (d, $^{2}J_{CP}$ = 9.0 Hz; SP*C*H$_{\mathrm{Ph},meta}$), 138.0 (dd, $^{1,3}J_{CP}$ = 105.3 Hz + 11.2 Hz; SP*C*H$_{\mathrm{Ph},ipso}$), 147.2 (dd, $^{1,3}J_{CP}$ = 74.8 Hz + 3.7 Hz; NP*C*H$_{\mathrm{Ph},ipso}$) ppm. ^{7}Li NMR spectroscopic studies only showed a signal at δ = 0.93, which we assign to excessive butyllithium. Due to the facile re-protonation of the yldiide in THF solution, spectra could only be recorded from the reaction mixture. Several attempts to locate any further signal failed.

[Y$_2$Li][Li(12-C-4)$_2$]:

^{31}P{^{1}H}-NMR (162.1 MHz, Benz-d_6): δ = 21.9 (d, $^{2}J_{PP}$ = 48.0 Hz; S*P*Ph$_2$), 52.9 (d, $^{2}J_{PP}$ = 48.0 Hz; N*P*Ph$_2$) ppm.

1.7 Synthesis of metalated Ylide Y-Na

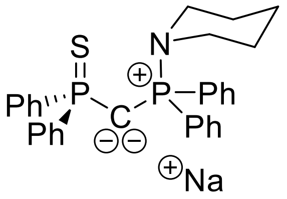

30 mg (0.06 mmol) Y-H and 9.4 mg (0.24 mmol) sodium amide were dissolved in 5 mL THF at room temperature and stirred for 16 h. A color change to yellow was observed. Although ^{31}P NMR spectroscopy showed the successful formation of the yldiide, isolation of the complex failed, due to the fast re-protonation to the ylide. NMR spectra were thus directly recorded after $NaNH_2$ addition.

^{1}H-NMR (400.3 MHz, THF-d_8): δ = 1.26-1.41 (br; C$H_{2,Pip,2,3,4}$), 2.55-2.74 (br; C$H_{2,Pip,1,5}$), 6.95-7.04 (m; NPC$H_{Ph,ortho,para}$), 7.13-7.19 (m; SPC$H_{Ph,ortho,para}$), 7.69-7.78 (m; SPC$H_{Ph,meta}$), 7.78-7.86 (m; NPC$H_{Ph,meta}$) ppm. ^{31}P{^{1}H}-NMR (162.1 MHz, THF-d_8): δ = 7.4 (d, $^{2}J_{PP}$ = 48.7 Hz; S*P*Ph$_2$), 24.1 (d, $^{2}J_{PP}$ = 48.7 Hz; N*P*Ph$_2$) ppm. ^{13}C{^{1}H}-NMR (100.7 MHz, THF-d_8): δ = 26.3 (s; N*C*$H_{Pip,3}$), 27.5 (d, $^{3}J_{CP}$ = 7.9 Hz; N*C*$H_{Pip,2,4}$), 47.0 (s; N*C*$H_{Pip,1,5}$), 127.0 (d, $^{3}J_{CP}$ = 11.3 Hz; NP*C*$H_{Ph,ortho}$), 127.6 (d, $^{4}J_{CP}$ = 2.8 Hz; NP*C*$H_{Ph,para}$), 128.0 (d, $^{3}J_{CP}$ = 11.4 Hz; SP*C*$H_{Ph,ortho}$), 129.1 (d, $^{4}J_{CP}$ = 2.7 Hz; SP*C*$H_{Ph,para}$), 132.5 (d, $^{2}J_{CP}$ = 10.6 Hz; NP*C*$H_{Ph,meta}$), 132.8 (d, $^{2}J_{CP}$ = 8.9 Hz; SP*C*$H_{Ph,meta}$), 139.3 (dd, $^{1,3}J_{CP}$ = 106.0 Hz + 10.6 Hz; SP*C*$H_{Ph,ipso}$), 148.5 (dd, $^{1,3}J_{CP}$ = 78.3 Hz + 7.6 Hz; NP*C*$H_{Ph,ipso}$) ppm.

1.8 Synthesis of metalated Ylide Y-K

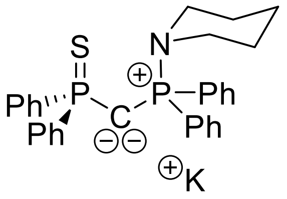

30 mg (0.06 mmol) Y-H and 8.21 mg (0.06 mmol) BnK were dissolved in 1 mL benzene and stirred for 2 min. A color change to yellow could be observed. After filtration and evaporation of the solvent the product was obtained as red solid (29.7 mg, 0.055 mmol; 92 %). Single crystals suitable for X-ray diffraction analyses were grown by two different ways. First, by slow evaporation of a saturated solution of **Y-K** in THF at −30 °C and second by adding a small amount of 18-crown-6 to a saturated solution of **Y-K** in C_6D_6.

Y-K in C_6D_6:

^{1}H-NMR (400.3 MHz, C_6D_6): δ = 1.23-1.34 (br; C$H_{2,Pip,2,3,4}$), 2.70-2.81 (br; C$H_{2,Pip,1,5}$), 6.98-7.17 (m; NPC$H_{Ph,ortho,para}$,SPC$H_{Ph,ortho,para}$), 7.72-7.81 (m; SPC$H_{Ph,meta}$), 8.03-8.12 (m; NPC$H_{Ph,meta}$) ppm. ^{31}P{^{1}H}-NMR (162.1 MHz, C_6D_6): δ = 8.4 (d, $^{2}J_{PP}$ = 62.3 Hz; S*P*Ph$_2$), 18.5 (d, $^{2}J_{PP}$ = 62,4 Hz;

N*P*Ph_2) ppm. $^{13}C\{^1H\}$-NMR (100.7 MHz, C_6D_6): $\delta = 25.5$ (s; N*C*$H_{Pip,3}$), 26.7 (d, $^3J_{CP}$ = 7.59 Hz; N*C*$H_{Pip,2,4}$), 29.1 (dd, $^1J_{CP}$ = 102.0 Hz +81.9 Hz; P*C*P), 46.3 (s; N*C*$H_{Pip,1,5}$), 127.4 (d, $^3J_{CP}$ = 11.4 Hz; NP*C*$H_{Ph,ortho}$), 127.9 (d, $^4J_{CP}$ = 1.99 Hz; NP*C*$H_{Ph,para}$), 128.1 (d, $^3J_{CP}$ = 11.8 Hz; SP*C*$H_{Ph,ortho}$), 128.9 (d, $^4J_{CP}$ = 2.65 Hz; SP*C*$H_{Ph,para}$), 131.4 (d, $^2J_{CP}$ = 10.7 Hz; NP*C*$H_{Ph,meta}$), 131.8 (d, $^2J_{CP}$ = 9.1 Hz; SP*C*$H_{Ph,meta}$), 137.4 (dd, $^{1,3}J_{CP}$ = 104.7 Hz + 9.73Hz; SP*C*$H_{Ph,ipso}$), 147.2 (dd, $^{1,3}J_{CP}$ = 80.54 Hz + 6.62 Hz; NP*C*$H_{Ph,ipso}$) ppm. Anal. Calcd for $C_{30}H_{30}KNP_2S$: C, 67.02; H, 5.62; N, 2.61; S, 5.96. Found: C, 66.64; H, 5.96; N, 2.52; S, 5.60.

Y-K in THF-d*8*:

^1H-NMR (400.3 MHz, THF-d_8): $\delta = 1.24$-1.40 (br; C*H*$_{2,Pip,2,3,4}$), 2.56-2.73 (br; C*H*$_{2,Pip,1,5}$), 6.90-7.24 (m; NPC*H*$_{Ph,ortho,para}$,SPC*H*$_{Ph,ortho,para}$), 7.75-7.86 (m; SPC*H*$_{Ph,meta}$), 7.86-7.98 (m; NPC*H*$_{Ph,meta}$) ppm. $^{31}P\{^1H\}$-NMR (162.1 MHz, THF-d_8): $\delta = 5.34$ (d, $^2J_{PP}$ = 58.76 Hz; S*P*Ph_2), 21.10 (d, $^2J_{PP}$ = 58.74 Hz; N*P*Ph_2) ppm. $^{13}C\{^1H\}$-NMR (100.7 MHz, THF-d_8): $\delta = 26.4$ (s; N*C*$H_{Pip,3}$), 27.5 (d, $^3J_{CP}$ = 8.25 Hz; N*C*$H_{Pip,2,4}$), 30.6 (dd, $^1J_{CP}$ = 99.8 Hz +82.8 Hz; P*C*P), 46.9 (s; N*C*$H_{Pip,1,5}$), 127.0 (d, $^3J_{CP}$ = 11.2 Hz; NP*C*$H_{Ph,ortho}$), 127.4 (d, $^4J_{CP}$ = 2.7 Hz; NP*C*$H_{Ph,para}$), 128.0 (d, $^3J_{CP}$ = 11.3 Hz; SP*C*$H_{Ph,ortho}$), 129.0 (d, $^4J_{CP}$ = 2.7 Hz; SP*C*$H_{Ph,para}$), 132.5 (d, $^2J_{CP}$ = 10.5 Hz; NP*C*$H_{Ph,meta}$), 132.9 (d, $^2J_{CP}$ = 9.0 Hz; SP*C*$H_{Ph,meta}$), 139.7 (dd, $^{1,3}J_{CP}$ = 105.7 Hz + 9.9Hz; SP*C*$H_{Ph,ipso}$), 149.4 (dd, $^{1,3}J_{CP}$ = 80.6 Hz + 9.3 Hz; NP*C*$H_{Ph,ipso}$) ppm.

[Y-K·(18-C-6)]:

Attempts to isolate the crown ether complex by addition of 18-crown-6 to a saturated solution of **Y-K** in THF failed. Resolving of solid material obtained after evaporation of the reaction mixture always resulted in the reformation of the ylide. Also the reaction solution in THF decomposed after several hours.

$^{31}P\{^1H\}$-NMR (162.1 MHz, Benz-d_6): $\delta = 21.9$ (d, $^2J_{PP}$ = 48.0 Hz; S*P*Ph_2), 52.9 (d, $^2J_{PP}$ = 48.0 Hz; N*P*Ph_2) ppm.

2. NMR spectra of the isolated compounds and reaction mixtures

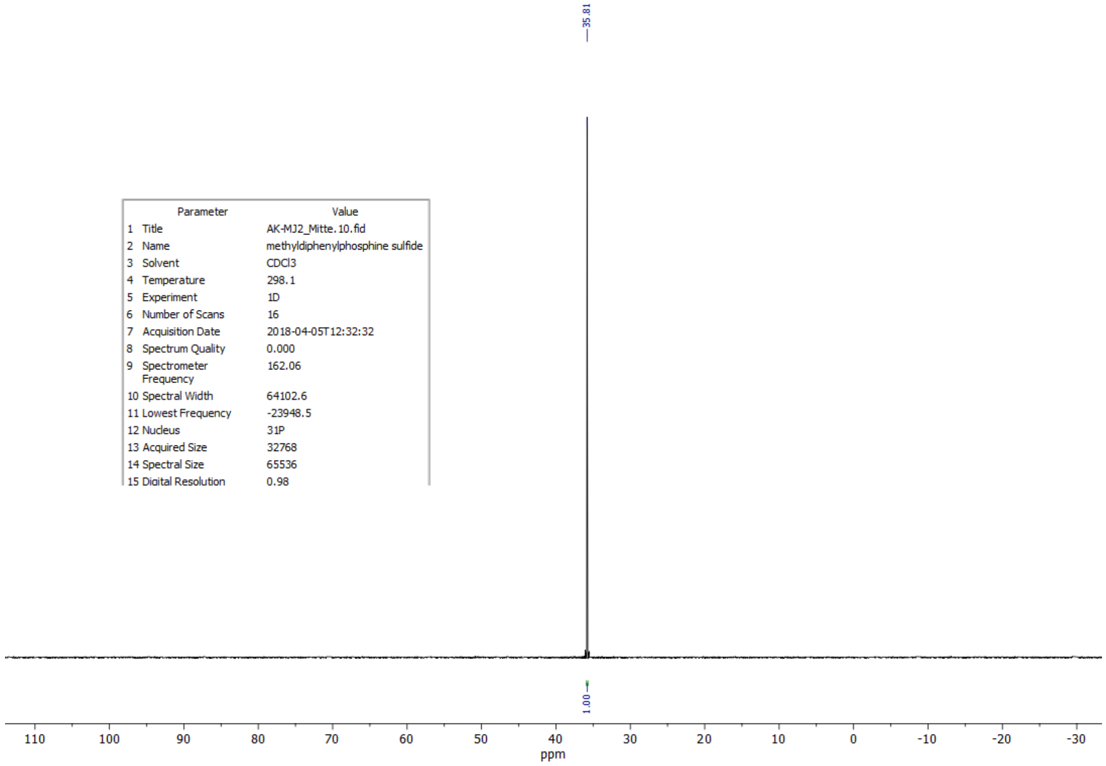

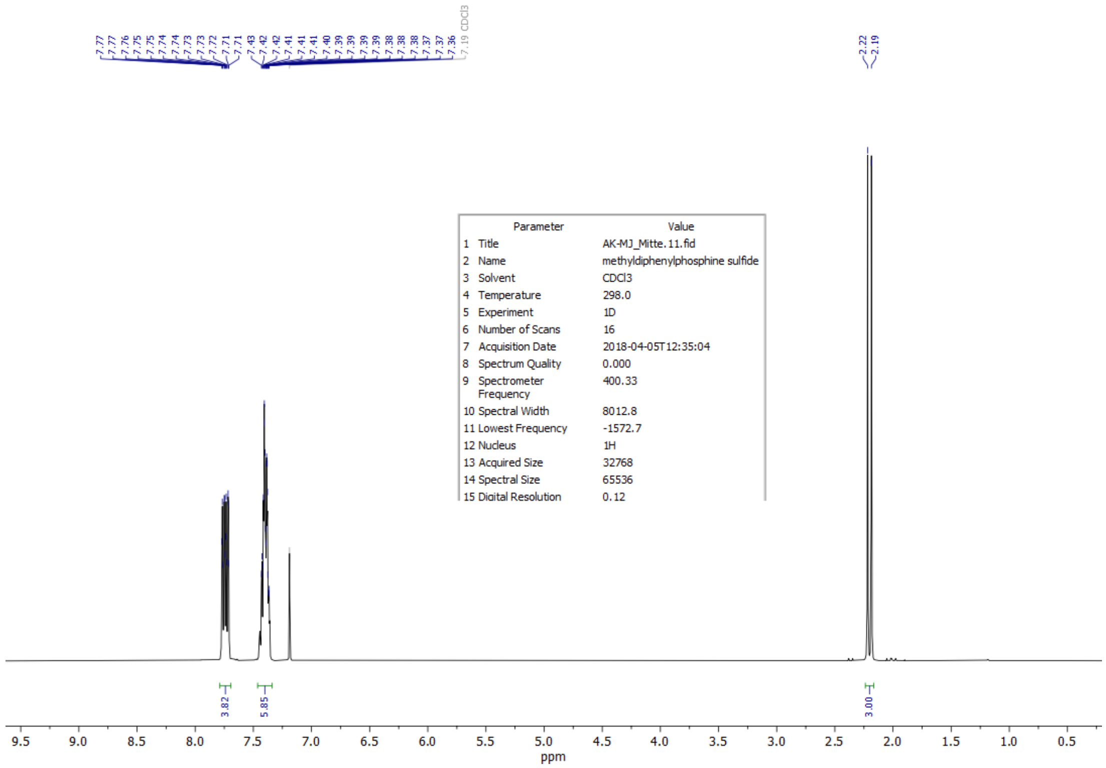

Figure s1 $^{31}P\{^{1}H\}$ NMR and ^{1}H NMR spectrum of compound 1 in $CDCl_3$.

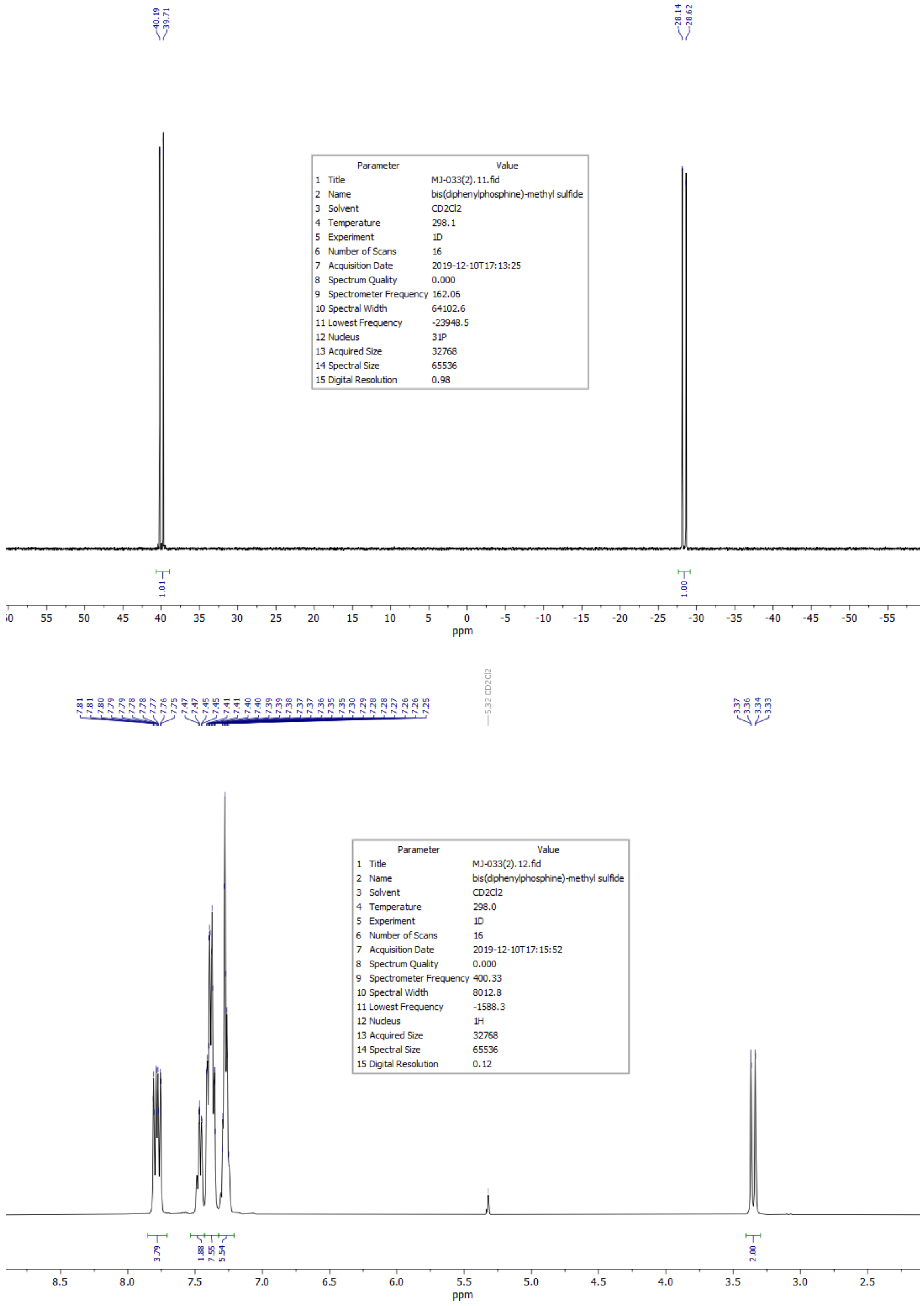

Figure S2 $^{31}P\{^{1}H\}$ NMR and ^{1}H NMR spectrum of compound **2** in CD_2Cl_2.

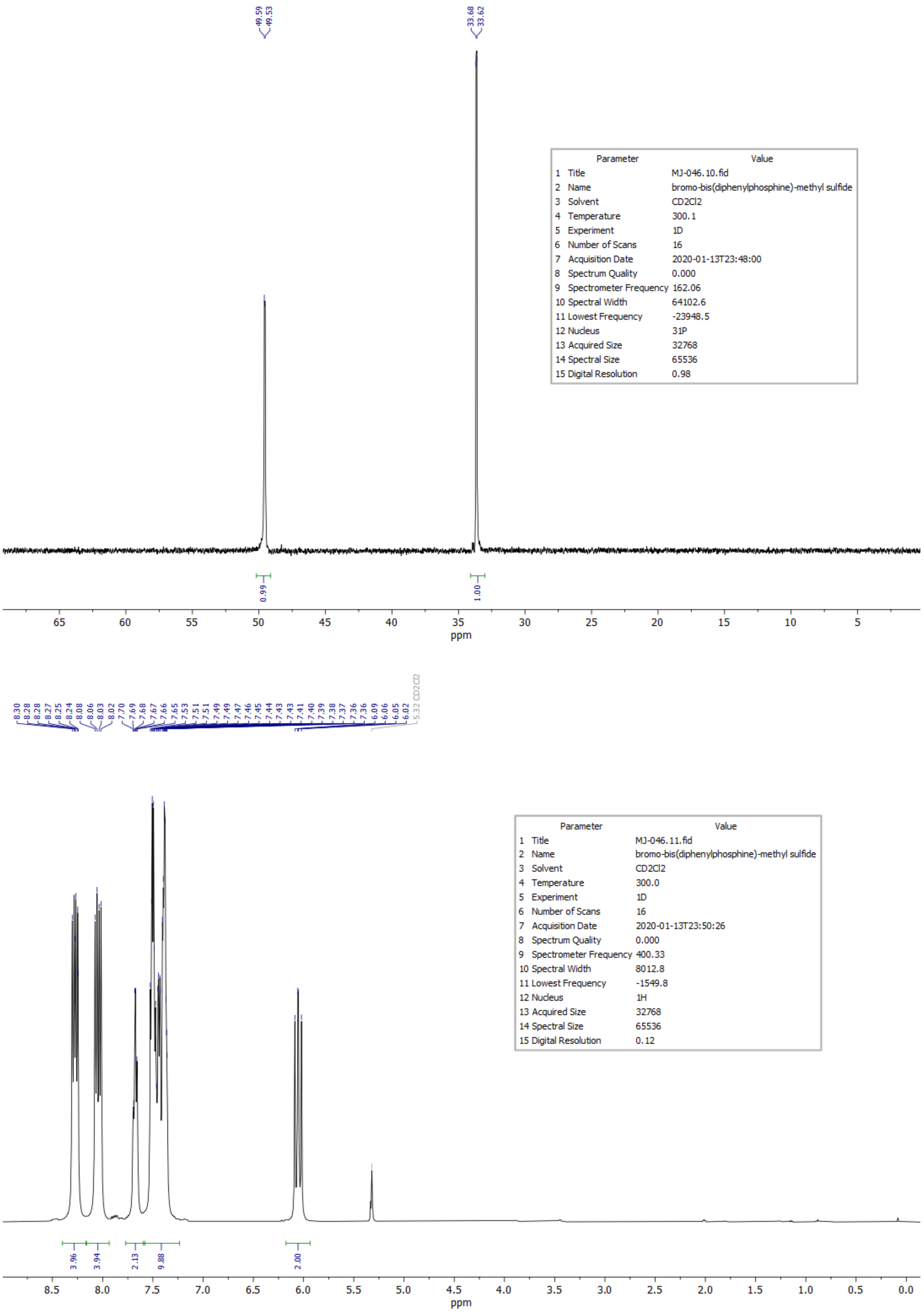

Figure S3a. $^{31}P\{^1H\}$ NMR and ^1H NMR spectra of compound **3** in CD_2Cl_2.

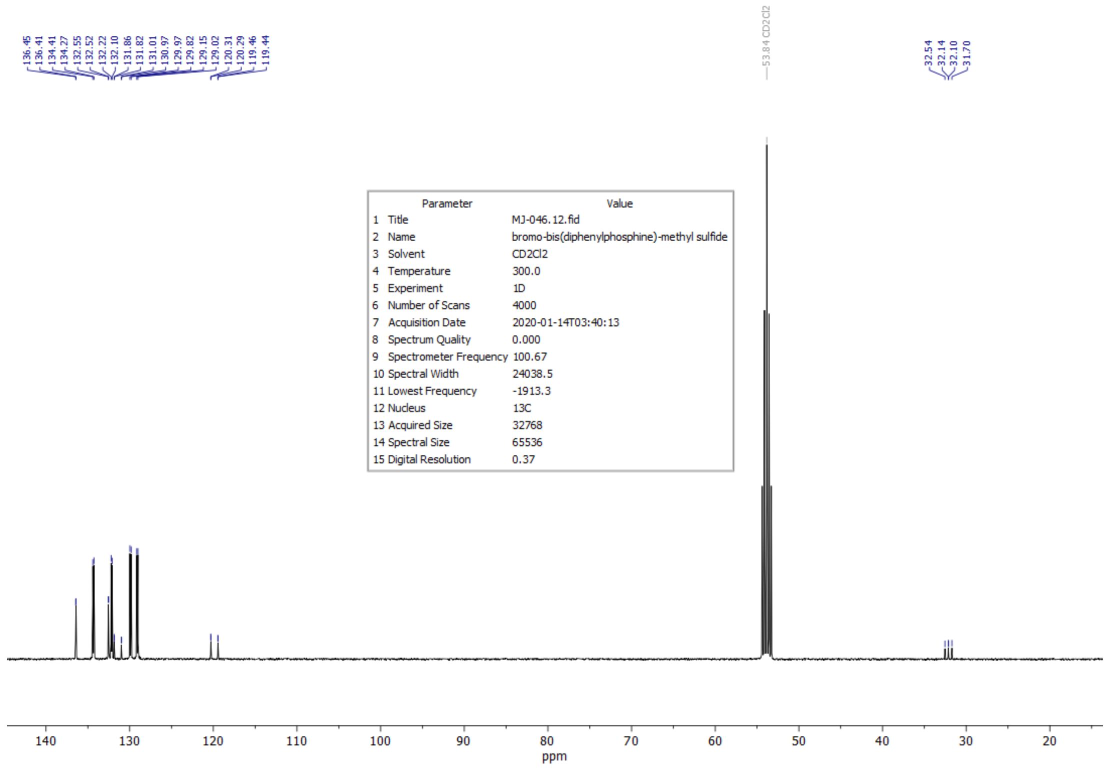

Figure S3b. ^{13}C NMR spectrum of compound **3** in CD_2Cl_2.

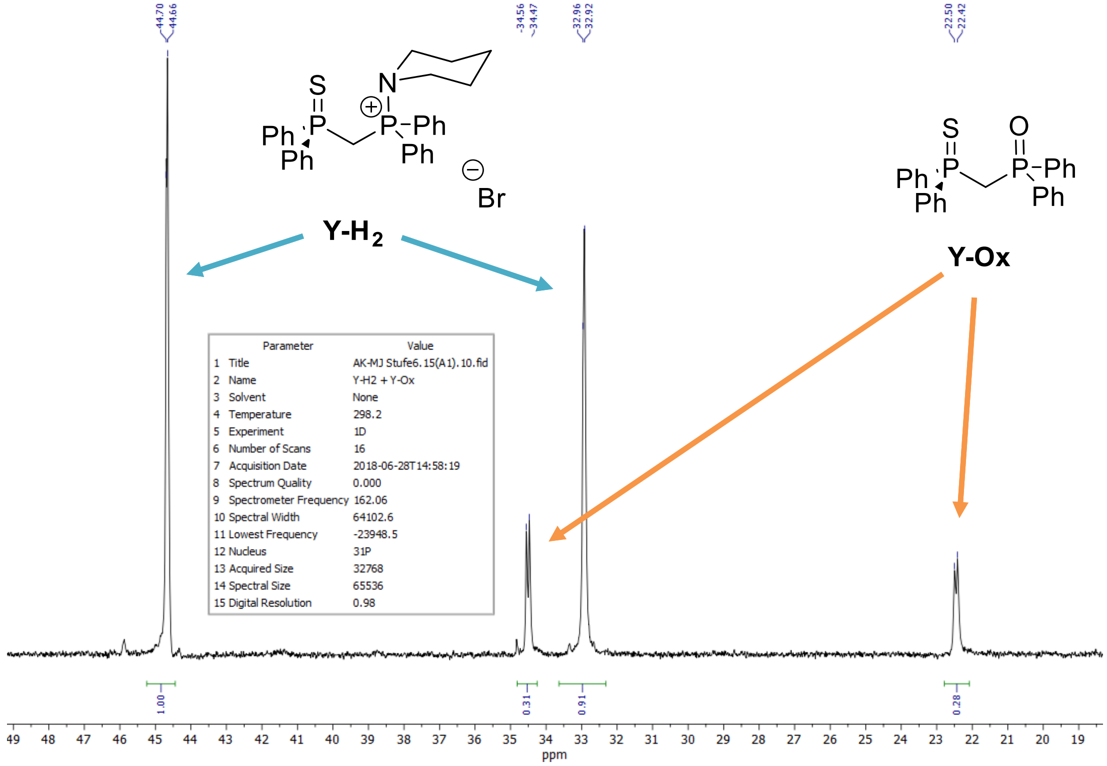

Figure S4 $^{31}P\{^1H\}$ NMR spectrum of Y-H_2 and Y-Ox in THF.

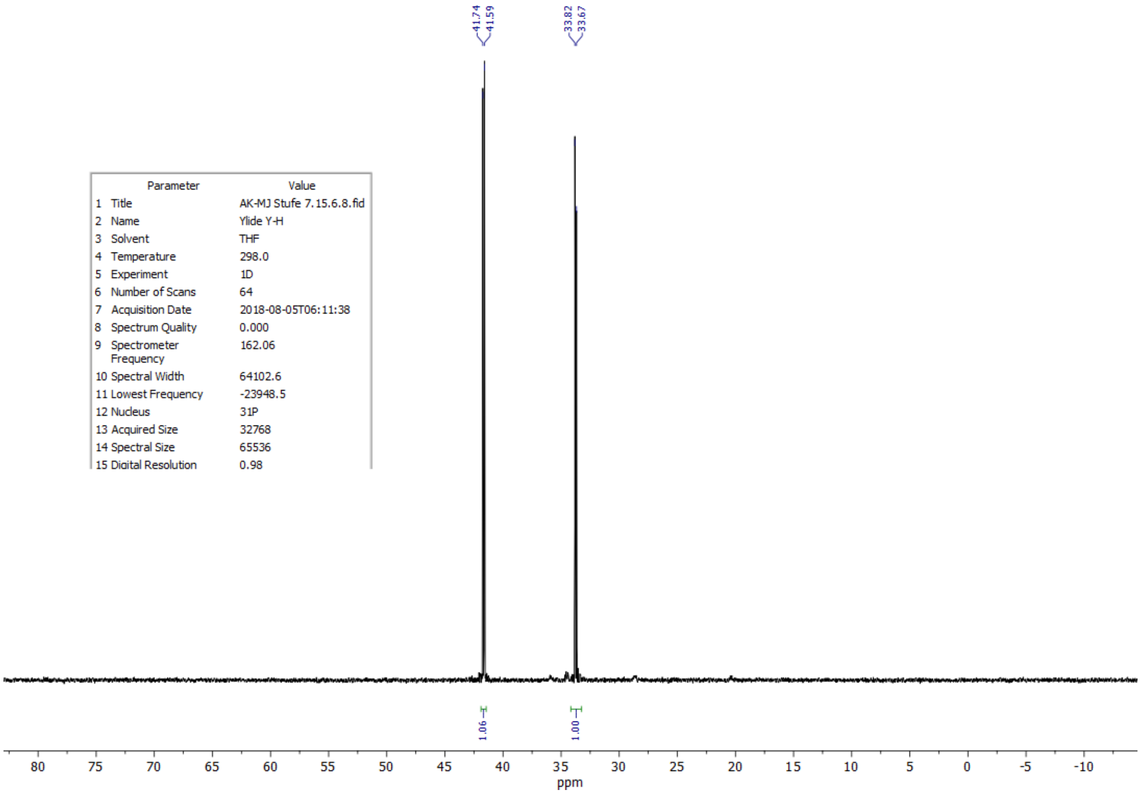

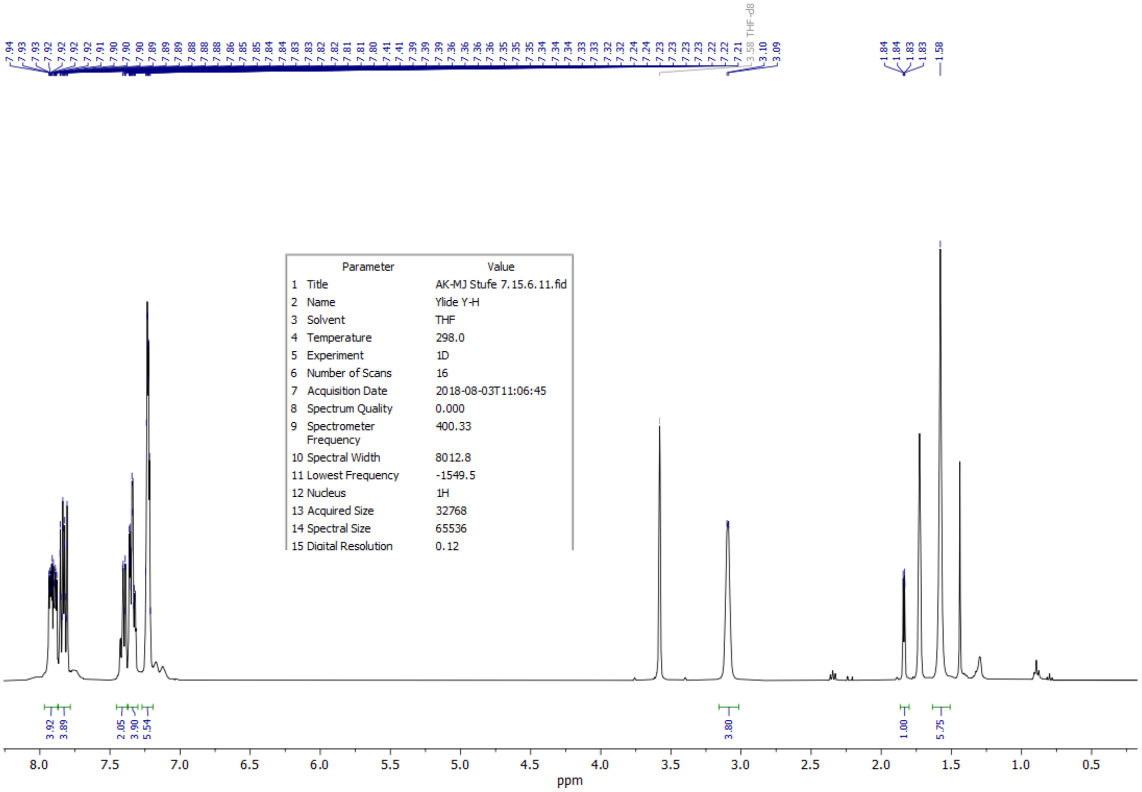

Figure S5a. $^{31}P\{^{1}H\}$ NMR and ^{1}H NMR spectra of Ylide **Y-H** in THF-d_8.

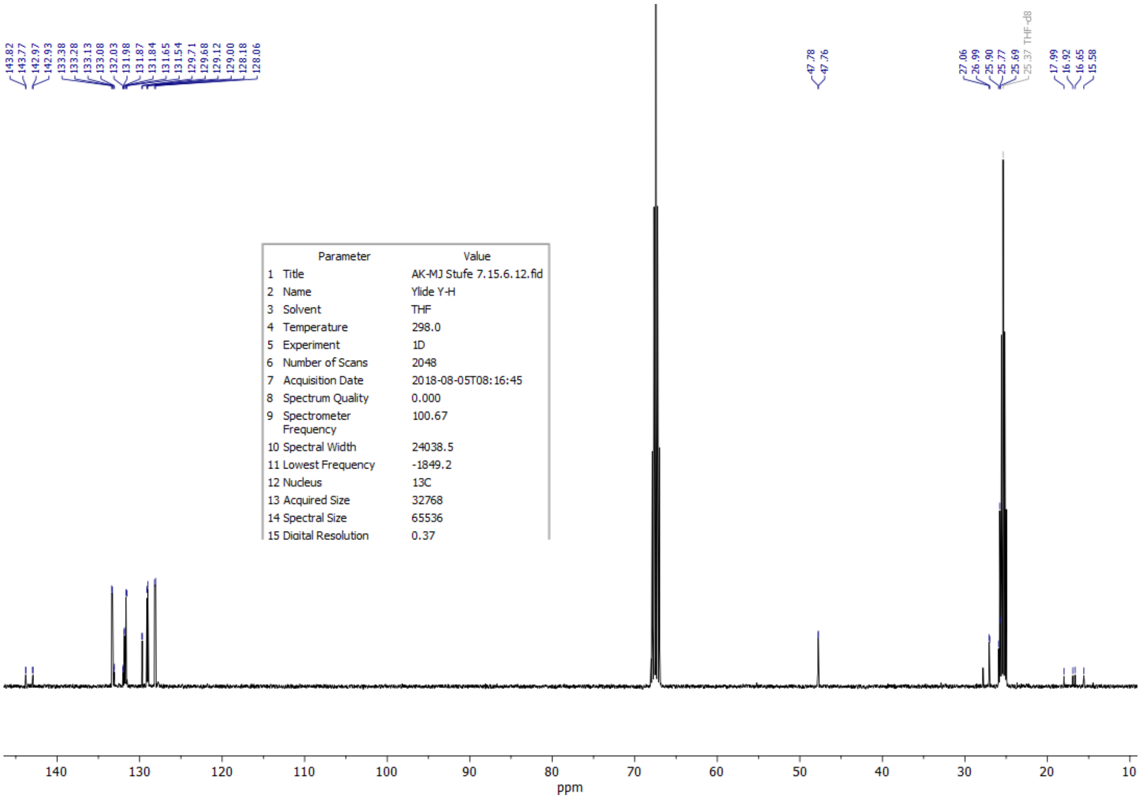

Figure S5b. $^{13}C\{^{1}H\}$ NMR spectrum of Ylide **Y-H** in THF-d_8.

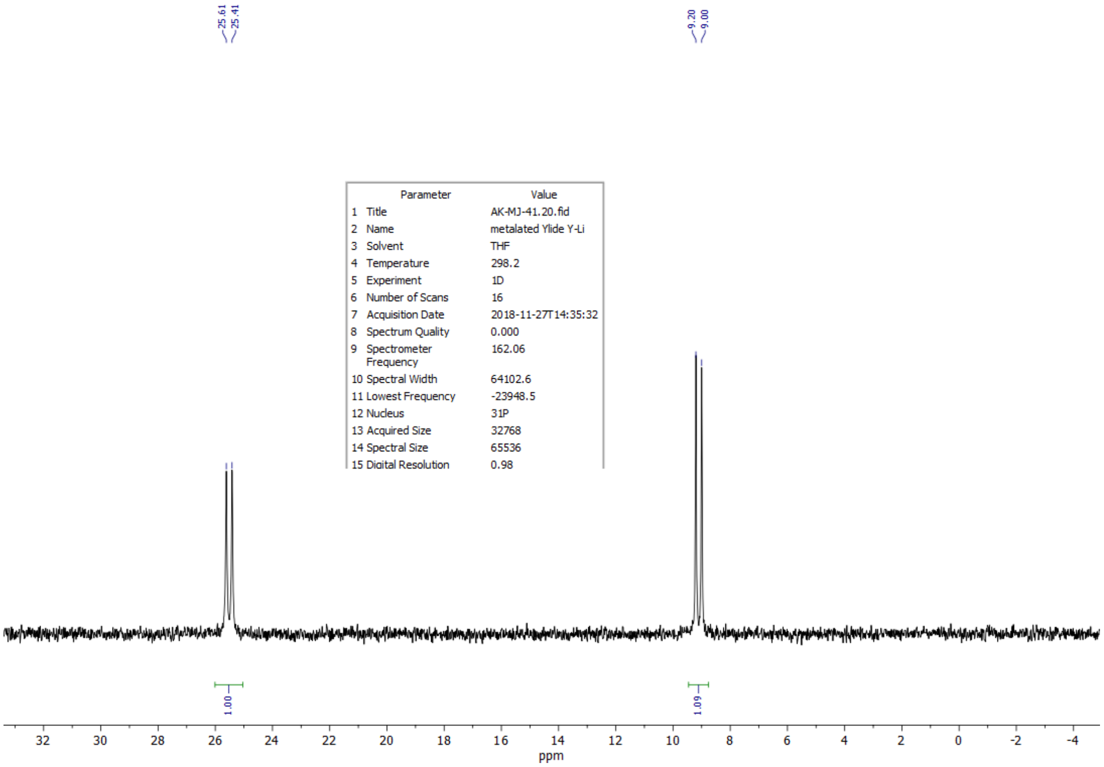

Figure S6a. $^{31}P\{^{1}H\}$ NMR spectrum of the metalated Ylide **Y-Li** in THF-d_8.

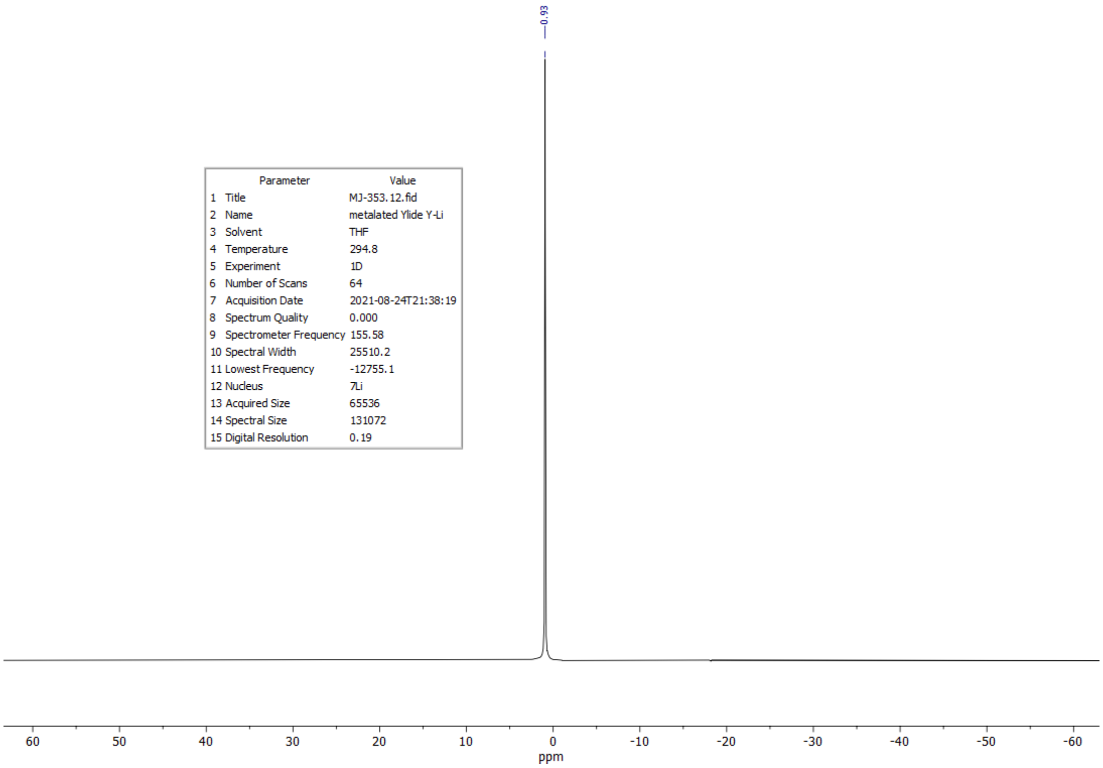

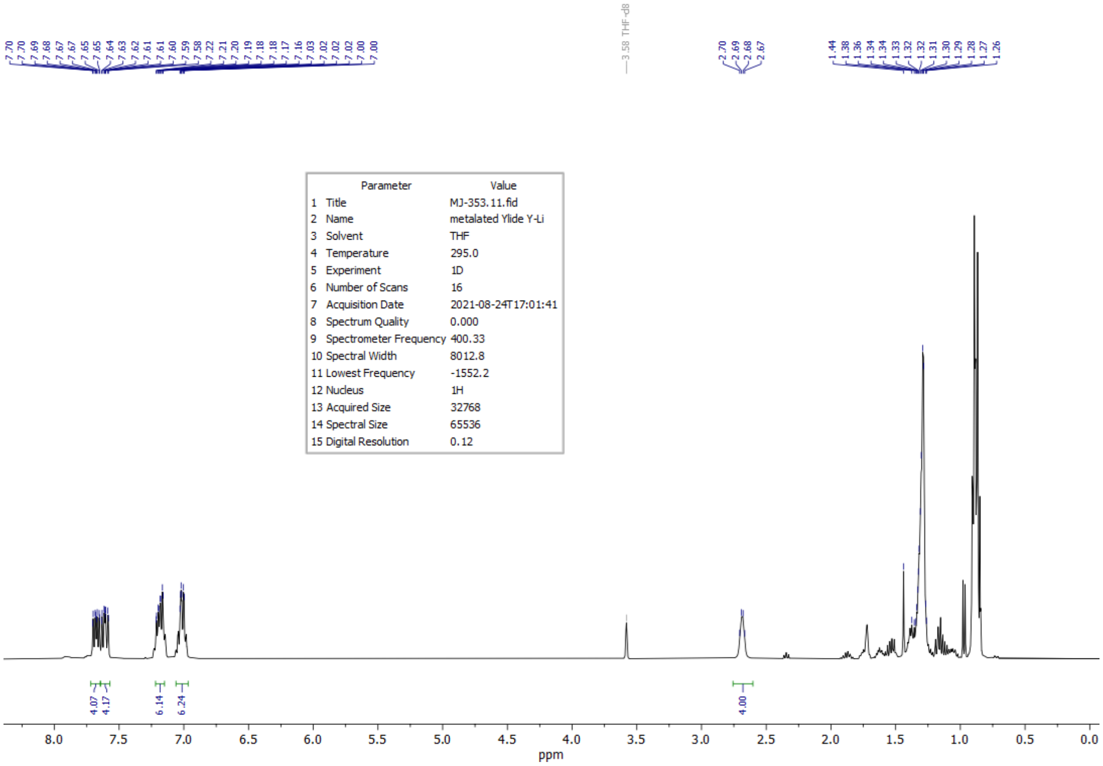

Figure S6b. ^{7}Li and ^{1}H NMR spectra of the metalated Ylide **Y-Li** in THF-d_8. The ^{7}Li NMR presuambly only shows the signal of excessive *n*BuLi. No further signal could be detected.

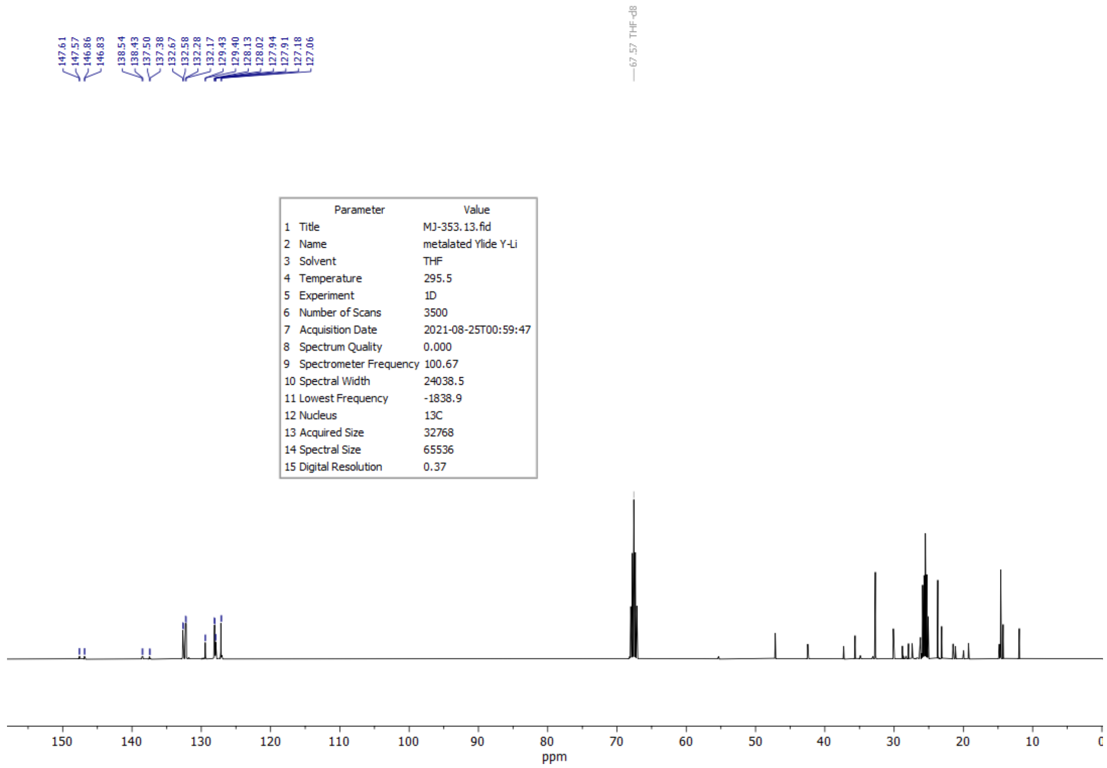

Figure S6c. $^{13}C\{^{1}H\}$ NMR spectrum of metalated Ylide **Y-Li** in THF-d_8. Due to the instability of **Y-Li**, the NMR spectrum was recorded from the reaction solution directly after addition of BuLi. The spectrum thus contains signals of excessive BuLi.

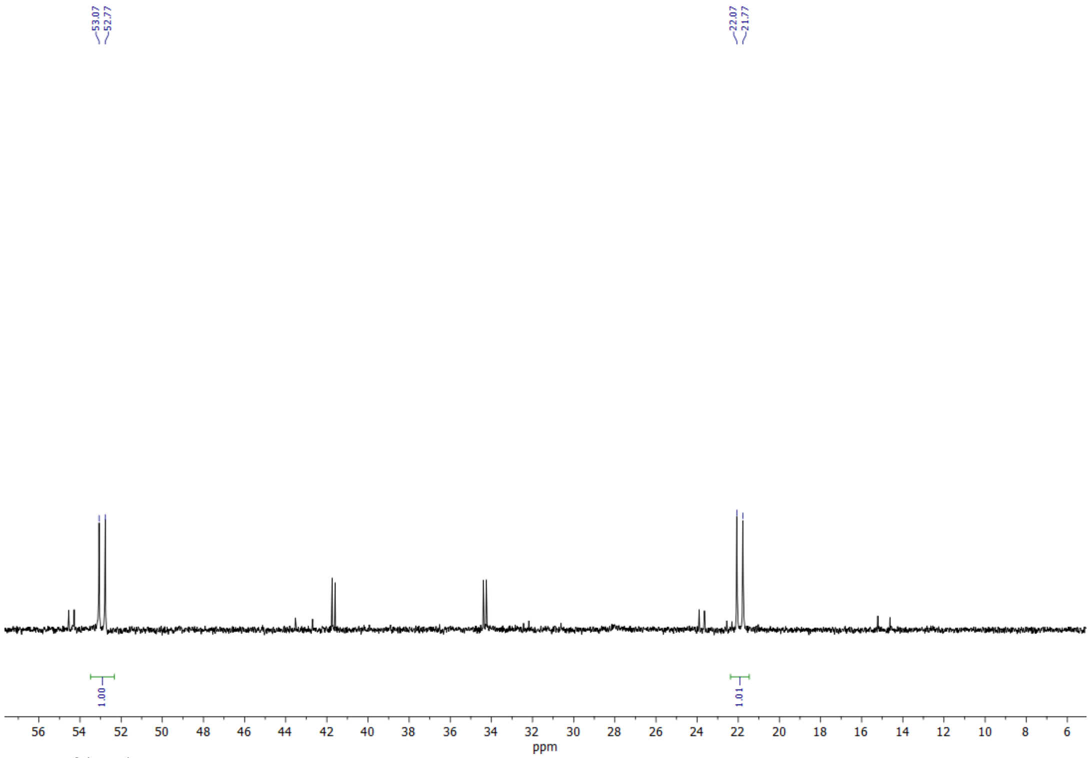

Figure S7. $^{31}P\{^{1}H\}$ NMR spectrum of $[Y_2Li][Li(12\text{-}C\text{-}4)_2]$ in C_6D_6.

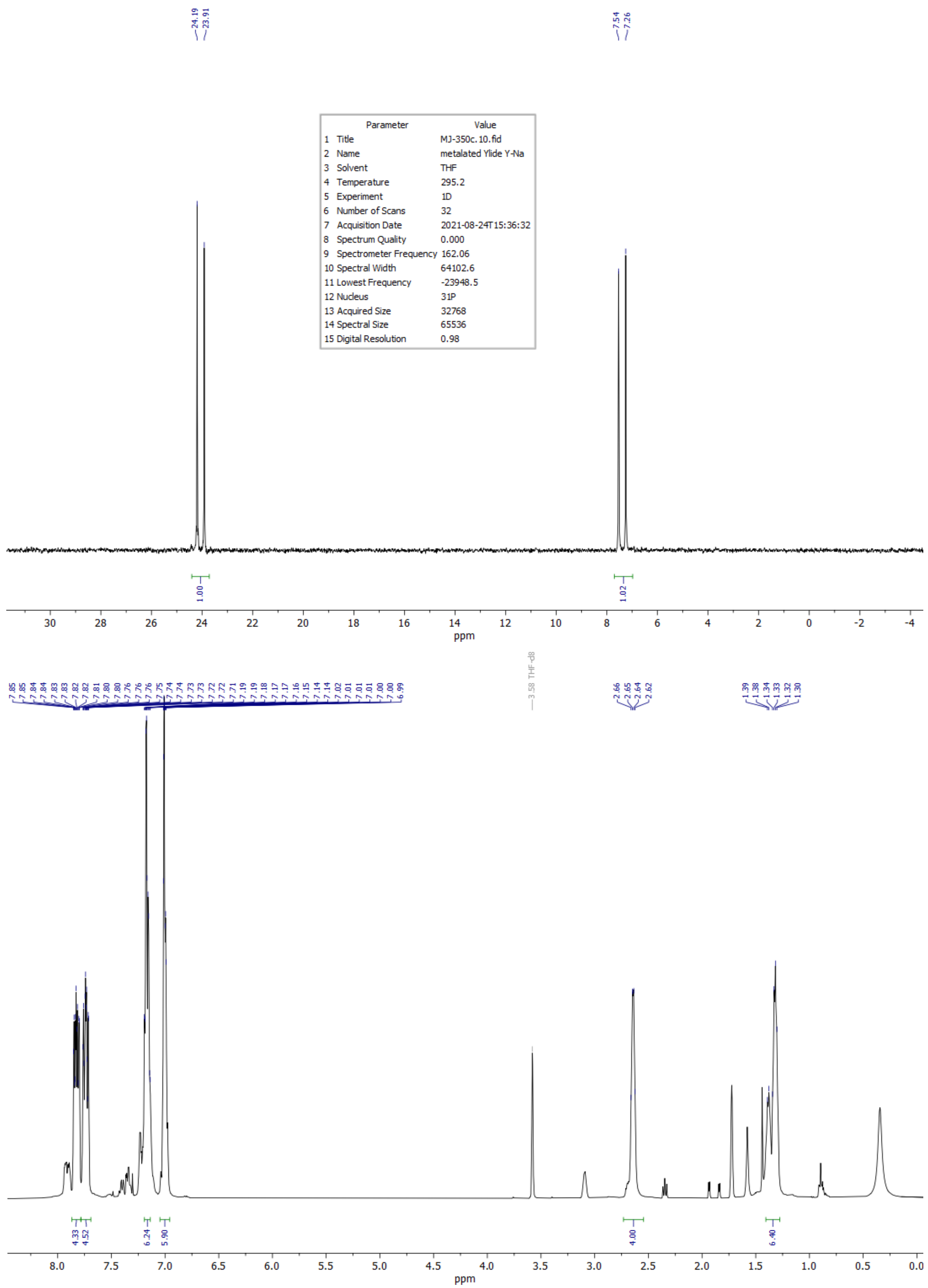

Figure S8a. $^{31}P\{^{1}H\}$ and ^{1}H NMR spectra of the metalated Ylide **Y-Na** in THF-d^{8}.

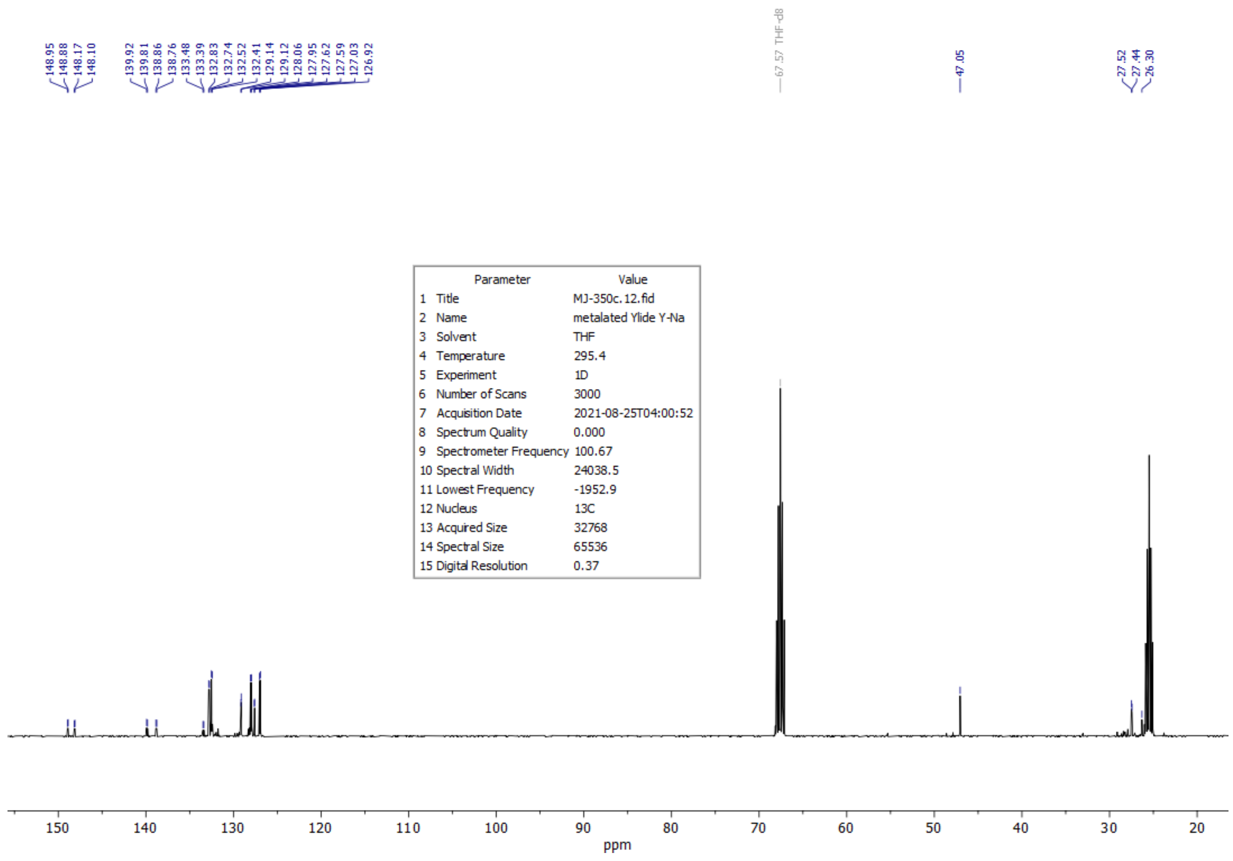

Figure S8b. $^{13}C\{^1H\}$ NMR spectrum of the metalated Ylide **Y-Na** in THF-d_8.

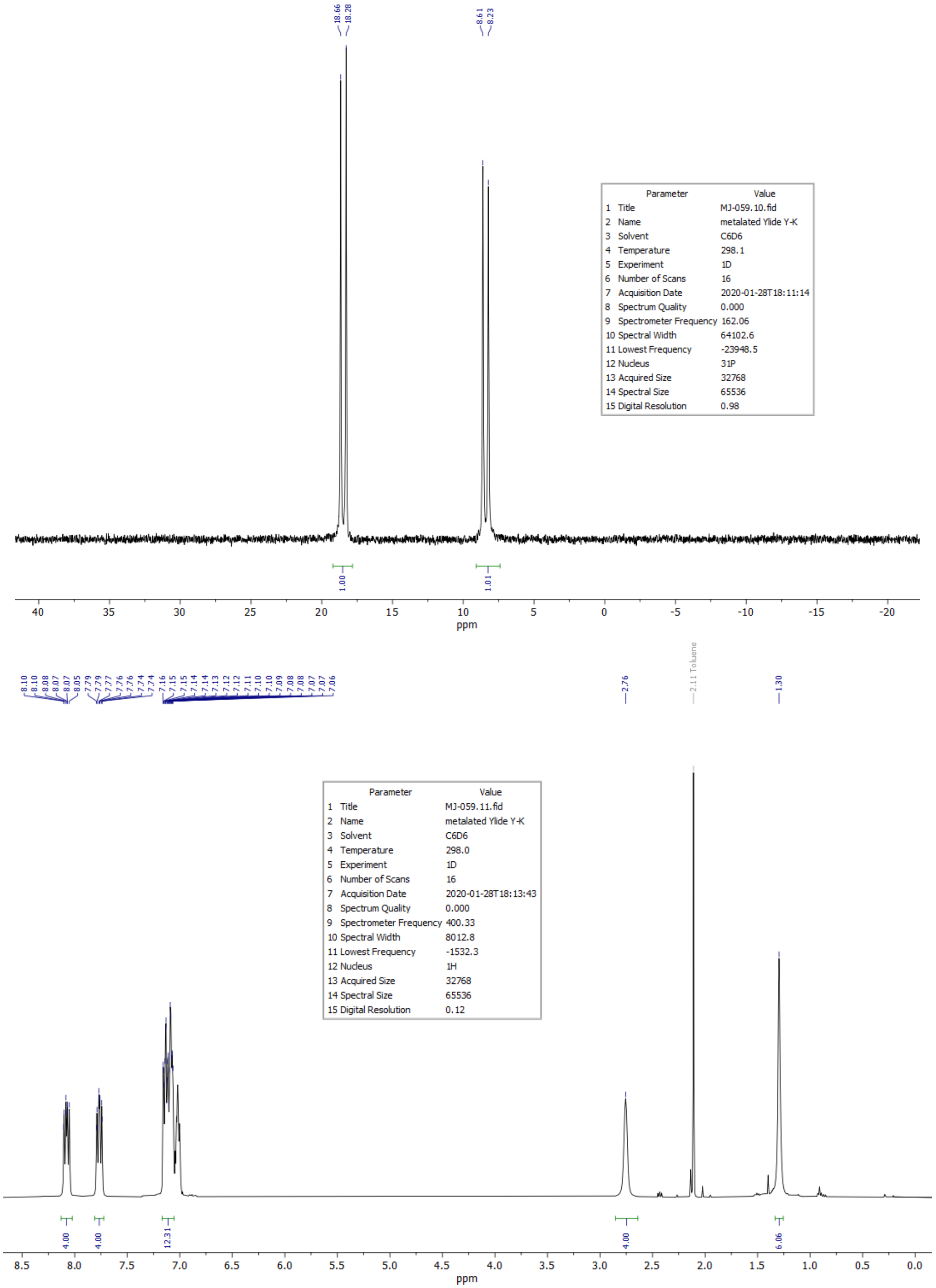

Figure s9a $^{31}P\{^{1}H\}$ NMR and ^{1}H NMR spectra of the metallated Ylide **Y-K** in C_6D_6. The ^{1}H NMR spectrum shows toluene residues which could not be removed. ^{1}H NMR signals of toluene in C_6D_6: $\delta = 2.11$ (s; PhC*H₃*); 6.96-7.09 (m; C*H*$_{Ph}$).

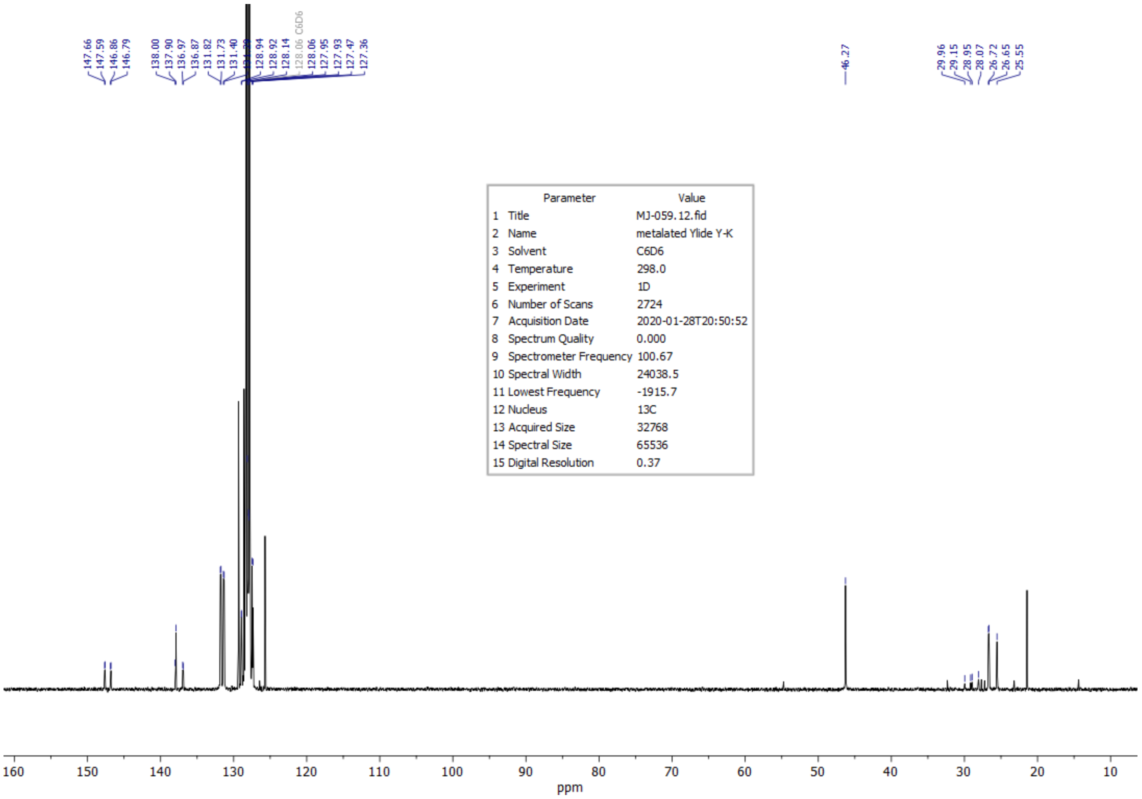

Figure s9b ^{13}C NMR spectrum of the metallated Ylide **Y-K** in C_6D_6. The ^{13}C NMR shows toluene residues which could not be removed. ^{13}C NMR signals of toluene in C_6D_6: $\delta = 21.1$ (s; PhCH_3); 125.7 (s; $CH_{Ph,para}$); 128.5 (s; $CH_{Ph,meta}$); 129.3 (s; $CH_{Ph,ortho}$); 137.9 (s; $CH_{Ph,ipso}$).

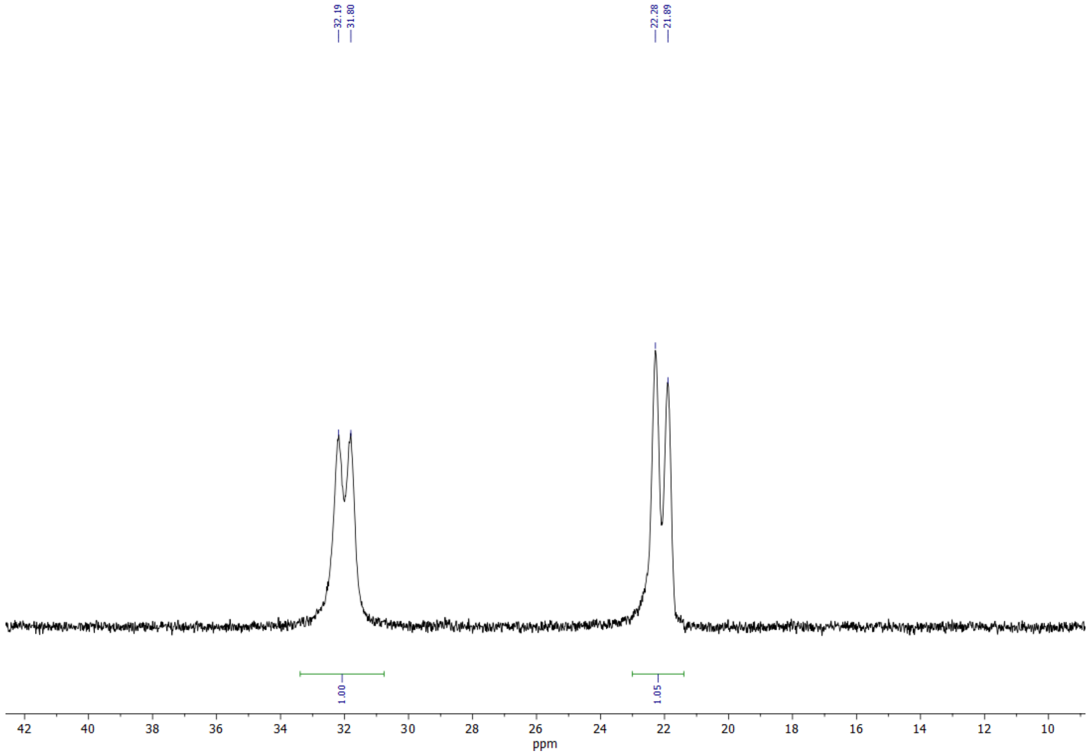

Figure s10. $^{31}P\{^1H\}$ NMR spectrum of [Y-K·(18-C-6)] in C_6D_6.

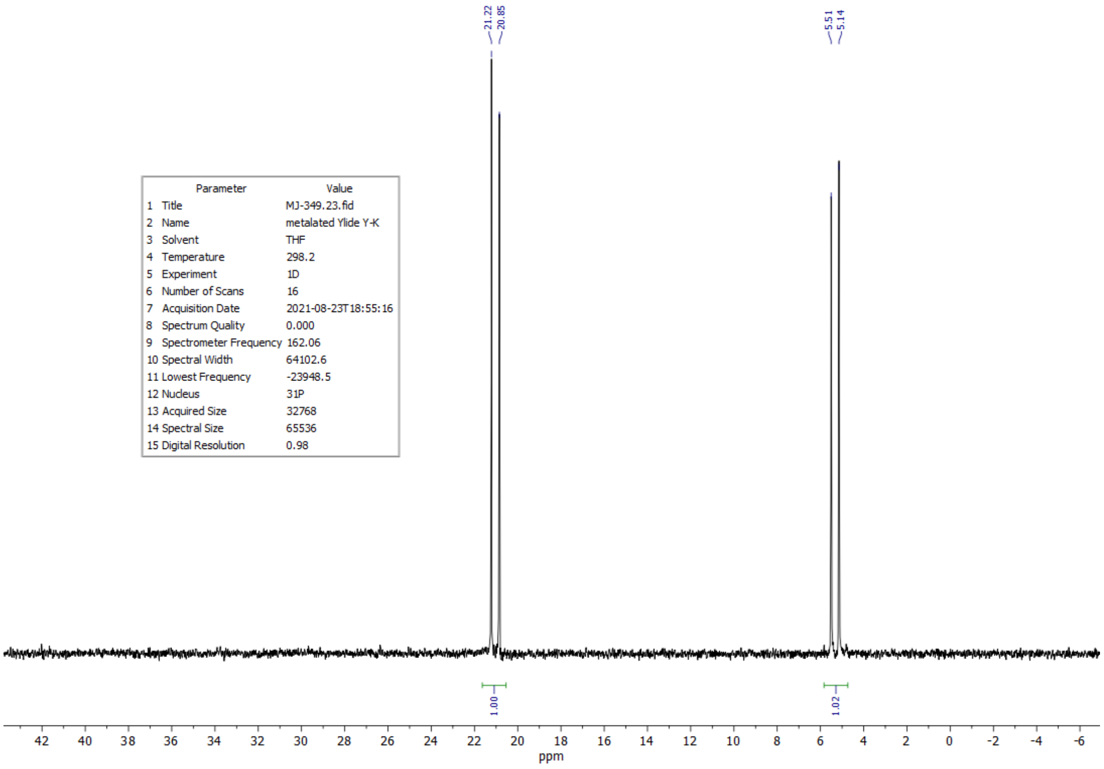

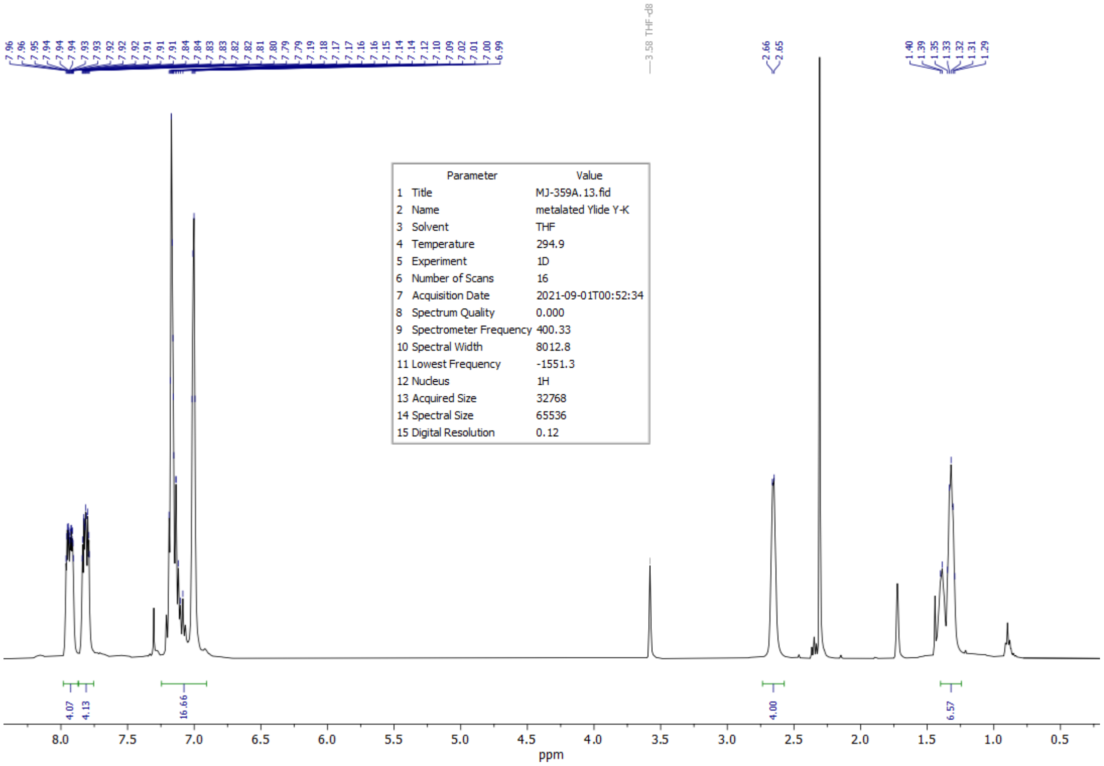

Figure S11a. $^{31}P\{^{1}H\}$ NMR and ^{1}H NMR spectra of the metallated Ylide **Y-K** in THF-d_8. The ^{1}H NMR spectrum shows toluene residues which could not be removed. ^{1}H NMR signals of toluene in C_6D_6: δ = 2.31 (s; PhCH_3); 7.10-7.20 (m; CH_{Ph}).

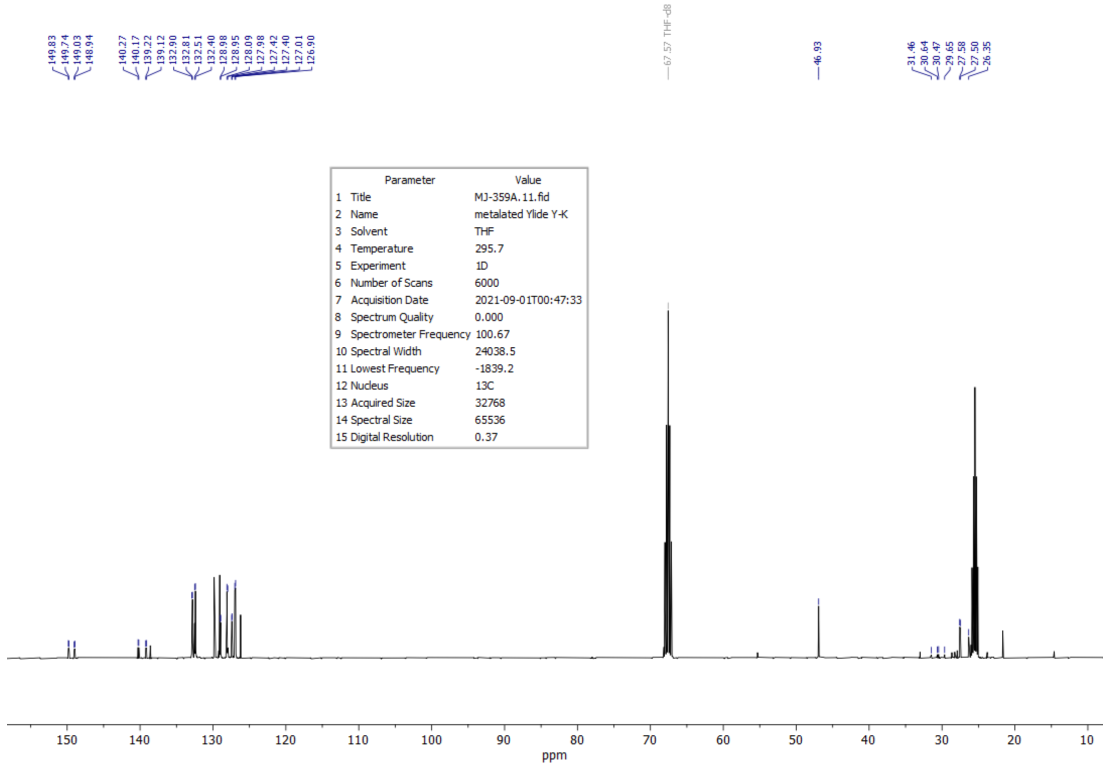

Figure S11b ^{13}C NMR spectrum of the metallated Ylide **Y-K** in THF-d_8. The ^{13}C NMR shows toluene residues which could not be removed. ^{13}C NMR signals of toluene in C_6D_6: $\delta = 21.3$ (s; PhCH_3); 125.8 (s; $CH_{Ph,\textit{para}}$); 128.7 (s; $CH_{Ph,\textit{meta}}$); 129.5 (s; $CH_{Ph,\textit{ortho}}$); 138.2 (s; $CH_{Ph,\textit{ipso}}$).

3. Crystal structure determination.

3.1 General information

Good quality single crystals were hand-picked under polarized optical microscopy and then mounted on the diffractometer. The data collection was done at 100-150 K. X-ray intensity data measurements of **3**, **Y-H2, Y-H, (Y-Li·THF)$_2$, [Y$_2$Li][Li(12-C-4)$_2$], (Y-K)$_6$,** and **[Y-K·(18-C-6)]** were carried out on an Oxford SuperNova diffractometer with graphite-monochromatized (CuK_α= 1.54184 Å) radiation. The X-ray generator was operated at 50 kV and 30 mA. All the structures were solved using direct methods, refined with the Shelx software package[3-4] and expanded using Fourier techniques. The crystals of all compounds were mounted in an inert oil such as perfluoropolyalkylether. Crystallographic data including structure factors have been deposited with the Cambridge Crystallographic Data Centre as supplementary publication no. CCDC-2100502-2100508. Copies of the data can be gained free of charge on application to Cambridge Crystallographic Data Centre, 12 Union Road, Cambridge CB2 1EZ, UK; [fax: (+44) 1223-336-033; email: deposit@ccdc.cam.ac.uk]. An *ORTEP III*[5] view of compounds **2**, **3,** and **4** were drawn with 50% probability displacement ellipsoids and H atoms omitted for clarity.

Table s1 Data collection and structure refinement details for compounds **3**, **Y-H$_2$** and **Y-H**.

**Compound**	**3**	**Y-H$_2$**	**Y-H**
Formula	$C_{25}H_{22}Br_2P_2S$	$C_{39}H_{38}Cl_3F_3NP_2PbS$	$C_{30}H_{31}NP_2S$
CCDC	2100503	2100505	2100504
Formula weight	576.24	985.24	499.56
Temperature [K]	100(2)	100(1)	100(2)
Wave lengt [Å]	1.54184	1.54184	1.54184
Crystal system	monoclinic	triclinic	monoclinic
Space group	$P2_1/c$	P-1	$P2_1/n$
a [Å]	23.0468(3)	9.26700(10)	9.88800(10)
b [Å]	9.16884(14)	14.7431(3)	15.2404(2)
c [Å]	23.0785(3)	14.7885(3)	17.1926(2)
α [°]	90	103.757(2)	90
β [°]	96.8842(13)	92.7240(10)	92.8470(10)
γ [°]	90	97.8100(10)	90
Volumen [Å^3]	4841.60(12)	1937.60(6)	2587.68(5)
Z	8	2	4
Calc. density [Mg·m^{-3}]	1.581	1.689	1.282

μ ($Mo_{K\alpha}$) [mm^{-1}]	6.377	12.020	2.415
F(000)	2304	970	1056
Crystal dimensions [mm]	0.127 × 0.079 × 0.056	0.206 x 0.084 x 0.038	0.196 × 0.175 × 0.88
Theta range θ [°]	3.859 to 74.996	3.087 to 67.072	3.878 to 77.152
Index ranges	-27<=h<=28	-11<=h<=9	-10 ≤ h ≤ 12
	-11<=k<=11	-17<=k<=17	-16 ≤ k ≤ 18
	-22<=l<=28	-17<=l<=17	-21 ≤ l ≤ 21
Reflections collected	37644	21911	20168
Independent reflections	9768 [R(int) = 0.0343]	6886 [R(int) = 0.0584]	5344 [R(int) = 0.0453]
Data/Restraints/Parameter	9768/0/541	6886/0/475	5344/0/307
Goodness-of-fit on F^2	1.123	1.048	1.049
Final R indices [I>2sigma(I)]	R1 = 0.0389, wR2 = 0.1068	R1 = 0.0372, wR2 = 0.0937	R1 = 0.0448, wR2 = 0.1232
Largest diff. peak and hole [e·Å-3]	1.037 and -1.717	1.646 and -1.499	0.384 and -0.522

Table s2 Data collection and structure refinement details for compounds **(Y-Li·THF)$_2$, [Y$_2$Li][Li(12-C-4)$_2$]** and **(Y-K)$_6$**.

**Compound**	**(Y-Li·THF)$_2$**	**[Y$_2$Li][Li(12-C-4)$_2$]**	**(Y-K)$_6$**
Formula	$C_{38}H_{76}Li_2N_2O_2P_4S_2$	$C_{76}H_{92}Li_2N_2O_8P_4S_2$	$C_{180}H_{186}K_6N_6P_{12}S_6$
CCDC	2100508	2100507	2100502
Formula weight	1155.18	1363.39	3231.94
Temperature [K]	100(2)	100(2)	100(1)
Wave lengt [Å]	1.54184	1.54184	1.54184
Crystal system	triclinic	monoclinic	monoclinic
Space group	*P*-1	*P*2$_1$/c	*P*2$_1$/c
a [Å]	10.72830(10)	18.59097(7)	18.09620(10)
b [Å]	13.09820(10	13.14483(5)	18.84310(10)
c [Å]	13.44360(10	29.83655(9)	32.4057(2)
α [°]	66.5770(10)	90	90
β [°]	67.1190(10)	92.2317(3)	104.4140(10)
γ [°]	72.2890(10)	90	90
Volumen [Å^3]	1571.82(3)	7285.78(5)	10702.14(12)
Z	1	4	2
Calc. density [Mg·m^{-3}]	1.220	1.243	1.003
μ (Mo$_{K\alpha}$) [mm^{-1}]	2.073	1.929	2.805
F(000)	612	2896	3396
Crystal dimensions [mm]	0.304 x 0.205 x 0.148	0.344 × 0.124 × 0.048	0.349 x 0.108 x 0.07
Theta range θ [°]	3.737 to 77.444	2.964 to 77.473	2.521 to 67.999
Index ranges	-13 ≤ h ≤ 13	-23<=h<=23	-21 ≤ h ≤ 20
	-16 ≤ k ≤ 16	-16<=k<=16	-22 ≤ k ≤ 22
	-16 ≤ l ≤ 16	-32<=l<=37	-21 ≤ l ≤ 38
Reflections collected	52654	136302	83497
Independent reflections	6583 [R(int) = 0.0337]	15404 [R(int) = 0.0416]	19444 [R(int) = 0.0496]
Data/Restraints/Parameter	6583/143/377	15404/147/904	19444/212/989
Goodness-of-fit on F^2	1.037	1.024	1.061
Final R indices [I>2sigma(I)]	R1 = 0.0534, wR2 = 0.1351	R1 = 0.0519, wR2 = 0.1415	R1 = 0.0527, wR2 = 0.1440
Largest diff. peak and hole [e·Å-3]	1.473 and -1.014	0.657 and -0.501	1.850 and -0.864

Table s3 Data collection and structure refinement details for compound **[Y-K·(18-C-6)].**

**Compound**	**[Y-K·(18-C-6)]**
Formula	$C_{84}H_{108}KNO_{12}P_4S_2$
CCDC	2100506
Formula weight	1603.92
Temperature [K]	100(2)
Wave lengt [Å]	1.54184
Crystal system	triclinic
Space group	*P*-1
a [Å]	9.7118(2)
b [Å]	11.1148(3)
c [Å]	21.2215(5)
α [°]	96.042(2)
β [°]	101.418(2)
γ [°]	108.771(2)
Volumen [Å^3]	2090.45(9)
Z	1
Calc. density [Mg·m^{-3}]	1.274
μ (Mo$_{K\alpha}$) [mm^{-1}]	2.673
F(000)	852
Crystal dimensions [mm]	0.522 x 0.246 x 0.202
Theta range θ [°]	4.273 to 77.663
Index ranges	-10 ≤ h ≤ 12
	-13 ≤ k ≤ 14
	-26 ≤ l ≤ 26
Reflections collected	28673
Independent reflections	8572 [R(int) = 0.0413]
Data/Restraints/Parameter	8572/756/697
Goodness-of-fit on F^2	1.028
Final R indices [I>2sigma(I)]	R1 = 0.0557, wR2 = 0.1463
Largest diff. peak and hole [e·Å-3]	1.215 and -0.456

3.2 Crystal Structure Determination of 3

The B error concerning the short Br-Br distance is probably due to an attractive halogen bond between the P-Br group and the Br counter anion.

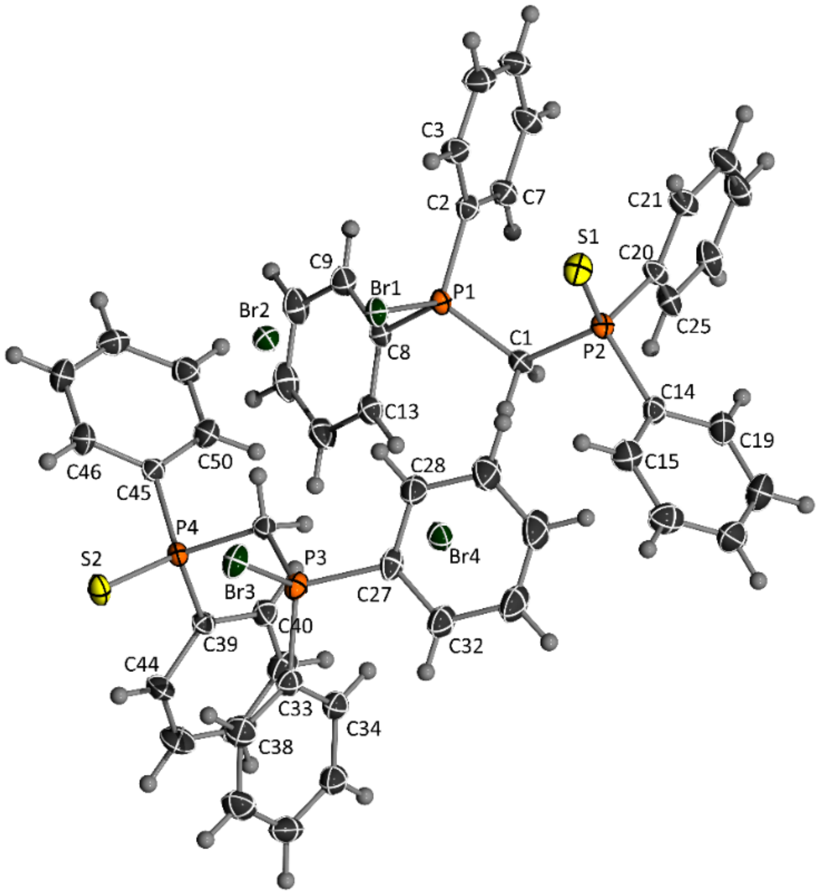

Figure s12 Molecular structure of **3**. Thermal ellipsoids at 50% probability level. Selected bond lengths [Å] and angles [°]: P1–C1 1.798(3), P2–C1 1.835(3), Br1–P1 2.1678(8), S1–P2 1.9522(10), P1–C1–P2 116.82(15).

Table s4 Atomic coordinates (x 10^4) and equivalent isotropic displacement parameters (Å^{2}x 10^3) for compound **3**. U(eq) is defined as one third of the trace of the orthogonalized Uij tensor.

	x	y	z	U(eq)
Br(1)	1488(1)	10742(1)	4845(1)	21(1)
Br(2)	1780(1)	14075(1)	5092(1)	21(1)
Br(3)	3597(1)	5976(1)	5186(1)	27(1)
Br(4)	3223(1)	9296(1)	4874(1)	25(1)
S(1)	1229(1)	9499(1)	3278(1)	25(1)
S(2)	3670(1)	4717(1)	6734(1)	24(1)

P(1)	1342(1)	8407(1)	4800(1)	17(1)
P(2)	1602(1)	7670(1)	3548(1)	17(1)
P(3)	3721(1)	3631(1)	5216(1)	21(1)
P(4)	3357(1)	2864(1)	6418(1)	17(1)
C(1)	1827(1)	7595(3)	4338(1)	17(1)
C(2)	598(1)	7984(3)	4546(1)	20(1)
C(3)	195(1)	9058(3)	4339(1)	24(1)
C(4)	-369(1)	8644(4)	4139(2)	31(1)
C(5)	-538(1)	7190(4)	4140(1)	29(1)
C(6)	-132(1)	6124(4)	4340(1)	27(1)
C(7)	438(1)	6511(3)	4545(1)	23(1)
C(8)	1514(1)	7735(3)	5531(1)	19(1)
C(9)	1066(1)	7435(3)	5870(1)	24(1)
C(10)	1209(2)	6926(4)	6440(1)	31(1)
C(11)	1790(2)	6741(4)	6663(1)	32(1)
C(12)	2233(2)	7041(4)	6326(1)	30(1)
C(13)	2100(1)	7534(3)	5757(1)	24(1)
C(14)	2283(1)	7358(3)	3252(1)	22(1)
C(15)	2675(2)	8526(4)	3280(1)	30(1)
C(16)	3191(2)	8395(4)	3033(2)	36(1)
C(17)	3318(2)	7116(5)	2760(2)	41(1)
C(18)	2934(2)	5979(5)	2721(2)	42(1)
C(19)	2413(2)	6091(4)	2962(2)	33(1)
C(20)	1145(1)	6079(3)	3374(1)	21(1)
C(21)	621(1)	6227(4)	3008(1)	28(1)
C(22)	267(2)	5007(4)	2873(2)	36(1)
C(23)	432(2)	3656(4)	3104(1)	34(1)
C(24)	950(2)	3505(4)	3471(1)	30(1)
C(25)	1306(1)	4714(3)	3602(1)	24(1)
C(26)	3197(1)	2805(3)	5622(1)	19(1)
C(27)	3586(1)	3036(4)	4474(1)	25(1)
C(28)	3014(2)	2945(4)	4208(1)	31(1)
C(29)	2912(2)	2564(5)	3625(1)	40(1)
C(30)	3375(2)	2271(5)	3316(2)	45(1)
C(31)	3944(2)	2346(5)	3580(2)	42(1)
C(32)	4054(2)	2734(4)	4163(1)	32(1)

C(33)	4451(1)	3128(3)	5506(1)	23(1)
C(34)	4592(1)	1651(4)	5462(1)	26(1)
C(35)	5151(2)	1180(4)	5673(1)	31(1)
C(36)	5559(1)	2184(4)	5926(2)	35(1)
C(37)	5406(2)	3628(4)	5984(2)	36(1)
C(38)	4852(2)	4123(4)	5772(2)	31(1)
C(39)	3843(1)	1332(3)	6621(1)	19(1)
C(40)	3739(1)	-49(3)	6378(1)	23(1)
C(41)	4116(2)	-1198(4)	6547(1)	29(1)
C(42)	4591(2)	-970(4)	6964(2)	33(1)
C(43)	4702(2)	392(5)	7205(2)	40(1)
C(44)	4331(1)	1549(4)	7033(1)	29(1)
C(45)	2656(1)	2399(3)	6645(1)	19(1)
C(46)	2408(1)	3279(4)	7039(1)	27(1)
C(47)	1879(2)	2900(4)	7233(1)	32(1)
C(48)	1596(1)	1655(4)	7028(1)	28(1)
C(49)	1823(1)	788(4)	6612(1)	27(1)
C(50)	2347(1)	1163(4)	6413(1)	26(1)

Table s5 Anisotropic displacement parameters (Å^{2}x 10^3) for compound **3**. The anisotropic displacement factor exponent takes the form: –2p^2[h^{2}a*^{2}U^{11} + ... + 2 h k a* b* U^{12}].

	**U^{11}**	**U^{22}**	**U^{33}**	**U^{23}**	**U^{13}**	**U^{12}**
Br(1)	26(1)	16(1)	21(1)	-1(1)	3(1)	-1(1)
Br(2)	23(1)	18(1)	22(1)	2(1)	1(1)	2(1)
Br(3)	37(1)	21(1)	26(1)	2(1)	10(1)	3(1)
Br(4)	27(1)	23(1)	24(1)	-4(1)	2(1)	-3(1)
S(1)	34(1)	20(1)	22(1)	6(1)	4(1)	6(1)
S(2)	30(1)	22(1)	22(1)	-6(1)	3(1)	-7(1)
P(1)	19(1)	16(1)	16(1)	0(1)	2(1)	-2(1)
P(2)	20(1)	16(1)	15(1)	2(1)	2(1)	0(1)
P(3)	25(1)	21(1)	18(1)	1(1)	5(1)	3(1)
P(4)	18(1)	18(1)	14(1)	-3(1)	1(1)	-1(1)
C(1)	18(1)	15(1)	17(1)	1(1)	-1(1)	-1(1)
C(2)	21(1)	23(2)	17(1)	0(1)	4(1)	-1(1)
C(3)	22(1)	23(2)	29(2)	0(1)	4(1)	2(1)

C(4)	23(2)	35(2)	33(2)	3(1)	2(1)	5(1)
C(5)	18(1)	41(2)	27(2)	2(1)	1(1)	-4(1)
C(6)	25(2)	31(2)	24(1)	3(1)	0(1)	-8(1)
C(7)	23(1)	24(2)	22(1)	2(1)	1(1)	-2(1)
C(8)	23(1)	18(1)	18(1)	-2(1)	2(1)	-3(1)
C(9)	27(2)	25(2)	21(1)	-1(1)	6(1)	-6(1)
C(10)	42(2)	30(2)	21(1)	1(1)	7(1)	-9(1)
C(11)	49(2)	27(2)	18(1)	2(1)	0(1)	0(2)
C(12)	35(2)	32(2)	23(1)	-1(1)	-4(1)	4(1)
C(13)	27(2)	21(2)	23(1)	0(1)	1(1)	0(1)
C(14)	25(1)	26(2)	16(1)	-1(1)	4(1)	-1(1)
C(15)	32(2)	30(2)	29(2)	2(1)	6(1)	-2(1)
C(16)	31(2)	45(2)	34(2)	6(2)	6(1)	-2(2)
C(17)	30(2)	63(3)	29(2)	-7(2)	9(1)	-2(2)
C(18)	44(2)	50(2)	34(2)	-12(2)	12(2)	4(2)
C(19)	36(2)	34(2)	29(2)	-9(1)	6(1)	-2(2)
C(20)	21(1)	24(2)	16(1)	2(1)	0(1)	-4(1)
C(21)	26(2)	33(2)	23(1)	2(1)	-2(1)	-2(1)
C(22)	28(2)	49(2)	29(2)	-4(2)	-7(1)	-12(2)
C(23)	36(2)	35(2)	29(2)	-8(1)	2(1)	-17(2)
C(24)	43(2)	23(2)	24(1)	0(1)	3(1)	-9(1)
C(25)	32(2)	21(2)	19(1)	-2(1)	-2(1)	-5(1)
C(26)	20(1)	19(1)	19(1)	-2(1)	1(1)	2(1)
C(27)	35(2)	25(2)	16(1)	2(1)	5(1)	6(1)
C(28)	32(2)	36(2)	23(2)	-2(1)	1(1)	12(1)
C(29)	44(2)	51(2)	23(2)	-2(2)	-2(1)	14(2)
C(30)	57(2)	59(3)	19(2)	-2(2)	1(2)	17(2)
C(31)	47(2)	56(2)	24(2)	0(2)	13(2)	13(2)
C(32)	35(2)	40(2)	23(2)	2(1)	8(1)	7(2)
C(33)	23(1)	27(2)	21(1)	2(1)	6(1)	0(1)
C(34)	25(2)	31(2)	23(1)	-1(1)	4(1)	2(1)
C(35)	30(2)	38(2)	23(2)	-1(1)	3(1)	7(1)
C(36)	22(2)	51(2)	31(2)	4(2)	2(1)	4(2)
C(37)	27(2)	44(2)	38(2)	-1(2)	3(1)	-10(2)
C(38)	30(2)	29(2)	34(2)	-2(1)	6(1)	-5(1)
C(39)	17(1)	25(2)	16(1)	2(1)	1(1)	2(1)

C(40)	26(1)	21(2)	23(1)	-2(1)	0(1)	4(1)
C(41)	33(2)	28(2)	27(2)	5(1)	6(1)	9(1)
C(42)	28(2)	40(2)	32(2)	11(1)	4(1)	14(1)
C(43)	31(2)	52(2)	33(2)	1(2)	-11(1)	10(2)
C(44)	24(2)	35(2)	26(2)	-5(1)	-6(1)	3(1)
C(45)	19(1)	22(1)	16(1)	1(1)	1(1)	3(1)
C(46)	32(2)	22(2)	27(2)	-5(1)	4(1)	1(1)
C(47)	34(2)	40(2)	25(2)	-8(1)	9(1)	3(2)
C(48)	22(1)	35(2)	29(2)	7(1)	5(1)	2(1)
C(49)	24(2)	25(2)	32(2)	-2(1)	2(1)	-1(1)
C(50)	26(2)	26(2)	27(2)	-4(1)	4(1)	3(1)

3.3 Crystal Structure Determination of Y-H$_2$

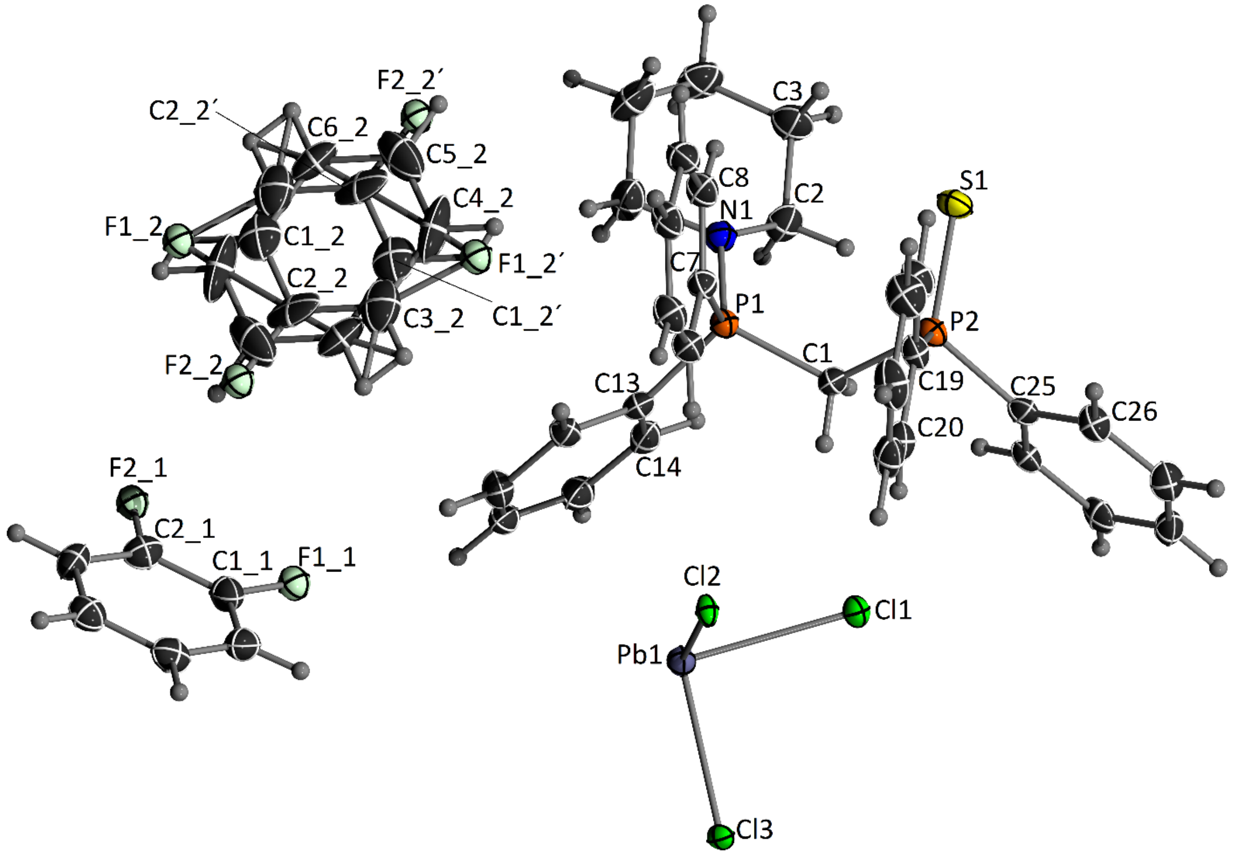

Figure s13 Molecular structure of **Y-H$_2$**. Single crystals suitable for X-ray diffraction analyses of **Y-H$_2$** were obtained by a test reaction of Y-H with $PbCl_2$ in THF. Thermal ellipsoids at 50% probability level. Selected bond lengths [Å] and angles [°]: P1-C1 1.803(5), P2-C1 1.831(5), P1-N1 1.647(4), S1-P2 1.9502(16), P1-C1-P2 119.4(2). Symmetry operations: -x, -y, -z.

Table s6 Atomic coordinates (x 10^4) and equivalent isotropic displacement parameters (Å^{2}x 10^3) for compound **Y-H$_2$**. U(eq) is defined as one third of the trace of the orthogonalized Uij tensor.

	x	y	z	U(eq)
Pb(1)	7453(1)	4974(1)	4848(1)	19(1)
Cl(1)	9538(1)	4974(1)	6169(1)	20(1)
N(1)	10266(4)	9119(3)	7008(3)	22(1)
C(1)	9976(5)	7286(3)	7342(3)	20(1)
S(1)	11424(1)	8632(1)	9268(1)	25(1)
P(1)	9088(1)	8163(1)	6964(1)	19(1)
Cl(2)	5679(1)	5173(1)	6274(1)	15(1)
P(2)	10371(1)	7402(1)	8593(1)	19(1)

C(2)	11869(5)	9160(4)	7086(4)	29(1)
Cl(3)	6952(1)	3107(1)	4261(1)	15(1)
C(3)	12586(6)	10151(4)	7558(4)	35(1)
C(4)	12147(6)	10863(4)	7051(5)	38(1)
C(8)	7758(5)	9378(3)	8256(4)	26(1)
C(7)	7619(5)	8476(3)	7662(3)	20(1)
C(6)	9792(6)	9778(4)	6477(4)	35(1)
C(5)	10500(6)	10772(4)	6918(5)	40(1)
C(9)	6620(5)	9643(4)	8784(4)	27(1)
C(10)	5361(5)	8985(4)	8737(4)	27(1)
C(11)	5240(5)	8076(4)	8154(4)	29(1)
C(12)	6375(5)	7818(4)	7625(4)	26(1)
C(14)	9465(5)	7433(3)	5138(4)	25(1)
C(13)	8408(5)	7650(3)	5761(3)	21(1)
C(16)	7604(6)	7024(3)	3859(4)	27(1)
C(15)	9065(6)	7126(4)	4190(4)	28(1)
C(19)	8635(5)	7106(3)	9044(3)	21(1)
C(18)	6934(5)	7537(3)	5429(3)	23(1)
C(17)	6553(6)	7220(4)	4471(4)	30(1)
C(20)	7769(5)	6235(4)	8639(4)	24(1)
C(21)	6443(5)	6002(4)	8992(4)	29(1)
C(22)	5981(5)	6631(4)	9741(4)	35(1)
C(23)	6837(6)	7491(4)	10141(4)	34(1)
C(24)	8167(5)	7741(4)	9791(4)	28(1)
C(25)	11289(5)	6396(3)	8611(3)	21(1)
C(26)	11134(5)	5988(4)	9378(3)	24(1)
C(27)	11846(5)	5233(4)	9438(4)	27(1)
C(28)	12715(5)	4872(3)	8744(4)	26(1)
C(29)	12886(5)	5276(4)	7997(4)	24(1)
C(30)	12195(5)	6047(3)	7929(3)	20(1)
C11	3636(6)	6990(4)	2086(4)	31(1)
F11	5091(4)	6978(3)	2243(3)	47(1)
C21	3210(6)	7664(4)	1661(4)	28(1)
F21	4250(3)	8283(2)	1429(3)	40(1)
C31	1756(6)	7703(4)	1482(4)	28(1)
C41	718(6)	7051(4)	1735(4)	29(1)

C51	1148(6)	6378(4)	2152(4)	29(1)
C61	2619(6)	6352(4)	2352(4)	31(1)
F12	4498(11)	10602(8)	3663(6)	62(2)
F22	3312(12)	8928(8)	3852(10)	72(3)
C12	4850(11)	10350(9)	4417(6)	40(4)
C22	4250(11)	9472(8)	4532(8)	44(4)
C32	4612(12)	9209(8)	5346(10)	47(5)
C42	5574(13)	9824(12)	6045(7)	52(5)
C52	6175(10)	10702(11)	5930(6)	53(6)
C62	5813(11)	10965(8)	5115(8)	35(3)

Table s7 Anisotropic displacement parameters (Å^{2}x 10^3) for compound **Y-H$_2$**. The anisotropic displacement factor exponent takes the form: –2p^2[h^{2}a*^{2}U^{11} + ... + 2 h k a* b* U^{12}].

	U^{11}	U^{22}	U^{33}	U^{23}	U^{13}	U^{12}
Pb(1)	18(1)	20(1)	20(1)	6(1)	0(1)	3(1)
Cl(1)	17(1)	21(1)	21(1)	4(1)	-1(1)	3(1)
N(1)	21(2)	20(2)	27(2)	6(2)	1(2)	2(2)
C(1)	25(2)	15(2)	20(2)	4(2)	-1(2)	4(2)
S(1)	22(1)	22(1)	26(1)	0(1)	-3(1)	-2(1)
P(1)	18(1)	18(1)	21(1)	5(1)	0(1)	2(1)
Cl(2)	12(1)	21(1)	14(1)	5(1)	1(1)	6(1)
P(2)	17(1)	20(1)	20(1)	4(1)	0(1)	2(1)
C(2)	22(2)	27(3)	41(3)	15(2)	3(2)	2(2)
Cl(3)	11(1)	14(1)	20(1)	2(1)	0(1)	1(1)
C(3)	28(3)	30(3)	44(3)	8(2)	-3(2)	-5(2)
C(4)	35(3)	26(3)	52(4)	13(2)	5(3)	-4(2)
C(8)	18(2)	26(2)	33(3)	8(2)	-3(2)	0(2)
C(7)	21(2)	21(2)	18(2)	4(2)	1(2)	5(2)
C(6)	29(3)	33(3)	46(3)	20(3)	-1(2)	3(2)
C(5)	38(3)	25(3)	63(4)	19(3)	11(3)	8(2)
C(9)	24(2)	28(3)	26(3)	0(2)	-2(2)	5(2)
C(10)	18(2)	34(3)	26(3)	3(2)	0(2)	7(2)
C(11)	21(2)	35(3)	29(3)	7(2)	-1(2)	2(2)
C(12)	25(2)	24(2)	26(3)	5(2)	-3(2)	3(2)
C(14)	25(2)	22(2)	30(3)	10(2)	1(2)	5(2)

C(13)	25(2)	14(2)	23(2)	4(2)	1(2)	4(2)
C(16)	41(3)	20(2)	22(2)	5(2)	-2(2)	7(2)
C(15)	34(3)	25(2)	28(3)	9(2)	3(2)	8(2)
C(19)	18(2)	26(2)	18(2)	7(2)	-2(2)	1(2)
C(18)	21(2)	20(2)	26(3)	4(2)	-1(2)	2(2)
C(17)	29(3)	31(3)	28(3)	6(2)	-6(2)	6(2)
C(20)	20(2)	31(3)	24(2)	11(2)	2(2)	6(2)
C(21)	18(2)	33(3)	38(3)	16(2)	-5(2)	0(2)
C(22)	17(2)	56(4)	34(3)	19(3)	4(2)	5(2)
C(23)	25(3)	46(3)	32(3)	7(2)	7(2)	8(2)
C(24)	26(3)	34(3)	21(2)	3(2)	1(2)	4(2)
C(25)	17(2)	18(2)	25(2)	2(2)	-4(2)	-2(2)
C(26)	20(2)	30(2)	22(2)	7(2)	2(2)	3(2)
C(27)	24(2)	33(3)	27(3)	12(2)	2(2)	1(2)
C(28)	21(2)	25(2)	32(3)	11(2)	-5(2)	3(2)
C(29)	13(2)	32(3)	25(3)	1(2)	-3(2)	4(2)
C(30)	17(2)	24(2)	17(2)	3(2)	-2(2)	-1(2)
C11	27(3)	29(3)	33(3)	1(2)	-5(2)	8(2)
F11	29(2)	48(2)	66(2)	17(2)	-8(2)	10(2)
C21	31(3)	26(2)	28(3)	6(2)	8(2)	5(2)
F21	32(2)	32(2)	60(2)	18(2)	10(2)	1(1)
C31	34(3)	26(2)	29(3)	11(2)	6(2)	12(2)
C41	28(3)	32(3)	25(3)	3(2)	3(2)	5(2)
C51	34(3)	29(3)	23(3)	6(2)	4(2)	-1(2)
C61	43(3)	23(2)	26(3)	4(2)	-1(2)	4(2)
F12	85(6)	85(7)	32(4)	25(4)	7(4)	46(5)
F22	57(7)	58(7)	85(8)	-12(6)	-21(6)	12(5)
C12	38(10)	50(11)	36(10)	6(9)	11(8)	22(8)
C22	24(7)	22(6)	84(14)	4(9)	10(8)	14(6)
C32	40(11)	57(13)	52(12)	18(12)	5(10)	23(10)
C42	51(10)	80(13)	52(11)	50(11)	23(8)	39(9)
C52	29(11)	73(18)	47(12)	-6(11)	-10(8)	13(11)
C62	38(8)	23(7)	52(10)	16(8)	18(7)	15(6)

3.4 Crystal Structure Determination of Y-H

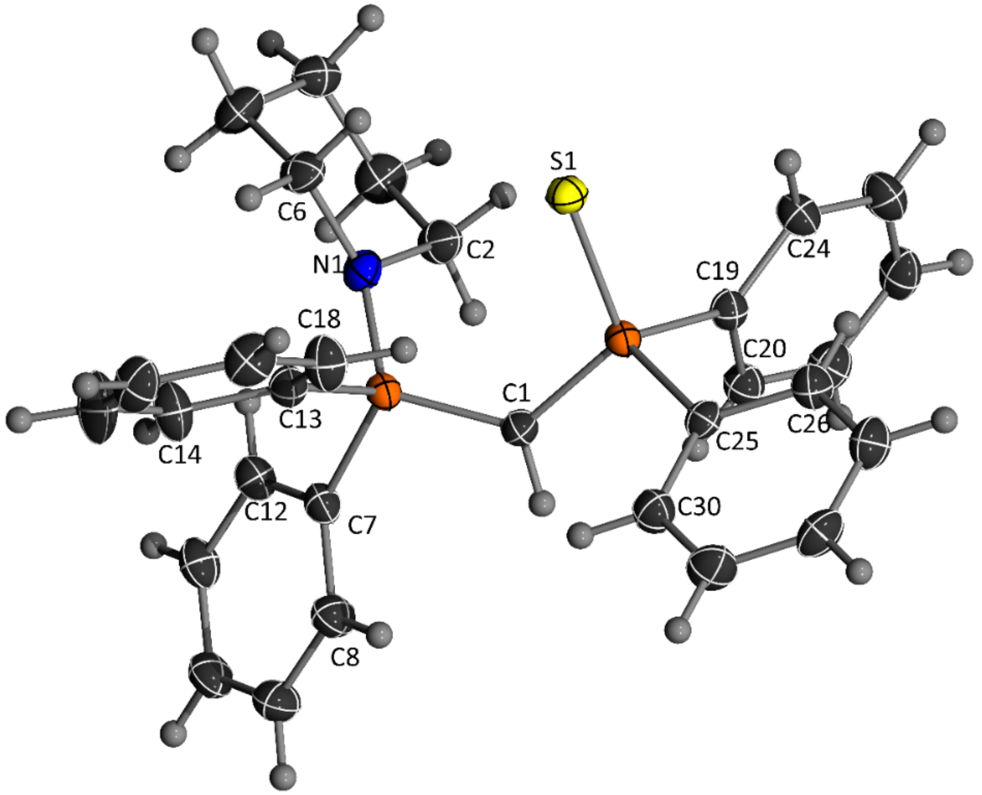

Figure s14 Molecular structure of **Y-H**. Thermal ellipsoids at 50% probability level. Selected bond lengths [Å] and angles [°]: P1–C1 1.694(2), C1–P2 1.723(2), P1–N1 1.6589(18), S1–P2 1.9743(7), P1–C1–P2 125.39(12).

Table s8 Atomic coordinates (x 10^4) and equivalent isotropic displacement parameters (Å^{2}x 10^3) for compound **Y-H**. U(eq) is defined as one third of the trace of the orthogonalized Uij tensor.

	x	y	z	U(eq)
S(1)	3549(1)	5869(1)	8130(1)	23(1)
P(1)	5176(1)	6659(1)	6444(1)	20(1)
N(1)	4945(2)	7557(1)	6965(1)	24(1)
C(1)	5826(2)	5777(1)	6932(1)	22(1)
P(2)	5285(1)	5385(1)	7804(1)	19(1)
C(2)	5977(2)	7776(2)	7576(1)	30(1)
C(3)	6142(2)	8751(2)	7670(2)	33(1)
C(4)	4806(2)	9213(1)	7775(1)	30(1)
C(5)	3762(2)	8946(2)	7140(2)	35(1)

C(6)	3632(2)	7972(2)	7077(1)	30(1)
C(7)	6347(2)	6933(1)	5703(1)	23(1)
C(8)	6782(2)	6282(2)	5199(1)	29(1)
C(9)	7562(2)	6506(2)	4582(1)	33(1)
C(10)	7935(2)	7371(2)	4465(1)	32(1)
C(11)	7505(2)	8020(2)	4960(1)	30(1)
C(12)	6716(2)	7802(1)	5577(1)	26(1)
C(13)	3610(2)	6514(1)	5875(1)	24(1)
C(14)	3360(3)	7024(2)	5200(1)	34(1)
C(15)	2165(3)	6935(2)	4754(2)	47(1)
C(16)	1224(2)	6300(2)	4948(1)	36(1)
C(17)	1461(2)	5794(2)	5614(2)	36(1)
C(18)	2649(2)	5884(2)	6060(1)	33(1)
C(19)	6602(2)	5559(1)	8577(1)	22(1)
C(20)	7955(2)	5659(1)	8394(1)	26(1)
C(21)	8963(2)	5737(2)	8983(1)	31(1)
C(22)	8633(2)	5716(2)	9754(1)	32(1)
C(23)	7289(3)	5640(2)	9947(1)	33(1)
C(24)	6283(2)	5567(1)	9357(1)	28(1)
C(25)	5219(2)	4188(1)	7729(1)	21(1)
C(26)	5252(2)	3663(1)	8395(1)	28(1)
C(27)	5109(2)	2759(2)	8337(1)	32(1)
C(28)	4930(2)	2370(1)	7616(1)	31(1)
C(29)	4906(2)	2881(2)	6948(1)	33(1)
C(30)	5050(2)	3783(1)	7003(1)	29(1)

Table s9 Anisotropic displacement parameters (Å^{2}x 10^3) for compound **Y-H**. The anisotropic displacement factor exponent takes the form: –2p^2[h^{2}a*^{2}U^{11} + ... + 2 h k a* b* U^{12}].

	**U^{11}**	**U^{22}**	**U^{33}**	**U^{23}**	**U^{13}**	**U^{12}**
S(1)	21(1)	25(1)	25(1)	0(1)	4(1)	1(1)
P(1)	19(1)	22(1)	18(1)	2(1)	0(1)	0(1)
N(1)	18(1)	28(1)	25(1)	1(1)	-1(1)	2(1)
C(1)	20(1)	24(1)	22(1)	2(1)	2(1)	3(1)
P(2)	19(1)	20(1)	18(1)	1(1)	0(1)	0(1)
C(2)	25(1)	36(1)	28(1)	-6(1)	-5(1)	4(1)

C(3)	30(1)	30(1)	38(1)	-4(1)	-1(1)	-5(1)
C(4)	32(1)	26(1)	32(1)	-3(1)	7(1)	-1(1)
C(5)	30(1)	29(1)	45(1)	-1(1)	0(1)	8(1)
C(6)	22(1)	36(1)	31(1)	-3(1)	0(1)	6(1)
C(7)	21(1)	29(1)	19(1)	4(1)	0(1)	-1(1)
C(8)	32(1)	28(1)	27(1)	1(1)	6(1)	-1(1)
C(9)	35(1)	35(1)	29(1)	0(1)	9(1)	3(1)
C(10)	29(1)	41(1)	26(1)	7(1)	7(1)	-1(1)
C(11)	30(1)	32(1)	27(1)	7(1)	1(1)	-6(1)
C(12)	27(1)	28(1)	23(1)	1(1)	0(1)	-2(1)
C(13)	25(1)	23(1)	23(1)	1(1)	-1(1)	0(1)
C(14)	37(1)	36(1)	29(1)	8(1)	-9(1)	-7(1)
C(15)	49(2)	46(2)	43(1)	13(1)	-21(1)	-6(1)
C(16)	33(1)	35(1)	38(1)	-5(1)	-15(1)	0(1)
C(17)	29(1)	32(1)	45(1)	-4(1)	-4(1)	-4(1)
C(18)	34(1)	31(1)	32(1)	2(1)	-8(1)	-7(1)
C(19)	24(1)	21(1)	22(1)	0(1)	-1(1)	-1(1)
C(20)	25(1)	28(1)	25(1)	0(1)	0(1)	-1(1)
C(21)	24(1)	31(1)	37(1)	0(1)	-4(1)	-1(1)
C(22)	33(1)	31(1)	31(1)	-2(1)	-11(1)	1(1)
C(23)	38(1)	38(1)	24(1)	-1(1)	-3(1)	-2(1)
C(24)	28(1)	32(1)	23(1)	0(1)	1(1)	-3(1)
C(25)	16(1)	22(1)	24(1)	1(1)	-1(1)	2(1)
C(26)	30(1)	26(1)	26(1)	2(1)	-1(1)	-1(1)
C(27)	35(1)	26(1)	36(1)	7(1)	-1(1)	-1(1)
C(28)	28(1)	21(1)	44(1)	-1(1)	0(1)	1(1)
C(29)	38(1)	29(1)	33(1)	-8(1)	-3(1)	0(1)
C(30)	31(1)	28(1)	27(1)	-1(1)	-2(1)	1(1)

3.5 Crystal Structure Determination of (Y-Li·THF)$_2$

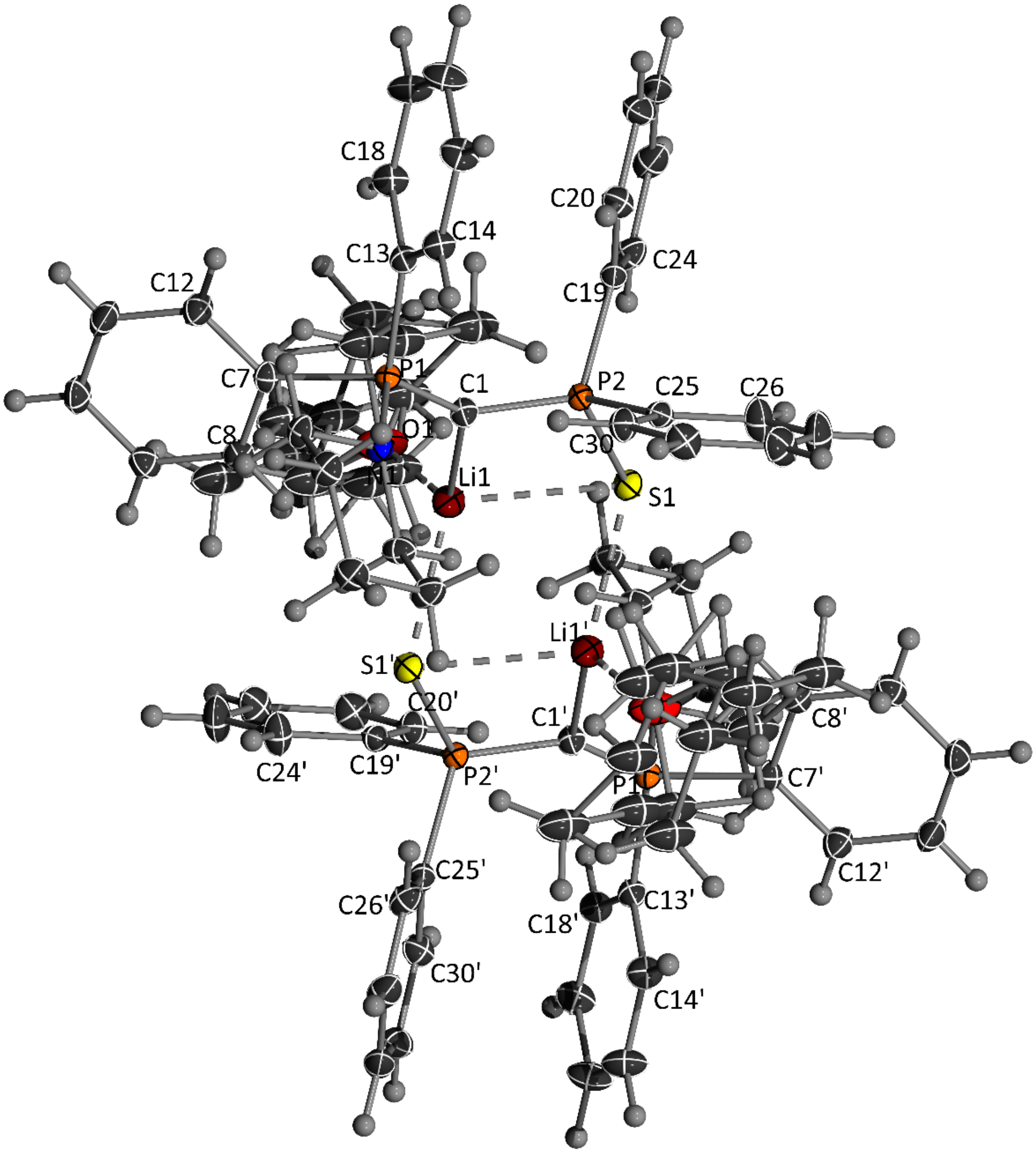

Figure s15 Molecular structure of **(Y-Li·THF)$_2$**. Thermal ellipsoids at 50% probability level. Selected bond lengths [Å] and angles [°]: P1-C1 1.6391(17), P2-C1 1.6671(18), N1-P1 1.6976(15), S1-P2 2.0332(6), Li1-C1 2.132(4), S1-Li1 2.531(3), S1-Li1' 2.487(3), P1-C1-P2 134.91(11). Symmetry operation: -x, -y, -z.

Table s10 Atomic coordinates (x 10^4) and equivalent isotropic displacement parameters (Å^{2}x 10^3) for compound **(Y-Li·THF)$_2$**. U(eq) is defined as one third of the trace of the orthogonalized Uij tensor.

	x	y	z	U(eq)
S(1)	3725(1)	824(1)	4021(1)	18(1)
N(1)	8679(2)	2031(1)	2989(1)	16(1)
P(1)	7081(1)	2750(1)	3467(1)	13(1)
C(1)	5814(2)	2110(2)	3787(2)	16(1)
P(2)	4907(1)	2040(1)	3059(1)	13(1)
C(2)	8893(2)	786(2)	3405(2)	19(1)
C(3)	10148(2)	316(2)	2566(2)	23(1)
C(5)	11140(2)	2041(2)	2029(2)	21(1)
C(4)	11427(2)	755(2)	2361(2)	23(1)
C(16)	6921(2)	6245(2)	712(2)	28(1)
C(30)	7264(2)	1799(2)	1208(2)	19(1)
C(29)	8000(2)	1553(2)	194(2)	24(1)
C(28)	7347(2)	1281(2)	-349(2)	27(1)
C(27)	5947(2)	1264(2)	117(2)	33(1)
C(26)	5215(2)	1495(2)	1134(2)	27(1)
C(25)	5870(2)	1764(2)	1693(2)	16(1)
C(24)	2324(2)	3491(2)	3222(2)	21(1)
C(23)	1446(2)	4507(2)	2898(2)	27(1)
C(22)	1954(2)	5392(2)	1959(2)	25(1)
C(21)	3343(2)	5272(2)	1352(2)	24(1)
C(20)	4223(2)	4261(2)	1673(2)	20(1)
C(19)	3719(2)	3354(2)	2610(2)	16(1)
C(18)	6090(2)	5038(2)	2635(2)	21(1)
C(17)	6000(2)	6086(2)	1802(2)	27(1)
C(15)	7939(2)	5360(2)	452(2)	24(1)
C(14)	8043(2)	4310(2)	1280(2)	19(1)
C(13)	7122(2)	4138(2)	2386(2)	16(1)
C(12)	7516(2)	3906(2)	4672(2)	22(1)
C(6)	9867(2)	2441(2)	2898(2)	18(1)
C(7)	7127(2)	2959(2)	4715(2)	16(1)
C(8)	6905(2)	2066(2)	5740(2)	18(1)

C(9)	7050(2)	2131(2)	6693(2)	21(1)
Li(11)	4748(3)	996(3)	5344(3)	22(1)
C(11)	7656(2)	3966(2)	5632(2)	26(1)
C(10)	7425(2)	3080(2)	6643(2)	24(1)
O(1A)	3505(2)	1704(1)	6473(1)	32(1)
C(5A)	3271(5)	1391(4)	7649(3)	44(1)
C(4A)	1743(6)	1969(4)	8031(5)	64(1)
C(3A)	1714(6)	3104(4)	6918(5)	64(1)
C(2A)	3134(6)	2911(4)	6144(4)	44(1)
O(1B)	3505(2)	1704(1)	6473(1)	32(1)
C(2B)	2782(9)	2854(7)	5998(7)	44(1)
C(3B)	1232(9)	2807(7)	6208(8)	64(1)
C(4B)	1097(9)	2081(7)	7390(8)	64(1)
C(5B)	2463(8)	1259(6)	7392(6)	44(1)
O(1C)	3505(2)	1704(1)	6473(1)	32(1)
C(2C)	4171(19)	2179(19)	6914(15)	44(1)
C(3C)	3090(30)	2540(20)	7930(20)	64(1)
C(4C)	2290(30)	1550(20)	8537(17)	64(1)
C(5C)	2730(30)	1033(17)	7585(14)	44(1)

Table s11 Anisotropic displacement parameters (Å^{2}x 10^3) for compound **(Y-Li·THF)$_2$**. The anisotropic displacement factor exponent takes the form: $-2p^2$[$h^2a^{*2}U^{11}$ + ... + 2 h k a* b* U^{12}].

	U^{11}	U^{22}	U^{33}	U^{23}	U^{1}	U^{12}
S(1)	18(1)	18(1)	20(1)	-2(1)	-7(1)	-8(1)
N(1)	13(1)	16(1)	18(1)	-5(1)	-4(1)	-4(1)
P(1)	13(1)	14(1)	12(1)	-4(1)	-3(1)	-4(1)
C(1)	14(1)	18(1)	14(1)	-3(1)	-3(1)	-6(1)
P(2)	12(1)	14(1)	13(1)	-4(1)	-3(1)	-4(1)
C(2)	18(1)	15(1)	23(1)	-4(1)	-6(1)	-4(1)
C(3)	18(1)	20(1)	32(1)	-12(1)	-7(1)	-1(1)
C(5)	14(1)	24(1)	24(1)	-10(1)	-3(1)	-5(1)
C(4)	15(1)	25(1)	30(1)	-12(1)	-6(1)	-1(1)
C(16)	27(1)	20(1)	25(1)	3(1)	-6(1)	-4(1)
C(30)	20(1)	22(1)	17(1)	-7(1)	-5(1)	-5(1)

C(29)	20(1)	29(1)	19(1)	-9(1)	-2(1)	-4(1)
C(28)	29(1)	33(1)	18(1)	-14(1)	-6(1)	1(1)
C(27)	29(1)	49(1)	33(1)	-26(1)	-13(1)	0(1)
C(26)	18(1)	41(1)	28(1)	-20(1)	-8(1)	-1(1)
C(25)	18(1)	15(1)	16(1)	-5(1)	-6(1)	0(1)
C(24)	17(1)	21(1)	29(1)	-12(1)	-5(1)	-4(1)
C(23)	17(1)	26(1)	44(1)	-20(1)	-10(1)	1(1)
C(22)	27(1)	21(1)	36(1)	-15(1)	-19(1)	6(1)
C(21)	32(1)	19(1)	21(1)	-5(1)	-12(1)	0(1)
C(20)	20(1)	21(1)	17(1)	-6(1)	-5(1)	-1(1)
C(19)	16(1)	16(1)	18(1)	-7(1)	-7(1)	-2(1)
C(18)	19(1)	21(1)	18(1)	-5(1)	-1(1)	-4(1)
C(17)	23(1)	18(1)	30(1)	-4(1)	-4(1)	0(1)
C(15)	22(1)	25(1)	17(1)	-2(1)	-2(1)	-7(1)
C(14)	17(1)	20(1)	17(1)	-5(1)	-3(1)	-4(1)
C(13)	16(1)	16(1)	15(1)	-3(1)	-5(1)	-6(1)
C(12)	26(1)	21(1)	19(1)	-6(1)	-6(1)	-9(1)
C(6)	14(1)	22(1)	21(1)	-8(1)	-5(1)	-6(1)
C(7)	14(1)	18(1)	16(1)	-8(1)	-4(1)	-3(1)
C(8)	19(1)	18(1)	18(1)	-5(1)	-6(1)	-4(1)
C(9)	23(1)	22(1)	17(1)	-6(1)	-7(1)	-3(1)
Li(11)	25(2)	21(2)	18(2)	-5(1)	-4(1)	-7(1)
C(11)	32(1)	24(1)	28(1)	-12(1)	-10(1)	-8(1)
C(10)	27(1)	28(1)	21(1)	-13(1)	-9(1)	-2(1)
O(1A)	45(1)	20(1)	26(1)	-7(1)	-9(1)	-1(1)
C(5A)	62(2)	26(1)	27(1)	-15(1)	-1(1)	8(1)
C(4A)	54(2)	30(1)	64(2)	-9(1)	13(1)	3(1)
C(3A)	54(2)	30(1)	64(2)	-9(1)	13(1)	3(1)
C(2A)	62(2)	26(1)	27(1)	-15(1)	-1(1)	8(1)
O(1B)	45(1)	20(1)	26(1)	-7(1)	-9(1)	-1(1)
C(2B)	62(2)	26(1)	27(1)	-15(1)	-1(1)	8(1)
C(3B)	54(2)	30(1)	64(2)	-9(1)	13(1)	3(1)
C(4B)	54(2)	30(1)	64(2)	-9(1)	13(1)	3(1)
C(5B)	62(2)	26(1)	27(1)	-15(1)	-1(1)	8(1)
O(1C)	45(1)	20(1)	26(1)	-7(1)	-9(1)	-1(1)
C(2C)	62(2)	26(1)	27(1)	-15(1)	-1(1)	8(1)

C(3C)	54(2)	30(1)	64(2)	-9(1)	13(1)	3(1)
C(4C)	54(2)	30(1)	64(2)	-9(1)	13(1)	3(1)
C(5C)	62(2)	26(1)	27(1)	-15(1)	-1(1)	8(1)

3.6 Crystal Structure Determination of [Y_2Li][Li(12-C-4)$_2$]

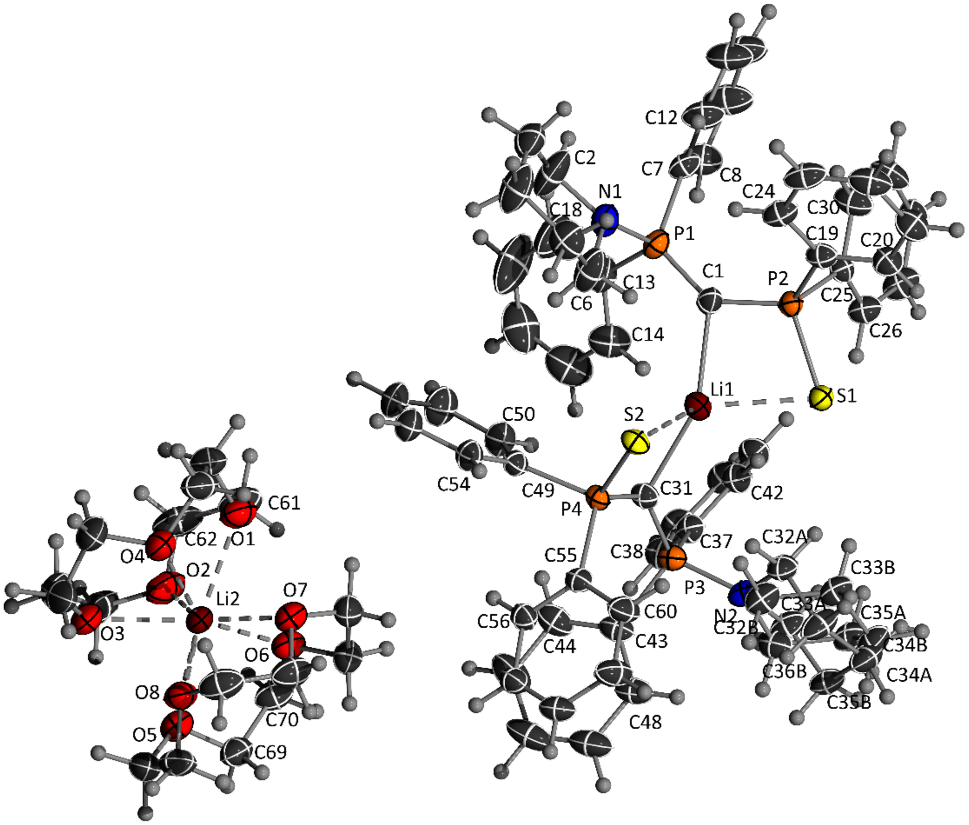

Figure s16 Molecular structure of **[Y_2Li][Li(12-C-4)$_2$]**. Thermal ellipsoids at 50% probability level. Selected bond lengths [Å] and angles [°]: P1-C1 1.639(2), P2-C1 1.658(2), N1-P1 1.725(2), S1-P2 2.0141(7), C1-Li1 2.215(4), S1-Li1 2.499(3), P1-C1-P2 135.95(13), C1-Li1-C31 139.11(19).

Table s12 Atomic coordinates (x 10^4) and equivalent isotropic displacement parameters (Å^{2}x 10^3) for compound **[Y_2Li][Li(12-C-4)$_2$]**. U(eq) is defined as one third of the trace of the orthogonalized Uij tensor.

	x	y	z	U(eq)
S(1)	1192(1)	7327(1)	3563(1)	29(1)
P(1)	2740(1)	8569(1)	4701(1)	31(1)

O(1)	7585(1)	5934(2)	3812(1)	52(1)
C(1)	2252(1)	8146(2)	4271(1)	33(1)
Li(1)	2529(2)	7240(3)	3676(1)	34(1)
S(2)	3379(1)	7933(1)	3061(1)	29(1)
N(2)	2153(1)	4539(1)	3057(1)	33(1)
P(2)	1400(1)	8250(1)	4091(1)	26(1)
O(2)	8335(1)	4173(2)	4013(1)	62(1)
N(1A)	3348(1)	9493(2)	4557(1)	39(1)
C(2A)	3883(2)	9863(3)	4883(1)	42(1)
C(3A)	4109(3)	10943(6)	4743(3)	45(1)
C(4A)	4373(3)	10968(8)	4278(2)	61(2)
C(5A)	3891(5)	10394(8)	3955(2)	49(1)
C(6A)	3648(2)	9373(4)	4108(1)	40(1)
N(1B)	3348(1)	9493(2)	4557(1)	39(1)
C(2B)	3664(7)	10279(10)	4894(3)	42(1)
C(3B)	4256(13)	10940(20)	4762(8)	45(1)
C(4B)	4533(11)	10860(30)	4311(8)	61(2)
C(5B)	4007(18)	10530(30)	3968(8)	49(1)
C(6B)	3513(7)	9773(10)	4117(4)	40(1)
Li(2)	8146(2)	4937(3)	3279(1)	33(1)
P(3)	2866(1)	4703(1)	3423(1)	28(1)
O(3)	9353(1)	4880(2)	3456(1)	62(1)
P(4)	3750(1)	6536(1)	3228(1)	25(1)
O(4)	8596(1)	6642(2)	3246(1)	51(1)
O(5)	8427(1)	3150(1)	3118(1)	49(1)
O(6)	7134(1)	4031(1)	3355(1)	41(1)
O(7)	7250(1)	5714(1)	2816(1)	41(1)
C(7)	2310(1)	9152(2)	5167(1)	34(1)
O(8)	8527(1)	4857(1)	2585(1)	46(1)
C(8)	2024(2)	8542(2)	5507(1)	46(1)
C(9)	1651(2)	8992(2)	5850(1)	62(1)
C(10)	1466(2)	10011(2)	5834(1)	55(1)
C(11)	1736(2)	10623(2)	5491(1)	58(1)
C(12)	2113(2)	10178(2)	5150(1)	48(1)
C(13)	3263(2)	7485(3)	4965(1)	36(1)
C(14)	3200(2)	6526(3)	4771(1)	42(1)

C(15)	3602(2)	5718(3)	4948(1)	50(1)
C(16)	4068(2)	5870(3)	5318(1)	58(1)
C(17)	4130(2)	6829(4)	5511(1)	78(2)
C(18)	3728(2)	7637(3)	5335(1)	61(1)
C(13B)	3292(7)	7727(9)	4975(5)	36(1)
C(18B)	3602(9)	7960(10)	5394(5)	61(1)
C(17B)	3998(9)	7227(13)	5632(5)	78(2)
C(16B)	4084(7)	6262(12)	5451(5)	58(1)
C(15B)	3773(8)	6029(10)	5032(5)	50(1)
C(14B)	3377(8)	6761(10)	4794(4)	42(1)
C(19)	1106(1)	9532(2)	3913(1)	31(1)
C(20)	428(1)	9677(2)	3701(1)	37(1)
C(21)	200(1)	10639(2)	3567(1)	44(1)
C(22)	646(2)	11474(2)	3647(1)	47(1)
C(23)	1314(2)	11339(2)	3852(1)	45(1)
C(24)	1548(1)	10371(2)	3983(1)	38(1)
C(25)	728(1)	7968(2)	4511(1)	28(1)
C(26)	453(1)	6992(2)	4550(1)	36(1)
C(27)	-34(1)	6763(2)	4877(1)	44(1)
C(28)	-242(1)	7500(2)	5175(1)	39(1)
C(29)	35(1)	8471(2)	5148(1)	46(1)
C(30)	521(1)	8704(2)	4819(1)	46(1)
C(31)	3152(1)	5867(2)	3514(1)	29(1)
C(32A)	1789(2)	5416(2)	2840(1)	37(1)
C(33A)	1500(3)	5112(3)	2375(2)	48(1)
C(34A)	971(3)	4232(4)	2404(2)	45(2)
C(35A)	1333(2)	3345(2)	2650(1)	39(1)
C(36A)	1662(3)	3670(4)	3100(2)	40(1)
C(32B)	2053(5)	5190(7)	2663(3)	38(2)
C(33B)	1259(5)	5333(7)	2575(3)	39(2)
C(34B)	854(8)	4331(10)	2521(4)	42(3)
C(35B)	1013(4)	3654(6)	2920(3)	39(2)
C(36B)	1824(6)	3515(8)	2998(5)	39(3)
C(37)	2526(1)	4179(2)	3940(1)	31(1)
C(38)	2792(1)	3311(2)	4155(1)	37(1)
C(39)	2509(1)	2990(2)	4557(1)	42(1)

C(40)	1949(1)	3512(2)	4738(1)	41(1)
C(41)	1677(1)	4375(2)	4525(1)	40(1)
C(42)	1965(1)	4715(2)	4130(1)	35(1)
C(43)	3518(1)	3775(2)	3233(1)	35(1)
C(44)	4142(1)	3582(2)	3488(1)	43(1)
C(45)	4660(2)	2921(2)	3330(1)	54(1)
C(46)	4552(2)	2471(2)	2909(1)	59(1)
C(47)	3947(2)	2679(2)	2650(1)	54(1)
C(48)	3427(2)	3319(2)	2810(1)	44(1)
C(49)	4577(1)	6761(2)	3569(1)	28(1)
C(50)	4631(1)	6396(2)	4004(1)	41(1)
C(51)	5224(2)	6650(2)	4284(1)	53(1)
C(52)	5774(1)	7225(2)	4123(1)	49(1)
C(53)	5733(1)	7579(2)	3686(1)	43(1)
C(54)	5131(1)	7360(2)	3412(1)	35(1)
C(55)	4079(1)	5928(1)	2722(1)	28(1)
C(56)	4684(1)	5299(2)	2729(1)	31(1)
C(57)	4895(1)	4812(2)	2341(1)	35(1)
C(58)	4507(1)	4947(2)	1941(1)	40(1)
C(59)	3897(2)	5555(2)	1932(1)	48(1)
C(60)	3687(1)	6038(2)	2319(1)	40(1)
C(61)	7459(2)	5323(3)	4208(1)	61(1)
C(62)	8110(2)	4770(3)	4369(1)	68(1)
C(63)	9079(2)	3851(3)	4049(1)	86(1)
C(64)	9567(2)	4642(3)	3899(1)	80(1)

Table s13 Anisotropic displacement parameters (Å^{2}x 10^3) for compound **[Y_2Li][Li(12-C-4)$_2$]**. The anisotropic displacement factor exponent takes the form: $-2p^2[h^2a^{*2}U^{11} + ... + 2 h k a^* b^* U^{12}]$.

	U^{11}	U^{22}	U^{33}	U^{23}	U^{13}	U^{12}
S(1)	28(1)	31(1)	30(1)	-5(1)	2(1)	-4(1)
P(1)	32(1)	36(1)	26(1)	0(1)	-1(1)	-7(1)
O(1)	59(1)	54(1)	42(1)	-15(1)	1(1)	-7(1)
C(1)	27(1)	44(1)	30(1)	-6(1)	3(1)	-5(1)

Li(1)	31(2)	36(2)	36(2)	-3(1)	6(1)	1(1)
S(2)	32(1)	25(1)	31(1)	3(1)	4(1)	3(1)
N(2)	43(1)	27(1)	29(1)	1(1)	4(1)	-9(1)
P(2)	25(1)	28(1)	27(1)	-2(1)	4(1)	-4(1)
O(2)	76(1)	62(1)	47(1)	-12(1)	-19(1)	0(1)
N(1A)	34(1)	51(1)	33(1)	1(1)	-1(1)	-7(1)
C(2A)	35(2)	53(2)	39(1)	-5(2)	-6(1)	-9(2)
C(3A)	28(3)	51(1)	57(2)	-4(1)	-6(2)	-13(2)
C(4A)	36(3)	81(3)	64(2)	19(2)	-9(2)	-29(3)
C(5A)	47(4)	52(3)	50(2)	8(2)	12(2)	-19(2)
C(6A)	36(2)	49(3)	36(1)	-3(2)	5(1)	-7(2)
N(1B)	34(1)	51(1)	33(1)	1(1)	-1(1)	-7(1)
C(2B)	35(2)	53(2)	39(1)	-5(2)	-6(1)	-9(2)
C(3B)	28(3)	51(1)	57(2)	-4(1)	-6(2)	-13(2)
C(4B)	36(3)	81(3)	64(2)	19(2)	-9(2)	-29(3)
C(5B)	47(4)	52(3)	50(2)	8(2)	12(2)	-19(2)
C(6B)	36(2)	49(3)	36(1)	-3(2)	5(1)	-7(2)
Li(2)	29(2)	34(2)	36(2)	-9(1)	-2(1)	-3(1)
P(3)	33(1)	24(1)	27(1)	0(1)	8(1)	-3(1)
O(3)	43(1)	60(1)	84(1)	-33(1)	-9(1)	-5(1)
P(4)	25(1)	22(1)	26(1)	1(1)	5(1)	0(1)
O(4)	40(1)	58(1)	55(1)	-20(1)	1(1)	-9(1)
O(5)	45(1)	51(1)	48(1)	-14(1)	-7(1)	-3(1)
O(6)	47(1)	40(1)	36(1)	-5(1)	-1(1)	-2(1)
O(7)	42(1)	40(1)	40(1)	-7(1)	-3(1)	-4(1)
C(7)	43(1)	34(1)	24(1)	-2(1)	0(1)	-7(1)
O(8)	47(1)	48(1)	44(1)	-15(1)	9(1)	-10(1)
C(8)	61(2)	43(1)	34(1)	5(1)	9(1)	0(1)
C(9)	96(2)	52(2)	40(1)	-7(1)	28(1)	-16(2)
C(10)	71(2)	52(2)	44(1)	-15(1)	16(1)	-6(1)
C(11)	85(2)	37(1)	53(2)	-12(1)	17(1)	-6(1)
C(12)	72(2)	38(1)	34(1)	-2(1)	7(1)	-5(1)
C(13)	36(1)	35(2)	37(1)	4(1)	-2(1)	-10(1)
C(14)	55(2)	32(2)	39(1)	1(1)	4(1)	-11(1)
C(15)	72(2)	32(2)	47(2)	6(1)	12(2)	-4(2)
C(16)	60(2)	44(3)	68(3)	17(2)	-1(2)	11(2)

C(17)	87(3)	43(3)	98(3)	16(3)	-51(3)	-6(2)
C(18)	66(2)	45(2)	70(2)	-7(2)	-32(2)	11(2)
C(13B)	36(1)	35(2)	37(1)	4(1)	-2(1)	-10(1)
C(18B)	66(2)	45(2)	70(2)	-7(2)	-32(2)	11(2)
C(17B)	87(3)	43(3)	98(3)	16(3)	-51(3)	-6(2)
C(16B)	60(2)	44(3)	68(3)	17(2)	-1(2)	11(2)
C(15B)	72(2)	32(2)	47(2)	6(1)	12(2)	-4(2)
C(14B)	55(2)	32(2)	39(1)	1(1)	4(1)	-11(1)
C(19)	38(1)	30(1)	27(1)	-2(1)	8(1)	-4(1)
C(20)	38(1)	32(1)	41(1)	2(1)	5(1)	-2(1)
C(21)	48(1)	40(1)	45(1)	6(1)	7(1)	6(1)
C(22)	74(2)	30(1)	38(1)	4(1)	16(1)	4(1)
C(23)	72(2)	33(1)	33(1)	-2(1)	11(1)	-15(1)
C(24)	50(1)	36(1)	27(1)	-1(1)	5(1)	-12(1)
C(25)	26(1)	29(1)	29(1)	2(1)	3(1)	1(1)
C(26)	46(1)	31(1)	31(1)	-1(1)	6(1)	-6(1)
C(27)	57(1)	42(1)	34(1)	2(1)	9(1)	-18(1)
C(28)	35(1)	51(1)	31(1)	8(1)	7(1)	-2(1)
C(29)	56(1)	41(1)	44(1)	2(1)	21(1)	10(1)
C(30)	58(1)	33(1)	48(1)	-1(1)	22(1)	-2(1)
C(31)	32(1)	27(1)	30(1)	-1(1)	9(1)	-2(1)
C(32A)	40(2)	26(1)	44(2)	-6(1)	-2(1)	-2(1)
C(33A)	64(2)	31(2)	47(2)	2(2)	-16(2)	-9(2)
C(34A)	51(3)	36(2)	46(3)	-7(2)	-11(2)	-7(2)
C(35A)	49(2)	30(1)	38(2)	-6(1)	2(1)	-11(1)
C(36A)	49(2)	35(2)	34(2)	0(2)	3(2)	-17(2)
C(32B)	48(4)	37(4)	30(4)	7(3)	2(3)	-11(3)
C(33B)	47(4)	37(4)	32(4)	2(3)	2(3)	-11(3)
C(34B)	47(5)	44(5)	36(6)	-4(4)	1(5)	-16(4)
C(35B)	45(4)	32(4)	40(4)	-2(3)	7(3)	-13(3)
C(36B)	44(4)	29(4)	44(7)	-2(4)	16(4)	-8(3)
C(37)	34(1)	29(1)	29(1)	0(1)	6(1)	-7(1)
C(38)	41(1)	31(1)	38(1)	4(1)	8(1)	-5(1)
C(39)	48(1)	40(1)	38(1)	11(1)	3(1)	-10(1)
C(40)	46(1)	51(1)	28(1)	2(1)	7(1)	-14(1)
C(41)	40(1)	49(1)	32(1)	-2(1)	11(1)	-9(1)

C(42)	37(1)	36(1)	33(1)	0(1)	7(1)	-5(1)
C(43)	46(1)	23(1)	36(1)	2(1)	16(1)	-1(1)
C(44)	50(1)	40(1)	39(1)	7(1)	16(1)	6(1)
C(45)	57(2)	47(1)	59(2)	20(1)	23(1)	16(1)
C(46)	82(2)	31(1)	67(2)	3(1)	41(2)	12(1)
C(47)	76(2)	34(1)	54(1)	-10(1)	27(1)	-3(1)
C(48)	61(1)	30(1)	43(1)	-7(1)	18(1)	-6(1)
C(49)	28(1)	26(1)	32(1)	-1(1)	3(1)	2(1)
C(50)	41(1)	45(1)	36(1)	7(1)	-1(1)	-3(1)
C(51)	54(2)	66(2)	37(1)	8(1)	-9(1)	-2(1)
C(52)	39(1)	58(2)	48(1)	-6(1)	-12(1)	-1(1)
C(53)	33(1)	45(1)	50(1)	-3(1)	0(1)	-6(1)
C(54)	34(1)	37(1)	36(1)	2(1)	2(1)	-3(1)
C(55)	30(1)	23(1)	31(1)	0(1)	7(1)	-2(1)
C(56)	28(1)	32(1)	34(1)	-1(1)	6(1)	0(1)
C(57)	33(1)	32(1)	42(1)	-5(1)	10(1)	0(1)
C(58)	53(1)	31(1)	36(1)	-6(1)	11(1)	1(1)
C(59)	65(2)	44(1)	32(1)	-8(1)	-6(1)	13(1)
C(60)	48(1)	37(1)	37(1)	-6(1)	-2(1)	13(1)
C(61)	79(2)	69(2)	36(1)	-10(1)	8(1)	-14(2)
C(62)	106(3)	60(2)	38(1)	-9(1)	-6(2)	-11(2)
C(63)	100(3)	57(2)	96(3)	-25(2)	-61(2)	21(2)
C(64)	59(2)	90(2)	89(2)	-41(2)	-35(2)	22(2)

3.7 Crystal Structure Determination (Y-K)$_6$

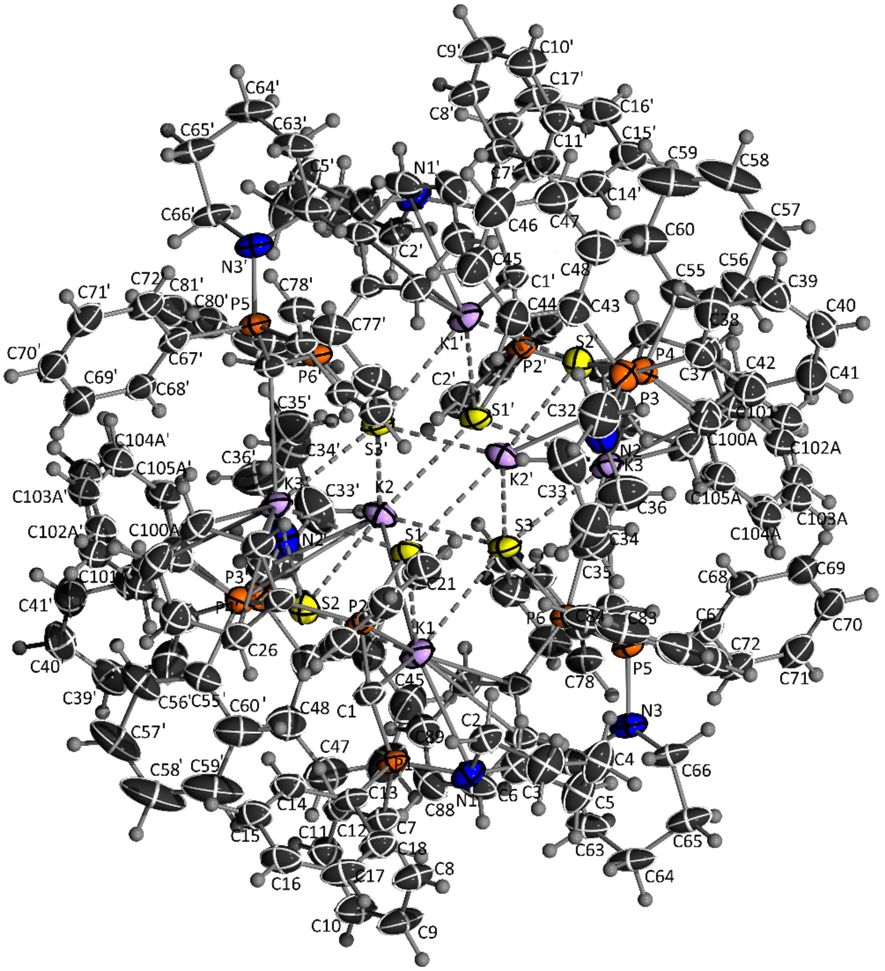

Figure s17 Molecular structure of **(Y-K)$_6$**. Thermal ellipsoids at 50% probability level. Selected bond lengths [Å] and angles [°]: P1-C1 1.585(2), P2-C1 1.674(3), N1-P1 1.708(2), S1-P2 2.0337(8), C1-K1 2.872(3), S1-K1 3.1413(9), P1-C1-P2 138.93(17). Symmetry operations: -x, y+1/2, -z+1/2; -x, -y, -z and x, -y-1/2, z-1/2.

Table s14 Atomic coordinates (x 10^4) and equivalent isotropic displacement parameters (Å^{2}x 10^3) for compound **(Y-K)$_6$**. U(eq) is defined as one third of the trace of the orthogonalized Uij tensor.

	x	y	z	U(eq)
N(1)	2396(1)	8208(1)	5283(1)	40(1)
K(1)	3399(1)	6389(1)	4683(1)	43(1)
S(1)	4905(1)	6985(1)	5288(1)	38(1)

P(1)	2743(1)	8306(1)	4842(1)	35(1)
C(1)	3496(1)	7896(1)	4819(1)	39(1)
K(2)	4584(1)	4509(1)	4236(1)	39(1)
N(2)	4144(2)	3525(1)	3031(1)	58(1)
S(2)	4115(1)	6113(1)	3959(1)	45(1)
P(2)	4434(1)	7924(1)	5046(1)	34(1)
C(2)	2905(2)	8496(2)	5676(1)	44(1)
C(3)	2479(2)	8567(2)	6017(1)	60(1)
P(3)	3769(1)	4370(1)	2974(1)	45(1)
S(3)	4014(1)	4780(1)	5101(1)	39(1)
K(3)	4305(1)	3279(1)	5550(1)	38(1)
N(3)	1072(1)	3586(1)	4722(1)	39(1)
P(4)	4230(1)	5763(1)	3392(1)	41(1)
C(4)	2158(2)	7859(2)	6112(1)	59(1)
P(5)	1968(1)	3270(1)	4926(1)	33(1)
C(5)	1706(2)	7510(2)	5705(1)	62(1)
C(6)	2144(2)	7495(1)	5367(1)	45(1)
P(6)	2938(1)	4582(1)	5158(1)	32(1)
C(7)	1936(1)	8033(1)	4410(1)	39(1)
C(8)	1179(2)	8140(2)	4422(1)	55(1)
C(9)	592(2)	7969(2)	4073(1)	66(1)
C(10)	748(2)	7713(2)	3707(1)	59(1)
C(11)	1493(2)	7613(2)	3689(1)	49(1)
C(12)	2081(2)	7763(1)	4041(1)	41(1)
C(13)	2680(1)	9269(1)	4783(1)	40(1)
C(14)	3087(2)	9593(2)	4520(1)	48(1)
C(15)	3058(2)	10324(2)	4456(1)	53(1)
C(16)	2583(2)	10736(2)	4635(1)	57(1)
C(18)	2200(2)	9695(2)	4956(1)	53(1)
C(17)	2174(2)	10424(2)	4892(1)	59(1)
C(19)	4764(1)	8608(1)	5455(1)	36(1)
C(20)	5109(2)	8448(1)	5876(1)	45(1)
C(21)	5368(2)	8987(2)	6170(1)	52(1)
C(23)	4915(2)	9857(1)	5628(1)	46(1)
C(22)	5269(2)	9692(2)	6046(1)	50(1)
C(24)	4670(2)	9321(1)	5335(1)	41(1)

C(25)	4988(1)	8176(1)	4664(1)	36(1)
C(26)	4656(2)	8230(1)	4212(1)	45(1)
C(28)	5842(2)	8487(2)	4084(1)	57(1)
C(27)	5057(2)	8384(2)	3942(1)	50(1)
C(29)	6198(2)	8447(2)	4512(1)	50(1)
C(30)	5776(2)	8295(1)	4803(1)	42(1)
C(31)	4280(2)	4889(2)	3337(1)	47(1)
C(32)	3729(3)	2985(2)	2742(1)	76(1)
C(33)	4001(3)	2267(2)	2872(1)	81(1)
C(34)	4835(3)	2188(2)	2954(2)	79(1)
C(35)	5259(2)	2758(2)	3242(2)	80(1)
C(36)	4942(2)	3476(2)	3111(2)	85(1)
C(37)	3714(2)	4579(2)	2423(1)	44(1)
C(38)	3119(2)	4408(2)	2075(1)	73(1)
C(39)	3120(2)	4613(2)	1667(1)	61(1)
C(40)	3777(2)	4892(2)	1583(1)	56(1)
C(41)	4387(2)	5060(2)	1920(1)	75(1)
C(42)	4379(2)	4866(2)	2335(1)	61(1)
C(43)	2776(2)	4238(2)	2985(1)	56(1)
C(44)	2590(2)	3767(2)	3264(1)	62(1)
C(45)	1840(2)	3694(3)	3292(1)	76(1)
C(46)	1282(2)	4124(2)	3075(1)	77(1)
C(47)	1457(2)	4647(2)	2810(1)	75(1)
C(48)	2206(2)	4721(2)	2772(1)	63(1)
C(100)	5136(2)	6150(3)	3328(2)	48(1)
C(101)	5232(3)	6882(3)	3343(2)	48(1)
C(102)	5913(3)	7178(3)	3300(2)	49(1)
C(103)	6498(2)	6741(3)	3242(1)	49(1)
C(104)	6402(2)	6009(3)	3227(2)	49(1)
C(105)	5721(3)	5713(2)	3270(2)	49(1)
C(106)	5064(4)	6253(5)	3330(3)	48(1)
C(107)	5049(4)	6988(4)	3264(2)	48(1)
C(108)	5684(4)	7369(4)	3226(2)	49(1)
C(109)	6367(4)	7017(4)	3271(2)	49(1)
C(110)	6414(4)	6298(5)	3341(2)	49(1)
C(111)	5766(4)	5916(4)	3376(2)	49(1)

C(55)	3484(2)	6213(2)	2991(1)	53(1)
C(56)	3597(2)	6347(2)	2587(1)	64(1)
C(58)	2283(3)	6734(3)	2350(2)	114(2)
C(59)	2184(3)	6615(3)	2749(2)	101(2)
C(60)	2779(2)	6362(2)	3071(1)	76(1)
C(61)	2694(1)	3735(1)	5178(1)	37(1)
C(62)	856(1)	3946(1)	4308(1)	44(1)
C(63)	-6(2)	3897(2)	4136(1)	52(1)
C(64)	-410(2)	4235(2)	4445(1)	59(1)
C(65)	-142(2)	3911(2)	4884(1)	55(1)
C(66)	725(1)	3932(2)	5034(1)	45(1)
C(67)	1757(1)	2571(1)	5275(1)	38(1)
C(68)	2244(2)	2463(2)	5670(1)	47(1)
C(69)	2108(2)	1932(2)	5943(1)	60(1)
C(70)	1470(2)	1504(2)	5815(1)	60(1)
C(71)	976(2)	1608(2)	5424(1)	57(1)
C(72)	1108(2)	2138(1)	5150(1)	47(1)
C(73)	2127(1)	2773(1)	4468(1)	36(1)
C(74)	2857(2)	2791(1)	4400(1)	40(1)
C(75)	3030(2)	2405(2)	4073(1)	50(1)
C(76)	2478(2)	2001(2)	3807(1)	58(1)
C(77)	1745(2)	1980(2)	3865(1)	56(1)
C(78)	1568(2)	2366(1)	4191(1)	44(1)
C(79)	2850(2)	5078(1)	5632(1)	42(1)
C(80)	2365(2)	4838(2)	5880(1)	52(1)
C(81)	2294(2)	5194(2)	6233(1)	73(1)
C(82)	2700(3)	5814(2)	6351(1)	81(1)
C(83)	3177(2)	6061(2)	6120(1)	74(1)
C(84)	3257(2)	5708(2)	5760(1)	56(1)
C(85)	2350(1)	5098(1)	4716(1)	35(1)
C(86)	1860(1)	5647(1)	4764(1)	39(1)
C(87)	1460(2)	6018(2)	4402(1)	50(1)
C(88)	1547(2)	5843(2)	4003(1)	58(1)
C(89)	2025(2)	5303(2)	3953(1)	54(1)
C(90)	2425(2)	4932(2)	4306(1)	44(1)

C(57)	2998(3)	6612(2)	2266(1)	94(2)

Table s15 Anisotropic displacement parameters (Å^{2}x 10^3) for compound **(Y-K)$_6$**. The anisotropic displacement factor exponent takes the form: –2p^2[h^{2}a*^{2}U^{11} + ... + 2 h k a* b* U^{12}].

	U^{11}	U^{22}	U^{33}	U^{23}	U^{13}	U^{12}
N(1)	32(1)	35(1)	56(1)	-8(1)	14(1)	-4(1)
K(1)	32(1)	30(1)	66(1)	-2(1)	14(1)	-2(1)
S(1)	30(1)	22(1)	61(1)	6(1)	10(1)	5(1)
P(1)	25(1)	27(1)	53(1)	-1(1)	10(1)	5(1)
C(1)	30(1)	30(1)	58(2)	1(1)	10(1)	7(1)
K(2)	41(1)	30(1)	42(1)	1(1)	1(1)	2(1)
N(2)	74(2)	49(1)	52(2)	-4(1)	16(1)	-8(1)
S(2)	48(1)	43(1)	45(1)	0(1)	14(1)	0(1)
P(2)	25(1)	22(1)	55(1)	3(1)	12(1)	4(1)
C(2)	36(1)	41(1)	56(2)	-10(1)	11(1)	-3(1)
C(3)	65(2)	56(2)	66(2)	-20(2)	26(2)	-14(2)
P(3)	45(1)	51(1)	38(1)	-3(1)	8(1)	-2(1)
S(3)	22(1)	33(1)	59(1)	1(1)	6(1)	0(1)
K(3)	28(1)	31(1)	57(1)	1(1)	12(1)	5(1)
N(3)	23(1)	34(1)	56(1)	-8(1)	3(1)	4(1)
P(4)	39(1)	42(1)	39(1)	3(1)	5(1)	4(1)
C(4)	68(2)	59(2)	61(2)	-17(2)	34(2)	-15(2)
P(5)	20(1)	28(1)	50(1)	-2(1)	7(1)	2(1)
C(5)	63(2)	55(2)	75(2)	-14(2)	33(2)	-21(2)
C(6)	47(2)	34(1)	55(2)	-6(1)	13(1)	-7(1)
P(6)	20(1)	30(1)	44(1)	-3(1)	4(1)	2(1)
C(7)	30(1)	34(1)	52(2)	-2(1)	9(1)	6(1)
C(8)	31(1)	70(2)	62(2)	-16(2)	9(1)	10(1)
C(9)	28(1)	93(3)	73(2)	-20(2)	3(1)	10(2)
C(10)	43(2)	70(2)	59(2)	-10(2)	2(1)	6(2)
C(11)	48(2)	47(2)	50(2)	-1(1)	11(1)	6(1)
C(12)	35(1)	36(1)	54(2)	2(1)	12(1)	6(1)
C(13)	26(1)	34(1)	59(2)	-6(1)	8(1)	7(1)
C(14)	45(2)	36(1)	64(2)	6(1)	16(1)	14(1)
C(15)	44(2)	34(1)	80(2)	6(1)	14(2)	7(1)

C(16)	50(2)	33(1)	83(2)	5(1)	8(2)	13(1)
C(18)	42(2)	44(2)	75(2)	1(1)	17(1)	14(1)
C(17)	42(2)	41(2)	92(2)	-16(2)	12(2)	15(1)
C(19)	27(1)	26(1)	60(2)	-1(1)	16(1)	5(1)
C(20)	49(2)	33(1)	56(2)	4(1)	20(1)	10(1)
C(21)	61(2)	41(2)	54(2)	-2(1)	18(1)	12(1)
C(23)	46(2)	27(1)	69(2)	0(1)	21(1)	4(1)
C(22)	55(2)	36(1)	62(2)	-8(1)	24(2)	2(1)
C(24)	35(1)	30(1)	60(2)	3(1)	14(1)	3(1)
C(25)	30(1)	19(1)	61(2)	1(1)	16(1)	4(1)
C(26)	40(1)	27(1)	68(2)	-1(1)	14(1)	5(1)
C(28)	56(2)	43(2)	82(2)	-8(2)	40(2)	-12(1)
C(27)	57(2)	37(1)	60(2)	0(1)	24(2)	0(1)
C(29)	39(2)	38(1)	79(2)	-7(1)	26(2)	-6(1)
C(30)	35(1)	26(1)	68(2)	-3(1)	19(1)	1(1)
C(31)	48(2)	47(2)	40(1)	-2(1)	3(1)	8(1)
C(32)	98(3)	51(2)	83(3)	-18(2)	30(2)	-10(2)
C(33)	118(4)	46(2)	79(3)	-1(2)	24(2)	-3(2)
C(34)	105(3)	35(2)	118(3)	2(2)	65(3)	9(2)
C(35)	75(3)	58(2)	111(3)	-3(2)	30(2)	11(2)
C(36)	67(2)	60(2)	125(4)	-19(2)	16(2)	15(2)
C(37)	43(2)	44(2)	42(1)	-2(1)	8(1)	-1(1)
C(38)	71(2)	89(3)	58(2)	-7(2)	15(2)	-29(2)
C(39)	65(2)	64(2)	46(2)	0(2)	-2(2)	-4(2)
C(40)	75(2)	50(2)	44(2)	-1(1)	17(2)	3(2)
C(41)	68(2)	102(3)	54(2)	13(2)	16(2)	-17(2)
C(42)	56(2)	74(2)	54(2)	0(2)	14(2)	-2(2)
C(43)	49(2)	74(2)	43(2)	-4(2)	7(1)	-10(2)
C(44)	60(2)	73(2)	59(2)	5(2)	22(2)	1(2)
C(45)	67(2)	97(3)	72(2)	-6(2)	29(2)	-5(2)
C(46)	65(2)	92(3)	80(3)	-18(2)	28(2)	-10(2)
C(47)	54(2)	96(3)	70(2)	-9(2)	4(2)	6(2)
C(48)	58(2)	65(2)	62(2)	-6(2)	8(2)	2(2)
C(100)	39(1)	60(2)	42(1)	4(1)	5(1)	2(1)
C(101)	39(1)	60(2)	42(1)	4(1)	5(1)	2(1)
C(102)	39(1)	61(2)	43(1)	4(1)	5(1)	2(1)

C(103)	39(1)	61(2)	44(1)	4(1)	6(1)	3(1)
C(104)	39(1)	61(2)	44(1)	4(1)	5(1)	3(1)
C(105)	39(1)	61(2)	43(1)	4(1)	5(1)	3(1)
C(106)	39(1)	60(2)	42(1)	4(1)	5(1)	2(1)
C(107)	39(1)	60(2)	43(1)	4(1)	5(1)	2(1)
C(108)	39(1)	61(2)	44(1)	4(1)	6(1)	2(1)
C(109)	39(1)	61(2)	44(1)	4(1)	5(1)	3(1)
C(110)	39(1)	61(2)	44(1)	4(1)	5(1)	3(1)
C(111)	39(1)	61(2)	43(1)	4(1)	5(1)	3(1)
C(55)	48(2)	49(2)	56(2)	3(1)	-3(1)	5(1)
C(56)	77(2)	56(2)	47(2)	8(1)	-8(2)	6(2)
C(58)	105(4)	105(4)	93(4)	-14(3)	-49(3)	47(3)
C(59)	68(3)	102(3)	106(4)	-18(3)	-27(3)	37(2)
C(60)	54(2)	81(3)	82(3)	-3(2)	-2(2)	17(2)
C(61)	26(1)	34(1)	48(1)	0(1)	5(1)	0(1)
C(62)	29(1)	33(1)	63(2)	-3(1)	-1(1)	1(1)
C(63)	32(1)	40(2)	73(2)	-2(1)	-8(1)	2(1)
C(64)	26(1)	44(2)	100(3)	-11(2)	-1(1)	5(1)
C(65)	25(1)	50(2)	87(2)	-17(2)	10(1)	5(1)
C(66)	26(1)	38(1)	71(2)	-13(1)	11(1)	6(1)
C(67)	27(1)	34(1)	58(2)	-3(1)	18(1)	4(1)
C(68)	31(1)	49(2)	66(2)	9(1)	18(1)	5(1)
C(69)	43(2)	69(2)	75(2)	24(2)	26(2)	11(2)
C(70)	51(2)	52(2)	90(3)	18(2)	40(2)	8(1)
C(71)	49(2)	41(2)	90(2)	-5(2)	36(2)	-5(1)
C(72)	35(1)	37(1)	72(2)	-6(1)	21(1)	-3(1)
C(73)	29(1)	31(1)	47(1)	-1(1)	6(1)	5(1)
C(74)	32(1)	39(1)	48(2)	3(1)	6(1)	8(1)
C(75)	42(2)	56(2)	54(2)	2(1)	14(1)	15(1)
C(76)	61(2)	56(2)	54(2)	-10(1)	11(2)	17(2)
C(77)	54(2)	48(2)	58(2)	-11(1)	0(1)	5(1)
C(78)	35(1)	36(1)	56(2)	-5(1)	4(1)	3(1)
C(79)	33(1)	41(1)	46(2)	-4(1)	-3(1)	16(1)
C(80)	46(2)	62(2)	45(2)	2(1)	4(1)	19(1)
C(81)	76(2)	94(3)	46(2)	2(2)	10(2)	42(2)
C(82)	92(3)	88(3)	47(2)	-22(2)	-9(2)	49(2)

C(83)	72(2)	60(2)	72(2)	-29(2)	-17(2)	27(2)
C(84)	49(2)	43(2)	64(2)	-15(1)	-5(1)	14(1)
C(85)	22(1)	32(1)	46(1)	0(1)	2(1)	-3(1)
C(86)	24(1)	34(1)	54(2)	3(1)	1(1)	-2(1)
C(87)	37(1)	32(1)	73(2)	8(1)	-2(1)	-1(1)
C(88)	57(2)	52(2)	54(2)	14(1)	-8(2)	-14(2)
C(89)	51(2)	58(2)	48(2)	2(1)	3(1)	-14(2)
C(90)	34(1)	47(2)	49(2)	-2(1)	6(1)	-9(1)
C(57)	122(4)	78(3)	57(2)	9(2)	-23(2)	22(3)

3.8 Crystal Structure Determination [Y-K·(18-C-6)]

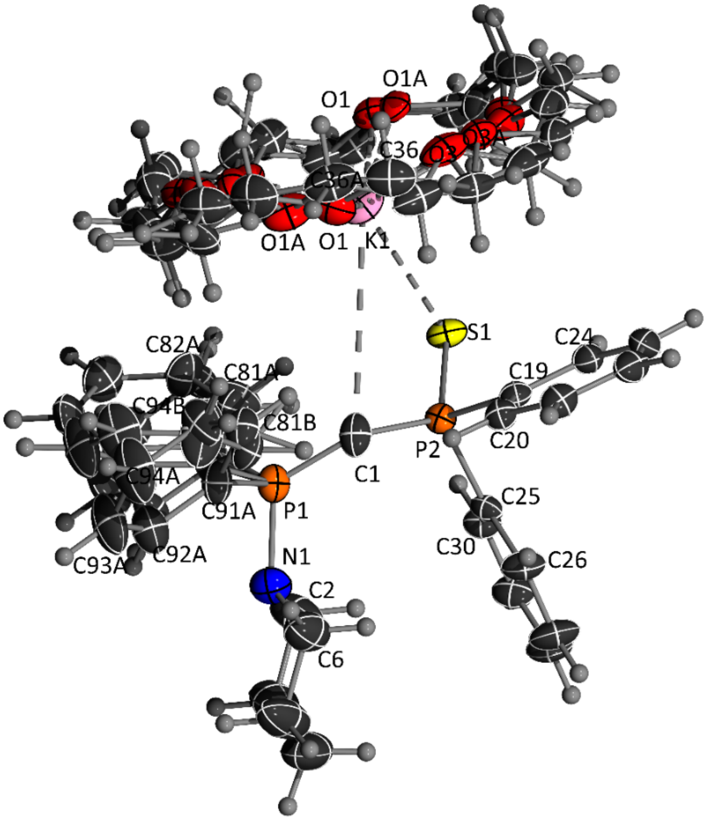

Figure s18 Molecular structure of **[Y-K·(18-C-6)]**. Thermal ellipsoids at 50% probability level. Selected bond lengths [Å] and angles [°]: P1-C1 1.609(3), P2-C1 1.660(2), K1-C1 3.857(4), N1-P1 1.729(2), S1-P2 2.0192(8), S1-K1 3.1573(12), P1-C1-P2 139.59(16).

Table s16 Atomic coordinates (x 10^4) and equivalent isotropic displacement parameters (Å^{2}x 10^3) for compound **[Y-K·(18-C-6)]**. U(eq) is defined as one third of the trace of the orthogonalized Uij tensor.

	x	y	z	U(eq)
K(1)	3182(1)	3259(1)	6827(1)	39(1)
O(1)	4244(8)	1080(7)	6523(3)	36(1)
N(1)	7739(3)	8686(2)	8741(1)	42(1)
S(1)	6396(1)	4813(1)	6655(1)	31(1)
P(1)	6309(1)	7233(1)	8387(1)	32(1)
C(1)	5639(3)	6800(3)	7608(1)	36(1)
P(2)	6210(1)	6534(1)	6937(1)	24(1)
O(2)	2866(5)	2010(4)	5492(2)	36(1)
C(2)	9234(3)	8762(4)	8670(2)	66(1)
C(3)	10442(4)	9944(4)	9125(2)	75(1)
O(3)	779(3)	3152(3)	5684(2)	33(1)
C(4)	10114(6)	11141(4)	9007(2)	98(1)
O(4)	818(4)	4066(3)	6978(2)	45(1)
C(5)	8566(6)	11019(3)	9049(2)	83(1)
O(5)	2016(4)	2981(4)	7988(2)	48(1)
C(6)	7441(5)	9816(3)	8588(2)	62(1)
O(6)	4321(9)	2072(8)	7795(3)	46(1)
C(19)	4901(2)	6797(2)	6261(1)	25(1)
C(20)	3998(2)	7497(2)	6399(1)	28(1)
C(21)	3064(3)	7771(2)	5894(1)	35(1)
C(22)	3006(3)	7346(2)	5250(1)	37(1)
C(23)	3870(3)	6616(2)	5107(1)	37(1)
C(24)	4821(2)	6361(2)	5611(1)	29(1)
C(25)	7977(2)	7768(2)	6895(1)	25(1)
C(26)	8031(3)	8990(2)	6783(1)	37(1)
C(27)	9377(3)	9936(3)	6785(2)	48(1)
C(28)	10692(3)	9672(3)	6896(2)	45(1)
C(29)	10663(3)	8472(3)	7007(1)	39(1)
C(30)	9318(2)	7525(2)	7007(1)	32(1)
C(80A)	6805(4)	6064(4)	8845(2)	26(1)
C(81A)	7322(6)	5236(5)	8502(2)	44(2)
C(82A)	7697(6)	4280(5)	8789(2)	47(2)

C(83A)	7556(5)	4153(3)	9420(2)	55(2)
C(84A)	7040(5)	4982(3)	9764(1)	52(1)
C(85A)	6664(5)	5937(3)	9476(2)	48(1)
C(85B)	8142(6)	6637(4)	9437(2)	38(2)
C(84B)	8868(6)	5868(4)	9727(2)	56(2)
C(83B)	8777(6)	4712(4)	9369(2)	41(2)
C(82B)	7960(8)	4325(5)	8720(2)	46(3)
C(81B)	7235(7)	5093(6)	8430(2)	39(3)
C(80B)	7326(5)	6250(5)	8788(2)	24(2)
C(90A)	6620(6)	2826(6)	11622(2)	38(2)
C(91A)	5164(5)	2414(5)	11233(2)	38(2)
C(92A)	4847(3)	1814(4)	10586(2)	51(1)
C(93A)	5986(4)	1626(5)	10328(1)	69(1)
C(94A)	7442(4)	2037(5)	10717(2)	73(2)
C(95A)	7759(4)	2637(5)	11364(2)	51(2)
C(95B)	7532(10)	2518(12)	11330(4)	73(6)
C(94B)	7554(7)	2796(9)	10708(4)	61(3)
C(93B)	6368(8)	3068(9)	10345(3)	69(1)
C(92B)	5160(7)	3062(9)	10605(3)	67(3)
C(91B)	5139(9)	2785(9)	11228(3)	27(2)
C(90B)	6325(12)	2513(12)	11590(3)	37(3)
C(31)	4670(6)	1117(5)	5918(3)	40(1)
C(32)	3339(6)	943(5)	5378(3)	39(1)
C(33)	1669(5)	1968(5)	4967(2)	39(1)
C(34)	1312(5)	3166(5)	5104(2)	37(1)
C(35)	469(5)	4289(4)	5867(3)	42(1)
C(36)	-252(6)	4102(5)	6434(3)	46(1)
C(37)	206(6)	3862(5)	7532(3)	55(1)
C(38)	1450(8)	3980(6)	8113(3)	57(1)
C(39)	3264(8)	3087(7)	8496(3)	62(2)
C(40)	3778(7)	1959(8)	8358(3)	59(1)
C(41)	4966(8)	1141(7)	7665(3)	51(2)
C(42)	5498(7)	1310(8)	7054(3)	43(1)
O(4A)	1026(8)	3969(7)	7349(5)	48(2)
C(37A)	708(13)	3691(11)	7946(7)	50(2)
C(38A)	2127(14)	3740(10)	8410(6)	55(2)

O(5A)	2540(11)	2713(7)	8142(4)	42(2)
C(39A)	3813(14)	2637(11)	8576(5)	45(2)
C(40A)	4201(14)	1543(11)	8283(5)	47(2)
O(6A)	4537(19)	1873(15)	7683(6)	46(3)
C(41A)	5055(17)	936(15)	7418(7)	43(3)
C(42A)	5381(17)	1231(15)	6791(7)	37(2)
O(1A)	4035(18)	1090(15)	6342(5)	35(2)
C(31A)	4221(16)	1215(13)	5705(6)	41(2)
C(32A)	2829(15)	1233(13)	5270(6)	54(3)
O(2A)	2528(11)	2339(10)	5500(5)	49(2)
C(33A)	1361(13)	2549(14)	5072(6)	53(3)
C(34A)	1092(11)	3698(12)	5399(6)	55(2)
O(3A)	569(7)	3416(8)	5975(5)	47(2)
C(35A)	210(13)	4401(11)	6299(7)	49(3)
C(36A)	-231(10)	4004(10)	6903(6)	52(2)

Table s17 Anisotropic displacement parameters ($Å^2$x 10^3) for compound **[Y-K·(18-C-6)]**. The anisotropic displacement factor exponent takes the form: $-2p^2[h^2a^{*2}U^{11} + ... + 2 h k a^* b^* U^{12}]$.

	U^{11}	U^{22}	U^{33}	U^{23}	U^{13}	U^{12}
K(1)	24(1)	42(1)	58(1)	17(1)	18(1)	13(1)
O(1)	30(2)	32(2)	48(3)	6(2)	14(2)	11(1)
N(1)	41(1)	38(1)	49(1)	5(1)	13(1)	17(1)
S(1)	24(1)	23(1)	46(1)	5(1)	9(1)	8(1)
P(1)	31(1)	38(1)	32(1)	10(1)	9(1)	18(1)
C(1)	32(1)	53(2)	34(1)	14(1)	14(1)	24(1)
P(2)	20(1)	27(1)	27(1)	4(1)	7(1)	10(1)
O(2)	30(2)	35(2)	40(2)	6(1)	8(1)	9(1)
C(2)	38(1)	79(2)	64(2)	-21(2)	14(1)	8(1)
C(3)	54(2)	81(2)	62(2)	-9(2)	8(2)	-3(1)
O(3)	27(1)	30(2)	42(2)	8(1)	7(1)	9(1)
C(4)	101(2)	65(2)	74(2)	14(2)	-13(2)	-21(2)
O(4)	34(2)	47(2)	52(2)	-4(2)	12(2)	16(1)
C(5)	119(2)	39(1)	68(2)	5(1)	-18(2)	23(2)
O(5)	40(2)	54(2)	41(2)	-9(2)	10(2)	12(2)

C(6)	78(2)	40(1)	59(2)	10(1)	-1(2)	20(1)
O(6)	40(2)	58(3)	36(2)	10(2)	6(2)	11(2)
C(19)	18(1)	23(1)	29(1)	4(1)	5(1)	3(1)
C(20)	24(1)	30(1)	32(1)	6(1)	8(1)	10(1)
C(21)	27(1)	36(1)	44(1)	12(1)	7(1)	14(1)
C(22)	33(1)	34(1)	38(1)	11(1)	-1(1)	7(1)
C(23)	42(1)	31(1)	29(1)	3(1)	3(1)	6(1)
C(24)	28(1)	27(1)	31(1)	4(1)	8(1)	6(1)
C(25)	22(1)	24(1)	26(1)	-3(1)	6(1)	7(1)
C(26)	26(1)	26(1)	55(2)	4(1)	6(1)	9(1)
C(27)	38(1)	25(1)	75(2)	9(1)	10(1)	4(1)
C(28)	27(1)	34(1)	62(2)	4(1)	10(1)	-1(1)
C(29)	23(1)	40(1)	51(2)	1(1)	9(1)	9(1)
C(30)	24(1)	30(1)	40(1)	2(1)	8(1)	11(1)
C(80A)	22(3)	29(2)	27(2)	6(2)	5(2)	9(2)
C(81A)	52(6)	44(4)	44(4)	20(3)	12(4)	22(4)
C(82A)	60(4)	44(4)	52(4)	20(3)	18(3)	31(3)
C(83A)	67(4)	52(3)	55(3)	22(2)	10(3)	33(3)
C(84A)	83(4)	47(3)	30(2)	12(2)	5(2)	29(3)
C(85A)	76(4)	44(3)	30(2)	6(2)	9(2)	32(3)
C(85B)	44(3)	42(3)	29(3)	0(2)	1(2)	23(3)
C(84B)	78(5)	51(4)	33(3)	-2(3)	-16(3)	36(4)
C(83B)	50(4)	39(3)	40(3)	9(3)	3(3)	29(3)
C(82B)	57(5)	39(5)	40(5)	-6(4)	4(4)	24(4)
C(81B)	37(6)	38(5)	39(5)	-9(4)	-2(4)	21(5)
C(80B)	22(4)	27(3)	25(3)	10(2)	10(3)	5(3)
C(90A)	28(3)	53(4)	39(3)	15(2)	13(2)	17(3)
C(91A)	42(3)	54(4)	34(3)	16(2)	18(2)	28(2)
C(92A)	53(3)	69(3)	38(2)	9(2)	12(2)	29(2)
C(93A)	68(3)	115(4)	38(2)	9(3)	26(2)	44(3)
C(94A)	67(4)	125(6)	53(3)	12(4)	31(3)	61(4)
C(95A)	41(3)	72(5)	46(4)	-1(3)	23(3)	25(3)
C(95B)	40(6)	137(16)	72(11)	55(10)	20(6)	54(8)
C(94B)	50(5)	100(9)	57(6)	10(6)	29(4)	52(6)
C(93B)	68(3)	115(4)	38(2)	9(3)	26(2)	44(3)
C(92B)	49(5)	134(11)	37(4)	27(5)	19(4)	50(6)

C(91B)	27(4)	38(5)	23(4)	6(3)	5(3)	23(3)
C(90B)	36(6)	44(6)	41(6)	17(4)	21(4)	17(5)
C(31)	35(3)	32(2)	56(3)	4(2)	21(2)	12(2)
C(32)	37(3)	34(2)	47(3)	1(2)	17(2)	10(2)
C(33)	34(2)	38(2)	37(2)	2(2)	10(2)	3(2)
C(34)	31(2)	35(2)	37(2)	9(2)	4(2)	3(2)
C(35)	33(2)	29(2)	63(3)	11(2)	5(2)	12(2)
C(36)	33(3)	40(3)	68(3)	1(2)	13(2)	20(2)
C(37)	53(3)	55(3)	66(3)	-1(2)	29(3)	25(2)
C(38)	60(4)	52(3)	50(3)	-15(2)	16(3)	14(3)
C(39)	52(3)	86(4)	38(3)	-9(3)	8(2)	18(3)
C(40)	49(3)	81(4)	41(3)	12(3)	6(2)	19(3)
C(41)	39(2)	50(3)	57(3)	19(3)	0(3)	10(2)
C(42)	30(2)	40(2)	62(4)	13(3)	8(3)	18(2)
O(4A)	31(3)	40(3)	78(5)	14(4)	17(4)	15(3)
C(37A)	38(5)	40(5)	72(6)	-3(5)	20(5)	16(4)
C(38A)	55(5)	45(5)	62(5)	-4(4)	20(4)	15(4)
O(5A)	38(4)	35(3)	50(5)	-3(3)	13(4)	9(3)
C(39A)	44(5)	36(5)	43(5)	3(4)	5(4)	1(4)
C(40A)	45(5)	47(5)	47(5)	12(4)	9(4)	14(4)
O(6A)	43(5)	43(5)	44(4)	11(4)	16(3)	0(4)
C(41A)	35(4)	45(5)	55(6)	21(5)	4(5)	20(4)
C(42A)	30(5)	33(4)	58(6)	16(5)	16(5)	17(3)
O(1A)	31(5)	31(4)	42(5)	7(4)	19(4)	4(3)
C(31A)	41(6)	40(5)	41(5)	9(4)	21(4)	6(4)
C(32A)	47(6)	47(6)	47(5)	7(5)	5(5)	-10(4)
O(2A)	33(4)	47(5)	52(4)	24(4)	-6(3)	1(3)
C(33A)	40(5)	50(6)	46(5)	15(5)	-4(4)	-5(5)
C(34A)	34(4)	46(5)	72(6)	25(5)	-2(4)	0(4)
O(3A)	23(3)	39(4)	74(5)	22(4)	6(3)	6(2)
C(35A)	27(5)	36(5)	81(6)	24(4)	2(4)	11(4)
C(36A)	32(4)	41(4)	87(6)	13(4)	15(4)	16(3)

4. Computational Details

General. All calculations were performed without symmetry restrictions using the Gaussian16 Revision C.01[4] program packages. Starting coordinates were directly obtained from the crystal structure analyses and optimized using the PW6B95 functional[5] with Grimme's D3 dispersion correction and Becke-Johnson damping[6] and the def2tzvp basis set[7] implemented in Gaussian. Frequency calculations were performed on the same level of theory and showed no imaginary frequency. NBO analysis was performed with NBO Version 7.[8]

Results of the NBO analyses

	Y-H	Y-Li		Y-K	
**NBO**	Population	Population	Δ(PopYLi-PopYH)	Population	Δ(PopYK-PopYH)
P2-C1	0,08898	0,05324	-0,03574	0,06633	-0,02265
P2-S1	0,02973	0,08486	0,05513	0,11605	0,08632
P2-C	0,15305	0,16308	0,01003	0,16434	0,01129
P2-C	0,15131	0,15684	0,00553	0,15729	0,00598
P1-C1	0,05331	0,03228	-0,02103	0,05195	-0,00136
P1-N1	0,1449	0,18642	0,04152	0,20627	0,06137
P1-C	0,08538	0,12371	0,03833	0,12289	0,03751
P1-C	0,11711	0,1342	0,01709	0,1659	0,04879
**Wiberg bond indices**	Bond index	Bond index	Δ(wbiYLi-wbiYH)	Bond index	Δ(wbiYK-wbiYH)
P2-C1	0,9953	1,0975	0,1022	1,208	0,2127
P2-S1	1,3057	1,0435	-0,2622	1,1216	-0,1841
P2-C	0,8214	0,8976	0,0762	0,8006	-0,0208
P2-C	0,8278	0,9031	0,0753	0,8046	-0,0232
P1-C1	1,1698	1,1564	-0,0134	1,4178	0,248
P1-N1	0,8002	0,8494	0,0492	0,7175	-0,0827
P1-C	0,8717	0,9164	0,0447	0,8325	-0,0392
P1-C	0,859	0,9108	0,0518	0,7939	-0,0651
**Natural charges**	charge	charge	Δ(chargeYLi-wbiYH)	charge	Δ(chargeYK-wbiYH)
C1	-1,3668	-1,6239	-0,2571	-1,5534	-0,1866
P2	1,36768	1,41709	0,04941	1,37108	0,0034
S1	-0,64993	-0,7958	-0,14587	-0,79314	-0,14321
P2-C	-1,43439	-1,45363	-0,01924	-1,46282	-0,02843
P2-C	-1,43072	-1,43167	-0,00095	-1,4781	-0,04738
P1	1,79686	1,77217	-0,02469	1,72534	-0,07152
N1	-0,87395	-0,86925	0,0047	-0,87638	-0,00243
P1-C	-1,41738	-1,4481	-0,03072	-1,46182	-0,04444
P1-C	-1,41303	-1,45781	-0,04478	-1,49181	-0,07878

Coordinates

**YH**				
E=-2300.58530394				
1	S	1,250596	-0,006442	-2,2282
2	P	-1,249522	0,132611	0,195914
3	N	-1,736656	-1,130013	-0,807595
4	C	0,349661	0,144007	0,755707
5	H	0,484283	-0,040746	1,820616
6	P	1,715487	0,012748	-0,309892
7	C	-1,152957	-2,431098	-0,492182
8	H	-0,896814	-2,461319	0,573709
9	H	-0,204657	-2,543846	-1,047427
10	C	-2,114681	-3,552577	-0,847028
11	H	-2,988776	-3,499992	-0,181166
12	H	-1,627861	-4,517722	-0,656998
13	C	-2,569171	-3,450048	-2,295742
14	H	-3,314341	-4,22301	-2,52326
15	H	-1,709104	-3,636903	-2,956188
16	C	-3,118535	-2,060663	-2,586021
17	H	-3,376398	-1,958934	-3,64806
18	H	-4,042268	-1,895627	-2,010973
19	C	-2,098892	-0,997725	-2,213155
20	H	-1,193781	-1,107911	-2,83176
21	H	-2,492411	0,004397	-2,399111
22	C	-2,279129	-0,002552	1,690096
23	C	-1,990585	0,776987	2,814117
24	H	-1,137228	1,45232	2,797805
25	C	-2,793831	0,694144	3,943865
26	H	-2,564671	1,303878	4,815694
27	C	-3,890762	-0,163328	3,960835
28	H	-4,518188	-0,226817	4,847896
29	C	-4,184286	-0,935994	2,843514
30	H	-5,040805	-1,607384	2,853207
31	C	-3,382699	-0,854767	1,709039
32	H	-3,603111	-1,453347	0,827271
33	C	-1,853916	1,634872	-0,625329
34	C	-3,228751	1,862373	-0,739752
35	H	-3,935534	1,125667	-0,359168
36	C	-3,694609	3,030201	-1,329268
37	H	-4,765327	3,20437	-1,415762
38	C	-2,790256	3,978097	-1,800953
39	H	-3,155139	4,894535	-2,261234
40	C	-1,422852	3,758491	-1,677847
41	H	-0,714261	4,498932	-2,043191
42	C	-0,951148	2,591044	-1,087397
43	H	0,117907	2,413678	-0,996352
44	C	2,671192	-1,468001	0,188278
45	C	3,220269	-1,546671	1,470494
46	H	3,124586	-0,705189	2,157099
47	C	3,898336	-2,691333	1,869086
48	H	4,326091	-2,748722	2,868432
49	C	4,036122	-3,761767	0,988218
50	H	4,569905	-4,657477	1,300954

51	C	3,499158	-3,681746	-0,29169
52	H	3,614095	-4,51376	-0,984291
53	C	2,819257	-2,53566	-0,693323
54	H	2,399856	-2,444094	-1,695379
55	C	2,880822	1,368746	0,071133
56	C	4,155096	1,355631	-0,501973
57	H	4,446654	0,523493	-1,142182
58	C	5,035156	2,40193	-0,264933
59	H	6,02765	2,387899	-0,711761
60	C	4,64789	3,471876	0,540336
61	H	5,339498	4,292261	0,723806
62	C	3,378782	3,491802	1,105753
63	H	3,073303	4,328029	1,732313
64	C	2,49517	2,440993	0,871973
65	H	1,495301	2,442528	1,304403

**(YLi-THF)2**				
E=-5080.92125128				
1	S	-0,49289	-0,622124	1,877727
2	P	-4,08569	0,572608	-0,832611
3	P	-2,361719	-0,987629	1,136022
4	N	-4,439702	-0,205676	-2,319552
5	Li	-0,904152	1,102473	0,045208
6	C	-2,703528	0,069887	-0,109946
7	C	-5,499721	0,331712	-3,154919
8	H	-5,127776	1,188478	-3,74907
9	H	-6,304496	0,71802	-2,51657
10	C	-6,027323	-0,748459	-4,086756
11	H	-6,80425	-0,328282	-4,738579
12	H	-6,504218	-1,53296	-3,480695
13	C	-4,898149	-1,352991	-4,911149
14	H	-4,513793	-0,588221	-5,603404
15	H	-5,271126	-2,177508	-5,532398
16	C	-3,760469	-1,819245	-4,011903
17	H	-4,093707	-2,667379	-3,395776
18	H	-2,909156	-2,16926	-4,610578
19	C	-3,310596	-0,698655	-3,091898
20	H	-2,539348	-1,029156	-2,38881
21	H	-2,864185	0,117562	-3,693497
22	C	-3,967106	2,342141	-1,280471
23	C	-5,02003	3,251463	-1,162997
24	H	-5,989945	2,919969	-0,797193
25	C	-4,834671	4,590037	-1,499298
26	H	-5,658085	5,293214	-1,386788
27	C	-3,604836	5,027375	-1,97828
28	H	-3,461167	6,074835	-2,237436
29	C	-2,562512	4,117645	-2,137107
30	H	-1,600182	4,446816	-2,524466
31	C	-2,741324	2,784902	-1,791791
32	H	-1,914748	2,083719	-1,908907
33	C	-5,64129	0,381424	0,094712

34	C	-5,866352	1,189285	1,214477
35	H	-5,157678	1,979987	1,459396
36	C	-6,980469	0,983699	2,015729
37	H	-7,14377	1,615621	2,886291
38	C	-7,873392	-0,042667	1,718794
39	H	-8,744366	-0,205917	2,350872
40	C	-7,641356	-0,867882	0,624915
41	H	-8,33078	-1,678842	0,397004
42	C	-6,529386	-0,658354	-0,184762
43	H	-6,341834	-1,301026	-1,043295
44	C	-2,451947	-2,759793	0,663907
45	C	-3,348153	-3,142852	-0,335127
46	H	-3,95697	-2,383455	-0,827325
47	C	-3,449242	-4,478904	-0,709444
48	H	-4,151848	-4,772636	-1,48792
49	C	-2,646218	-5,43651	-0,095425
50	H	-2,718834	-6,481062	-0,393532
51	C	-1,744099	-5,055042	0,894167
52	H	-1,107616	-5,800802	1,36783
53	C	-1,648491	-3,720851	1,275512
54	H	-0,938619	-3,404339	2,038097
55	C	-3,528568	-0,913245	2,552007
56	C	-4,620208	-1,778369	2,641144
57	H	-4,75852	-2,560681	1,897808
58	C	-5,533859	-1,652462	3,682062
59	H	-6,379641	-2,335216	3,740589
60	C	-5,372894	-0,657309	4,638898
61	H	-6,087371	-0,561681	5,454744
62	C	-4,291474	0,215293	4,550657
63	H	-4,15367	0,991776	5,301754
64	C	-3,372617	0,085002	3,516503
65	H	-2,506017	0,741229	3,460388
66	C	0,178656	2,96233	1,810417
67	H	0,695354	3,906374	1,583139
68	H	0,871162	2,133883	1,621885
69	C	-0,421312	2,949427	3,199065
70	H	-0,589383	1,909702	3,506499
71	H	0,219766	3,433966	3,943275
72	C	-1,741468	3,677671	2,970402
73	H	-1,585802	4,763539	2,996484
74	H	-2,507023	3,432808	3,71542
75	C	-2,141473	3,225876	1,570234
76	H	-2,826274	2,366819	1,583652
77	H	-2,604387	4,023003	0,973364
78	S	0,494077	0,624477	-1,879903
79	P	4,084488	-0,572118	0,833063
80	P	2,362776	0,988551	-1,136948
81	N	4,43787	0,205627	2,320439
82	Li	0,904333	-1,101928	-0,048476
83	C	2,702943	-0,068113	0,110059
84	C	5,496269	-0,333063	3,156988
85	H	5,122526	-1,189322	3,750732
86	H	6,30127	-0,7205	2,519627
87	C	6,024208	0,746426	4,089475

88	H	6,799941	0,325241	4,742065
89	H	6,502712	1,530426	3,484027
90	C	4,894921	1,352291	4,912743
91	H	4,508906	0,58792	5,604517
92	H	5,268211	2,176302	5,534469
93	C	3,758782	1,819864	4,012276
94	H	4,093667	2,667648	3,396516
95	H	2,907255	2,170845	4,610075
96	C	3,308572	0,699974	3,09159
97	H	2,538635	1,031613	2,387611
98	H	2,860299	-0,115694	3,692532
99	C	3,96417	-2,341463	1,280952
100	C	5,016791	-3,251381	1,165319
101	H	5,987348	-2,920604	0,800585
102	C	4,830271	-4,589655	1,502193
103	H	5,653478	-5,293313	1,391222
104	C	3,599529	-5,026119	1,979668
105	H	3,454972	-6,073359	2,239222
106	C	2,55744	-4,115792	2,136578
107	H	1,594374	-4,444265	2,522777
108	C	2,737456	-2,783305	1,79086
109	H	1,911245	-2,081541	1,906818
110	C	5,641042	-0,381941	-0,093102
111	C	5,867358	-1,190971	-1,211767
112	H	5,15913	-1,982152	-1,456427
113	C	6,982122	-0,986	-2,012305
114	H	7,146301	-1,618806	-2,882069
115	C	7,874498	0,04096	-1,715789
116	H	8,745925	0,203795	-2,347349
117	C	7,64123	0,867377	-0,623086
118	H	8,330096	1,678938	-0,395614
119	C	6,528588	0,658484	0,185799
120	H	6,339784	1,302496	1,043035
121	C	2,455198	2,760655	-0,665037
122	C	3,352422	3,142154	0,333711
123	H	3,960191	2,381614	0,825601
124	C	3,456129	4,478004	0,707942
125	H	4,159562	4,770446	1,486158
126	C	2,654552	5,437012	0,094224
127	H	2,729192	6,481451	0,392231
128	C	1,751435	5,05712	-0,895082
129	H	1,116252	5,80405	-1,368655
130	C	1,65338	3,723121	-1,276526
131	H	0,942932	3,407915	-2,039066
132	C	3,52983	0,912195	-2,552586
133	C	4,622489	1,776007	-2,642058
134	H	4,761814	2,558351	-1,898969
135	C	5,535921	1,648687	-3,683024
136	H	6,382654	2,330258	-3,741725
137	C	5,373522	0,653586	-4,639664
138	H	6,087703	0,556978	-5,455648
139	C	4,290976	-0,217598	-4,551157
140	H	4,152012	-0,993939	-5,302182
141	C	3,372444	-0,086026	-3,516914

142	H	2,504965	-0,741215	-3,460731
143	O	0,933469	-2,80103	-0,934822
144	C	-0,179053	-2,961061	-1,812394
145	H	-0,695985	-3,905158	-1,585667
146	H	-0,871555	-2,132617	-1,623749
147	C	0,421402	-2,947839	-3,200824
148	H	0,590217	-1,907932	-3,507306
149	H	-0,219606	-3,4314	-3,945746
150	C	1,741051	-3,676941	-2,972036
151	H	1,584733	-4,76272	-2,999319
152	H	2,507237	-3,431772	-3,71627
153	C	2,140422	-3,227165	-1,57103
154	H	2,826916	-2,369356	-1,58308
155	H	2,601255	-4,025648	-0,974322
156	O	-0,934143	2,801732	0,933251

**[Y(K-18C6)]**				
E=-3824.8959568				
1	K	-2,203784	-0,705354	-0,076277
2	O	-4,102995	-0,50567	-2,323836
3	N	3,54401	-0,024389	-0,041102
4	S	-1,089302	1,569453	-2,104107
5	P	1,912718	-0,476176	0,272372
6	C	0,647793	0,555639	0,432694
7	P	0,159538	1,84461	-0,526761
8	O	-4,410629	1,224636	-0,137694
9	C	3,995842	0,281919	-1,389612
10	H	3,893351	1,359928	-1,599262
11	H	3,367454	-0,245475	-2,113926
12	C	5,452395	-0,122313	-1,549905
13	H	5,531242	-1,213292	-1,43245
14	H	5,795742	0,123815	-2,563484
15	O	-3,502376	0,393918	2,3542
16	C	6,311899	0,577465	-0,506341
17	H	7,36044	0,260654	-0,582369
18	H	6,293681	1,660784	-0,704551
19	O	-1,852211	-1,857206	2,489374
20	C	5,77028	0,325465	0,894336
21	H	6,335767	0,901481	1,639185
22	H	5,88588	-0,737285	1,152967
23	O	-1,752285	-3,642497	0,36279
24	C	4,296696	0,696944	0,971666
25	H	3,889049	0,469196	1,96238
26	H	4,196258	1,784871	0,827048
27	O	-2,32017	-2,659286	-2,183912
28	C	-0,673637	3,055653	0,587784
29	C	-0,352839	3,065961	1,945919
30	H	0,360585	2,323371	2,305804
31	C	-0,953713	3,982194	2,805176
32	H	-0,698999	3,983814	3,864247
33	C	-1,881925	4,893891	2,310612

34	H	-2,355258	5,609606	2,981133
35	C	-2,204815	4,887336	0,955239
36	H	-2,931732	5,599111	0,56634
37	C	-1,601982	3,973781	0,096396
38	H	-1,855486	3,93864	-0,962171
39	C	1,575805	2,855741	-1,140014
40	C	2,456223	3,424275	-0,217398
41	H	2,265562	3,303383	0,848628
42	C	3,573788	4,128252	-0,650564
43	H	4,253696	4,568841	0,077255
44	C	3,825101	4,266917	-2,013556
45	H	4,702513	4,814492	-2,353694
46	C	2,94524	3,711416	-2,937044
47	H	3,133204	3,823925	-4,003782
48	C	1,82122	3,014556	-2,502831
49	H	1,114275	2,581841	-3,209625
50	C	1,741524	-1,776662	-1,037396
51	C	0,892868	-1,48058	-2,106255
52	H	0,331089	-0,544046	-2,106334
53	C	0,770594	-2,357899	-3,180425
54	H	0,111948	-2,096542	-4,007698
55	C	1,476323	-3,555488	-3,191286
56	H	1,382564	-4,243469	-4,030215
57	C	2,308646	-3,870632	-2,118853
58	H	2,861907	-4,808487	-2,115425
59	C	2,446783	-2,985326	-1,054925
60	H	3,111034	-3,245926	-0,236624
61	C	-4,594883	0,801395	-2,458172
62	H	-5,299585	0,859745	-3,307262
63	H	-3,769684	1,507033	-2,636131
64	C	-5,317069	1,18868	-1,200589
65	H	-5,779212	2,180207	-1,350847
66	H	-6,128807	0,468836	-0,992008
67	C	-4,949295	1,72314	1,050368
68	H	-5,802159	1,103721	1,380746
69	H	-5,314841	2,755733	0,911033
70	C	-3,880825	1,721856	2,102954
71	H	-3,022423	2,316354	1,7601
72	H	-4,271869	2,192909	3,021795
73	C	-2,469062	0,275496	3,291238
74	H	-2,711841	0,832112	4,213392
75	H	-1,52965	0,687216	2,884547
76	C	-2,29391	-1,177328	3,627901
77	H	-1,55971	-1,276768	4,446332
78	H	-3,252985	-1,593354	3,981706
79	C	-1,626316	-3,222953	2,687936
80	H	-2,581574	-3,752507	2,847893
81	H	-0,991076	-3,387285	3,574299
82	C	-0,924088	-3,778817	1,484316
83	H	0,028994	-3,246242	1,328699
84	H	-0,688666	-4,842362	1,666549
85	C	-1,184149	-4,204718	-0,788256
86	H	-1,008465	-5,285943	-0,644684
87	H	-0,213969	-3,737257	-1,005307

88	C	-2,115017	-4,022389	-1,950031
89	H	-1,665094	-4,50519	-2,835532
90	H	-3,078658	-4,52147	-1,745845
91	C	-3,114299	-2,411039	-3,308781
92	H	-4,076192	-2,945293	-3,219688
93	H	-2,611092	-2,77336	-4,223208
94	C	-3,369326	-0,937436	-3,437818
95	H	-2,417059	-0,386239	-3,509392
96	H	-3,938809	-0,763602	-4,36795
97	C	1,265633	-1,169672	2,891041
98	H	0,429826	-0,501432	2,693352
99	C	2,177101	-1,370766	1,855622
100	C	3,29598	-2,176452	2,08737
101	H	4,055248	-2,270075	1,314112
102	C	3,471952	-2,809533	3,313018
103	H	4,348065	-3,434024	3,479249
104	C	2,543448	-2,623929	4,332235
105	H	2,685722	-3,109377	5,296288
106	C	1,447876	-1,791628	4,121864
107	H	0,740793	-1,616252	4,93175

5. References

[1] P. J. Bailey, R. A. Coxall, C. M. Dick, S. Fabre, L. C. Henderson, C. Herber, S. T. Liddle, D. Loroño-González, A. Parkin, S. Parsons, *Chemistry* **2003**, *9*, 4820.

[2] V. H. Gessner, *Organometallics* **2011**, *30*, 4228.

[3] M. Fild, D. Bunke, D. Schomburg, *Z. Anorg. Allg. Chem.* **1988**, *566*, 90.

[4] M. J. Frisch, G. W. Trucks, H. B. Schlegel, G. E. Scuseria, M. A. Robb, J. R. Cheeseman, G. Scalmani, V. Barone, G. A. Petersson, H. Nakatsuji et al., *Gaussian 16, Revision C.01*, Gaussian, Inc., Wallingford CT, **2016**.

[5] Y. Zhao, D. G. Truhlar, *J. Phys. Chem. A* **2005**, *109*, 5656.

[6] a) S. Grimme, J. Antony, S. Ehrlich, H. Krieg, *J. Chem. Phys.* **2010**, *132*, 154104; b) S. Grimme, S. Ehrlich, L. Goerigk, *J. Comput. Chem.* **2011**, *32*, 1456; c) D. G. A. Smith, L. A. Burns, K. Patkowski, C. D. Sherrill, *J. Phys. Chem. Lett.* **2016**, *7*, 2197.

[7] F. Weigend, R. Ahlrichs, *Phys. Chem. Chem. Phys.* **2005**, *7*, 3297.

[8] E. D. Glendening, J, K. Badenhoop, A. E. Reed, J. E. Carpenter, J. A. Bohmann, C. M. Morales, P. Karafiloglou, C. R. Landis, and F. Weinhold, *NBO7*, Theoretical Chemistry Institute, University of Wisconsin, Madison, **2018**.
